# Supplementary figures and images for: DBT is a metabolic switch for maintenance of proteostasis under proteasomal impairment (part 1 of 2)
Source: eLife. 2024 Sep 10;12:RP91002. doi: 10.7554/eLife.91002 (PMC11386957; doi:10.7554/eLife.91002)

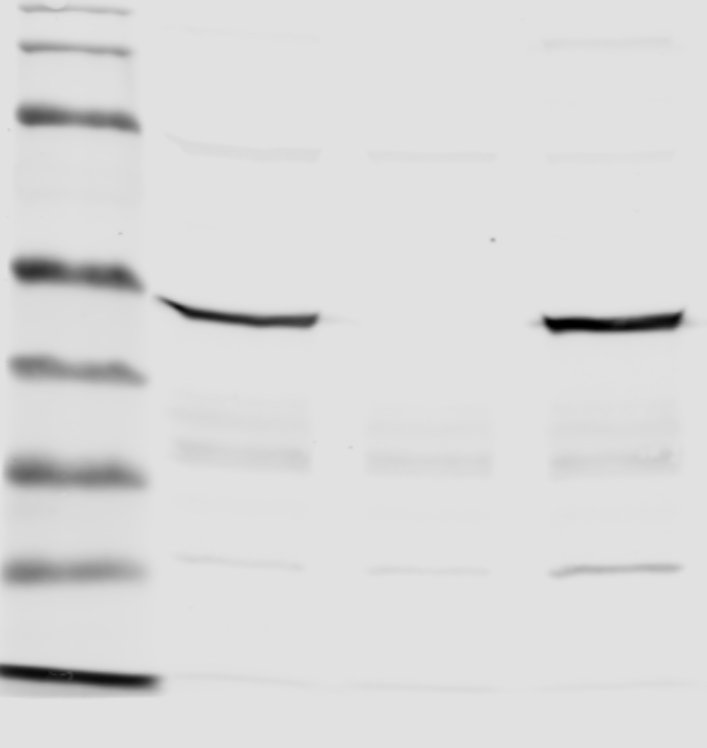

Supplement: Figure 1—source data 1. [file elife-91002-fig1-data1.zip › Figure 1 - Source data 1/Figure_1C- Source data_anti-DBT_raw data.tif]

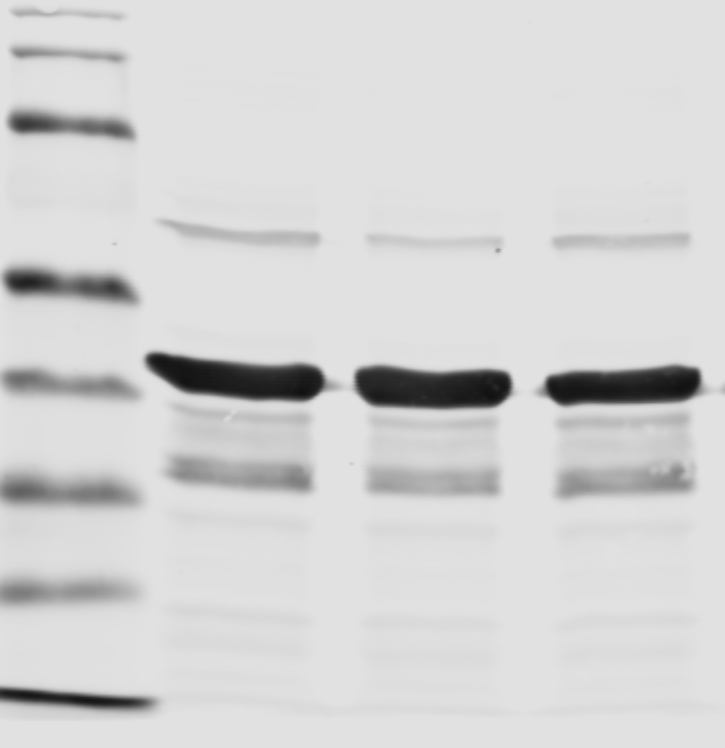

Supplement: Figure 1—source data 1. [file elife-91002-fig1-data1.zip › Figure 1 - Source data 1/Figure_1C- Source data_anti-Actin_raw data.tif]

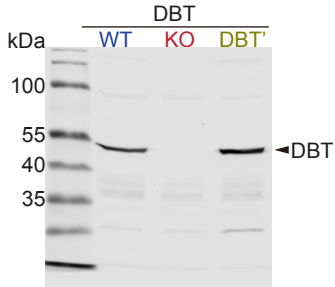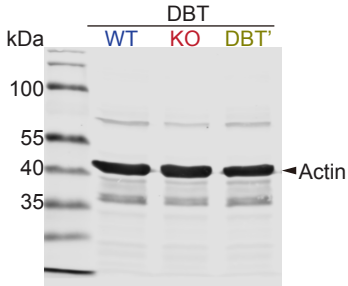

Supplement: Figure 1—source data 1. [file elife-91002-fig1-data1.zip › Figure 1 - Source data 1/Figure_1C_uncropped.pdf]

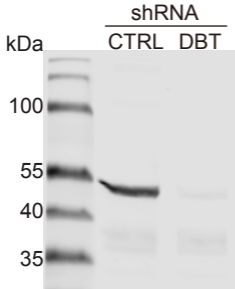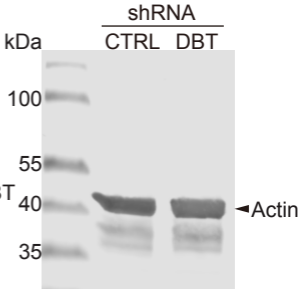

Supplement: Figure 1—source data 2. [file elife-91002-fig1-data2.zip › Figure 1 - Source data 2/Figure_1F_uncropped.pdf]

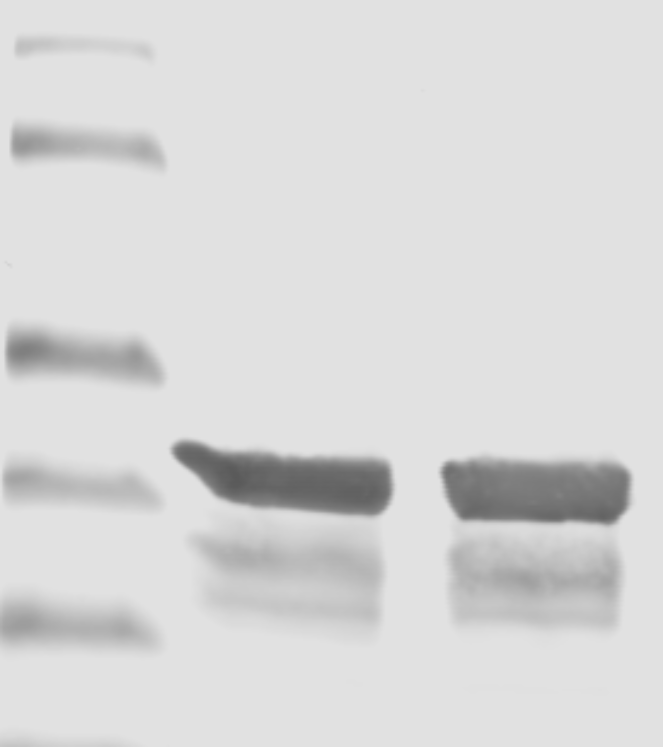

Supplement: Figure 1—source data 2. [file elife-91002-fig1-data2.zip › Figure 1 - Source data 2/Figure_1F- Source data_anti-Actin_raw data.tif]

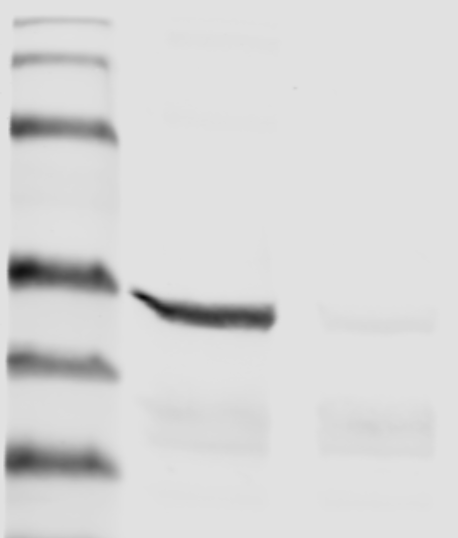

Supplement: Figure 1—source data 2. [file elife-91002-fig1-data2.zip › Figure 1 - Source data 2/Figure_1F- Source data_anti-DBT_raw data.tif]

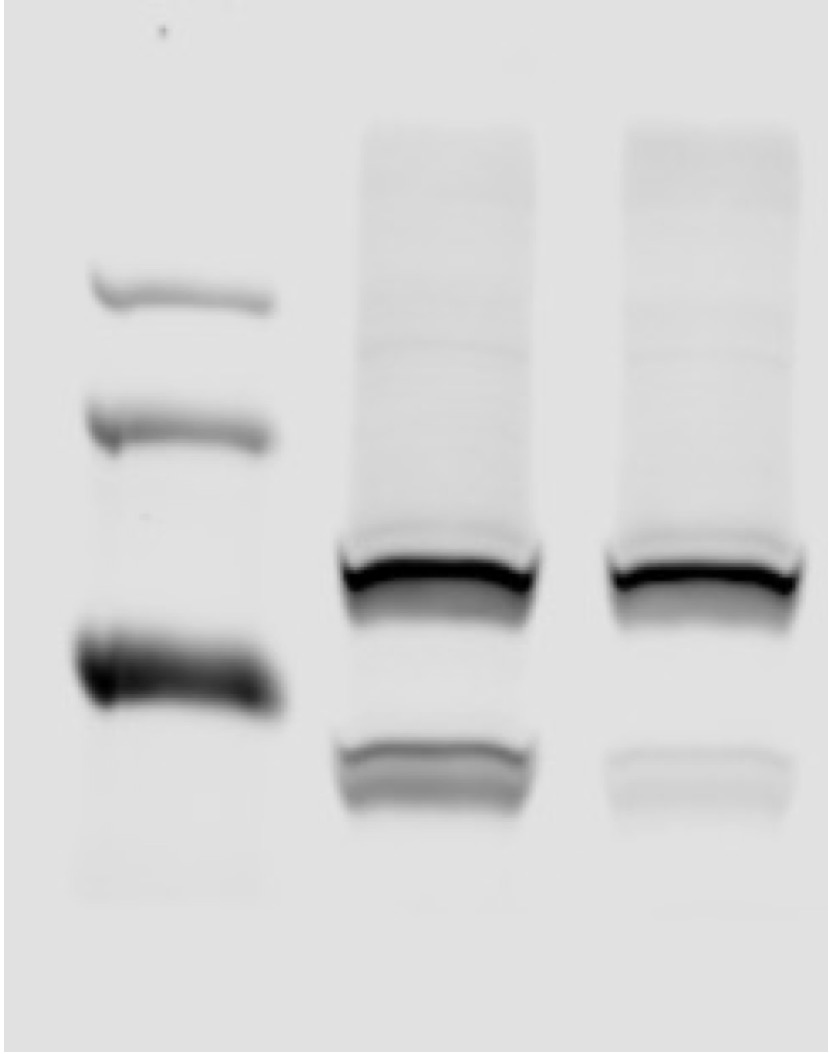

Supplement: Figure 1—source data 3. [file elife-91002-fig1-data3.zip › Figure 1 - Source data 3/Figure_1G- Source data_anti-PARP_raw data2.jpg]

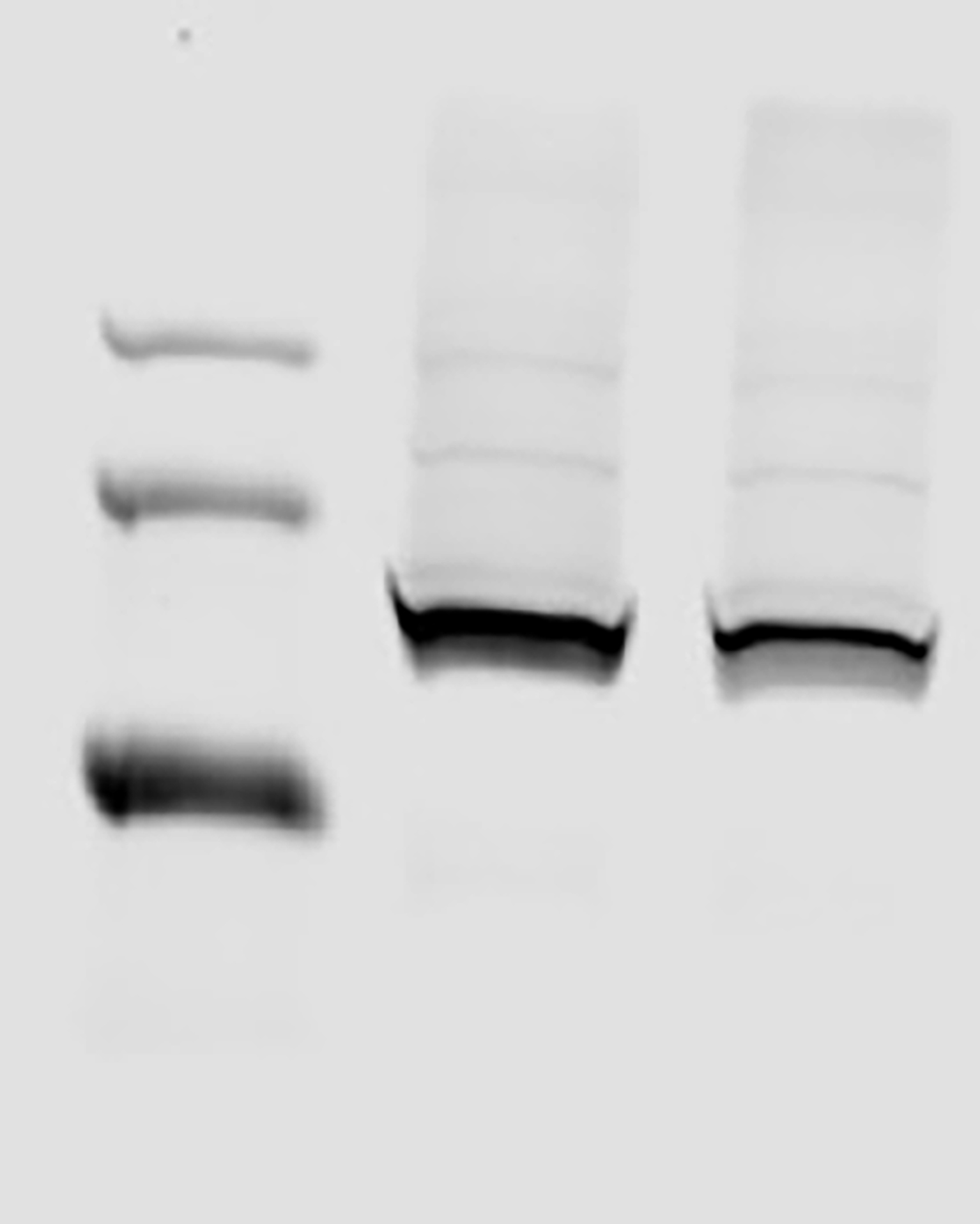

Supplement: Figure 1—source data 3. [file elife-91002-fig1-data3.zip › Figure 1 - Source data 3/Figure_1G- Source data_anti-PARP_raw data.jpg]

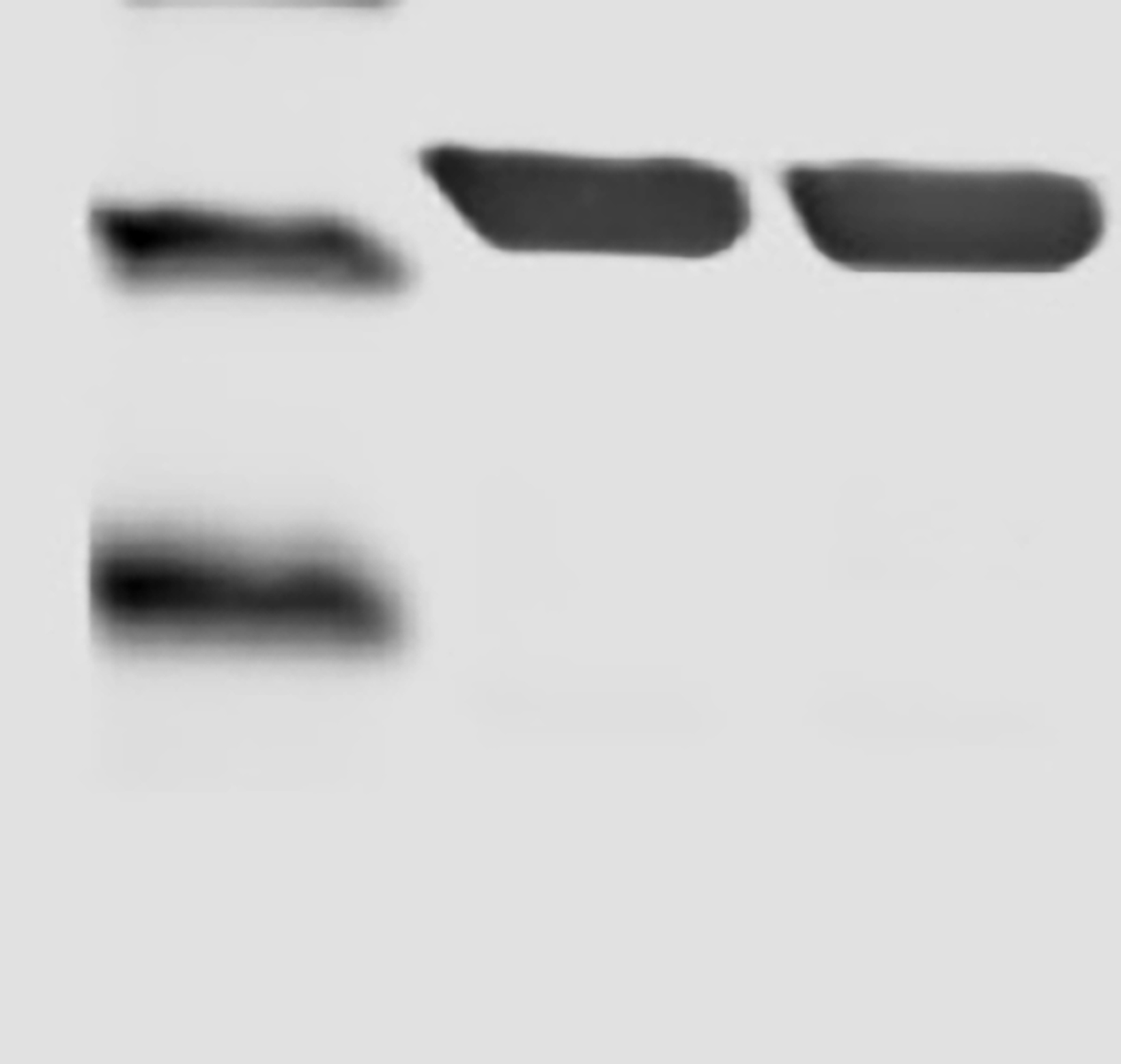

Supplement: Figure 1—source data 3. [file elife-91002-fig1-data3.zip › Figure 1 - Source data 3/Figure_1G- Source data_anti-Actin_raw data.jpg]

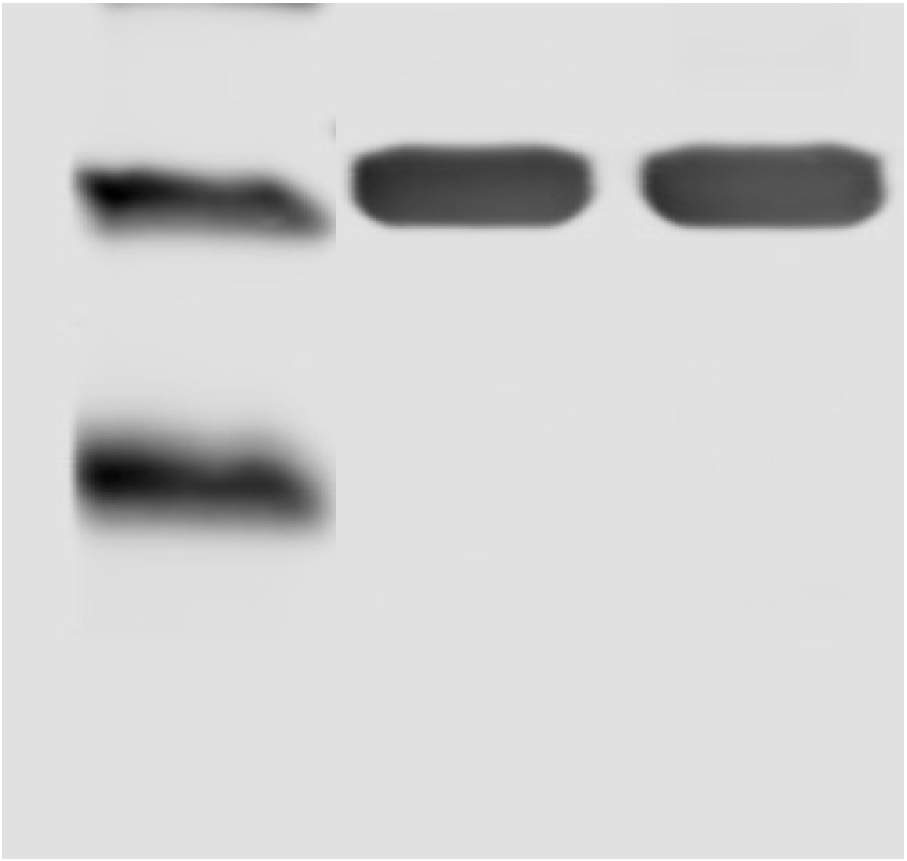

Supplement: Figure 1—source data 3. [file elife-91002-fig1-data3.zip › Figure 1 - Source data 3/Figure_1G- Source data_anti-Actin_raw data2.jpg]

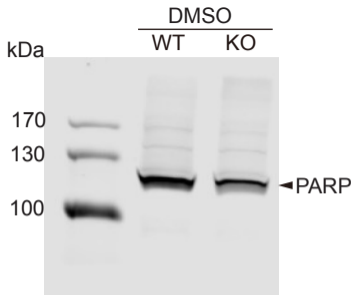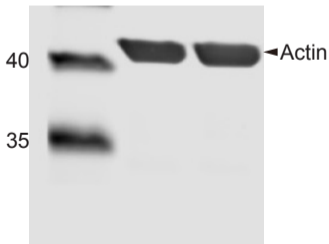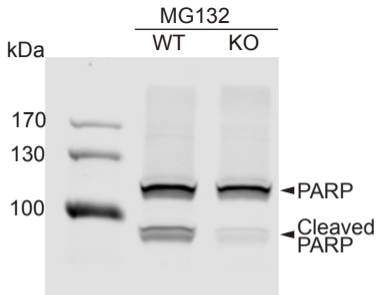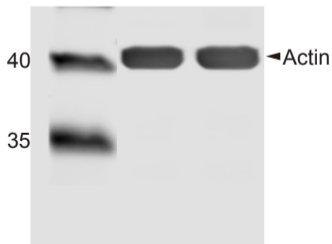

Supplement: Figure 1—source data 3. [file elife-91002-fig1-data3.zip › Figure 1 - Source data 3/Figure_1G_uncropped.pdf]

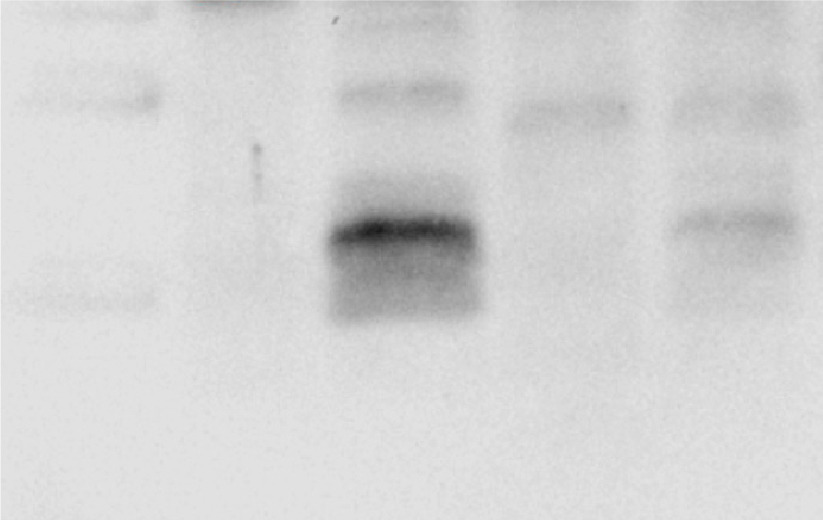

Supplement: Figure 1—source data 4. [file elife-91002-fig1-data4.zip › Figure 1 - Source data 4/Figure_1H- Source data_anti-Cleaved Caspase3_raw data.jpg]

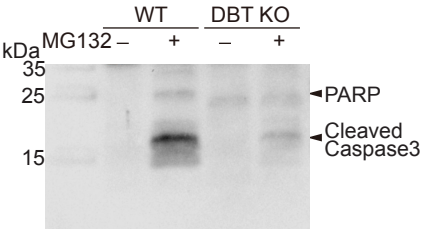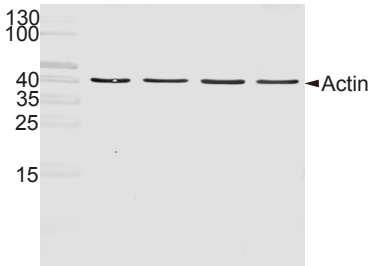

Supplement: Figure 1—source data 4. [file elife-91002-fig1-data4.zip › Figure 1 - Source data 4/Figure_1H_uncropped.pdf]

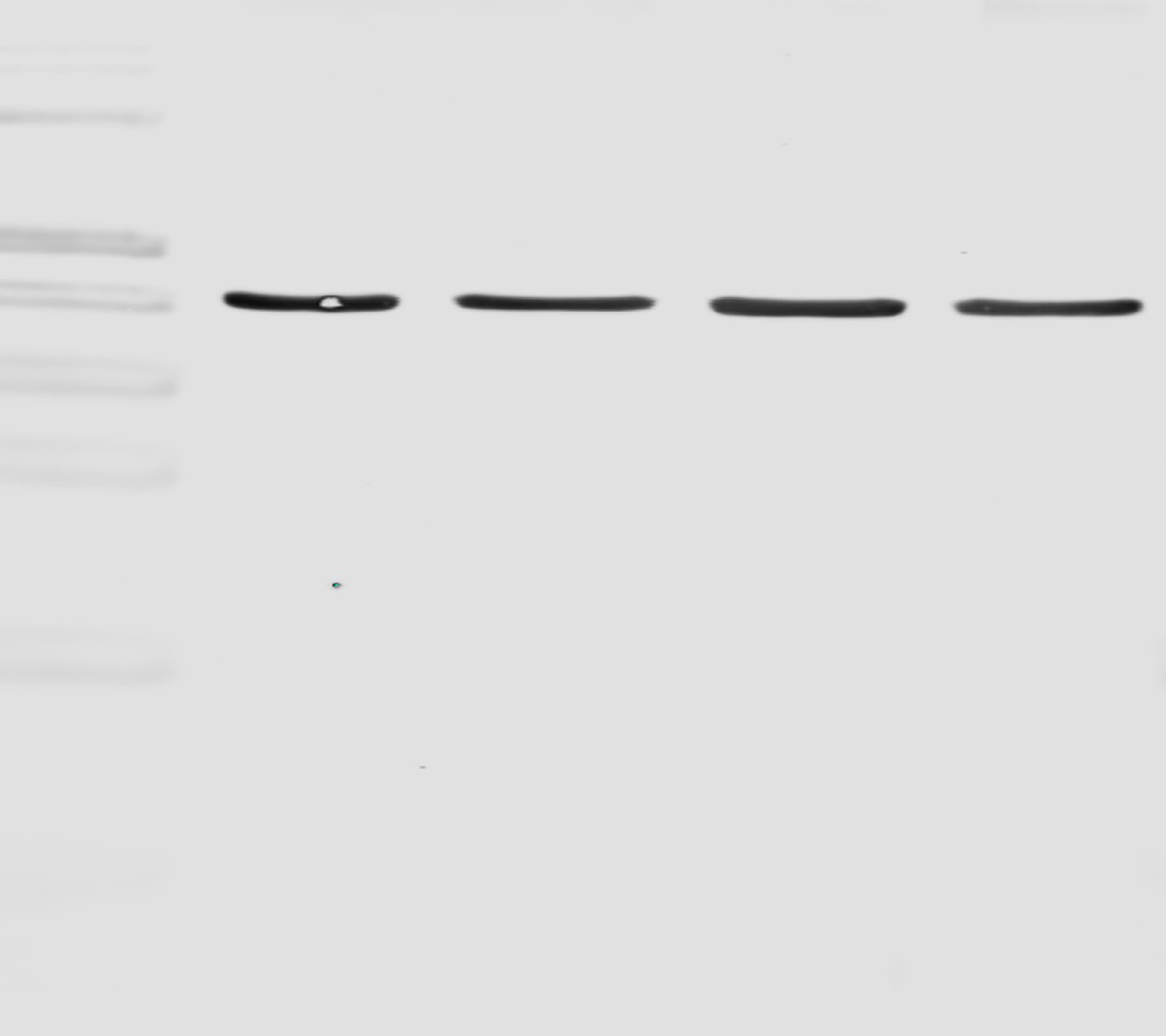

Supplement: Figure 1—source data 4. [file elife-91002-fig1-data4.zip › Figure 1 - Source data 4/Figure_1H- Source data_anti-Actin_raw data.jpg]

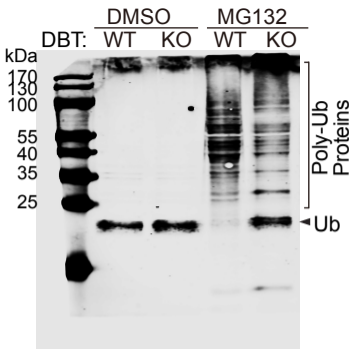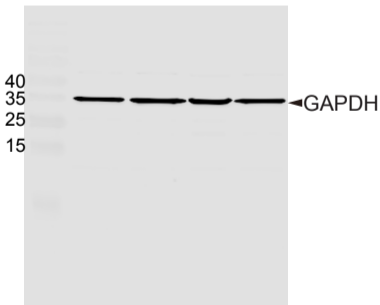

Supplement: Figure 2—source data 1. [file elife-91002-fig2-data1.zip › Figure 2 - Source data 1/Figure_2A_uncropped.pdf]

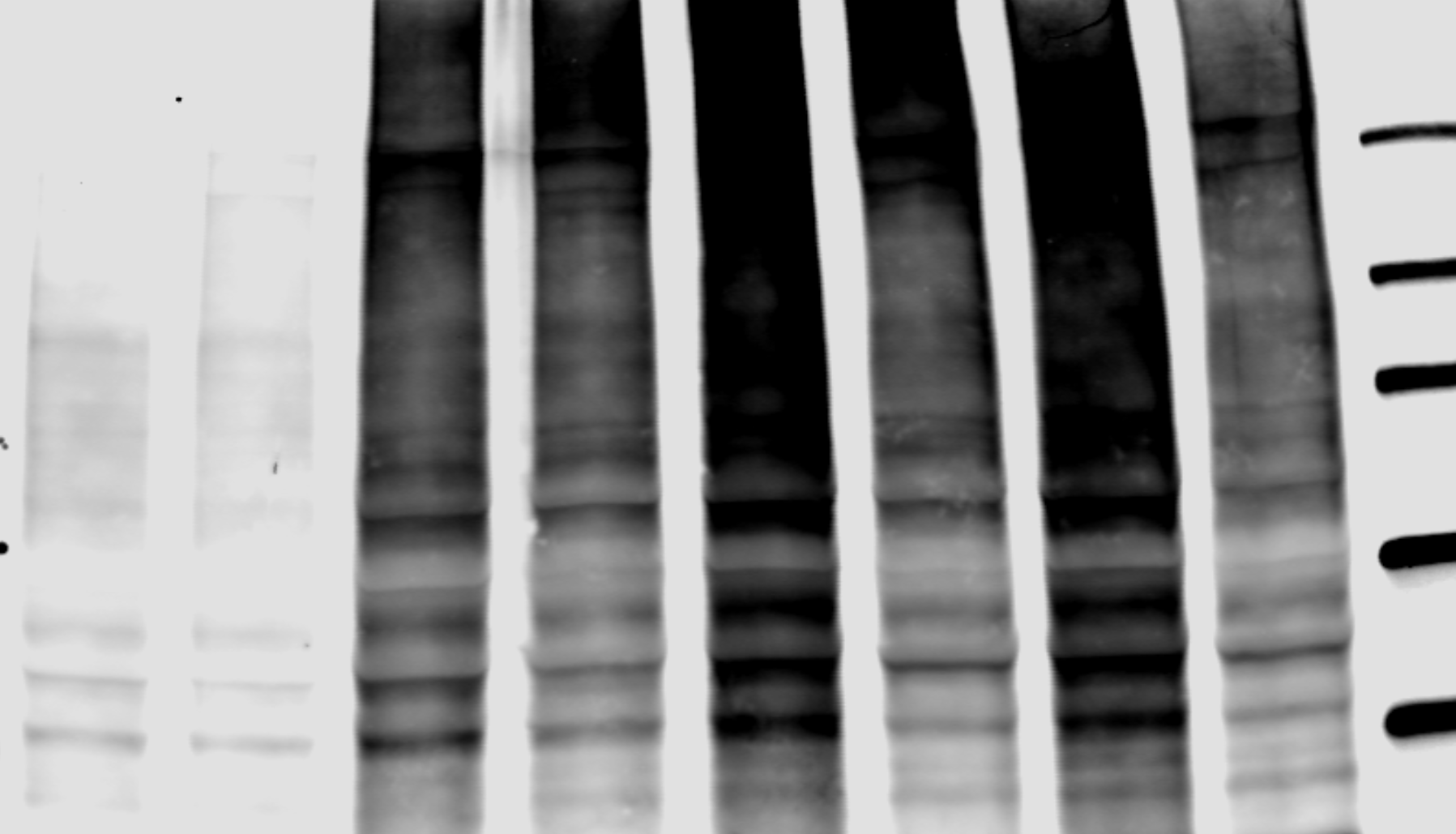

Supplement: Figure 2—figure supplement 1—source data 1. [file elife-91002-fig2-figsupp1-data1.zip › Figure 2 - Figure supplement1 - Source data 1/Figure 2 - Figure supplement1A - Source data_anti-Ubiquitin_raw data.tif]

# MG132

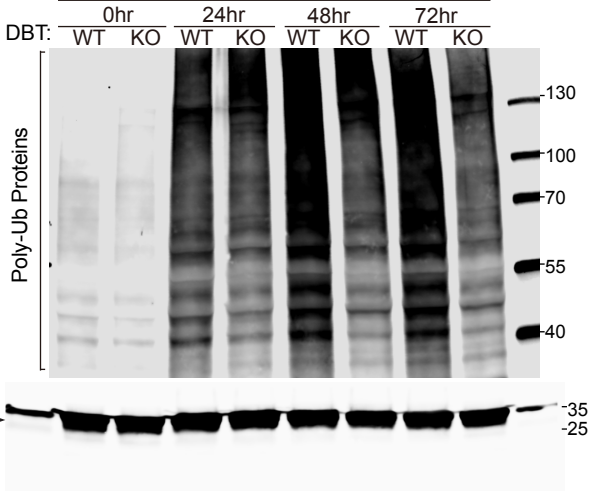

Supplement: Figure 2—figure supplement 1—source data 1. [file elife-91002-fig2-figsupp1-data1.zip › Figure 2 - Figure supplement1 - Source data 1/Figure 2 - Figure supplement1A_uncropped.pdf]

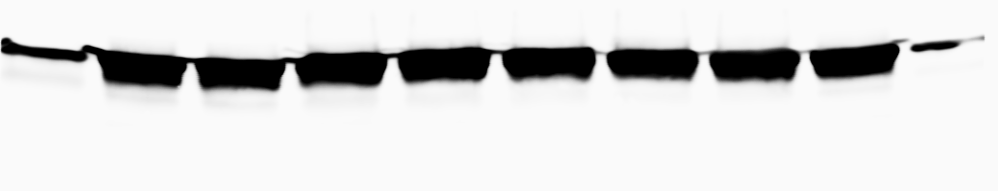

Supplement: Figure 2—figure supplement 1—source data 1. [file elife-91002-fig2-figsupp1-data1.zip › Figure 2 - Figure supplement1 - Source data 1/Figure 2 - Figure supplement1A - Source data_anti-GAPDH_raw data.tif]

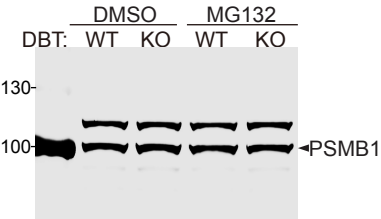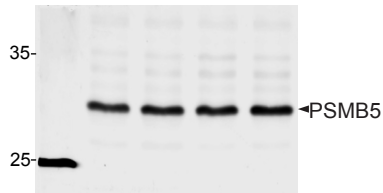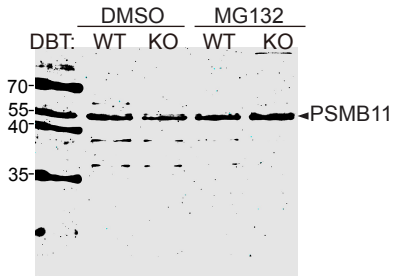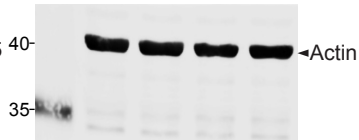

Supplement: Figure 2—figure supplement 1—source data 2. [file elife-91002-fig2-figsupp1-data2.zip › Figure 2 - Figure supplement1 - Source data 2/Figure 2 - Figure supplement1C_uncropped.pdf]

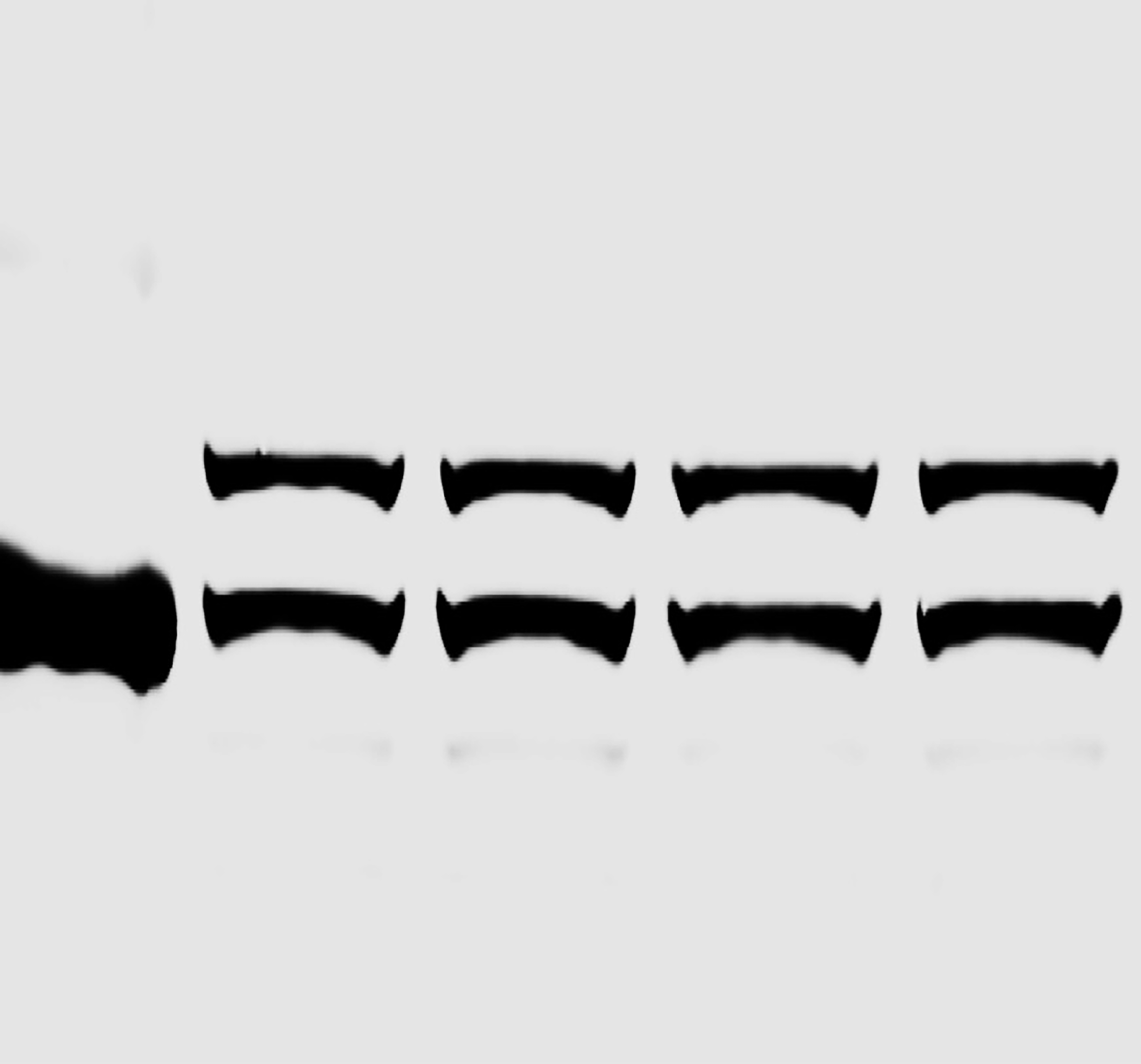

Supplement: Figure 2—figure supplement 1—source data 2. [file elife-91002-fig2-figsupp1-data2.zip › Figure 2 - Figure supplement1 - Source data 2/Figure 2 - Figure supplement1C - Source data_anti-PSMD1_raw data.jpg]

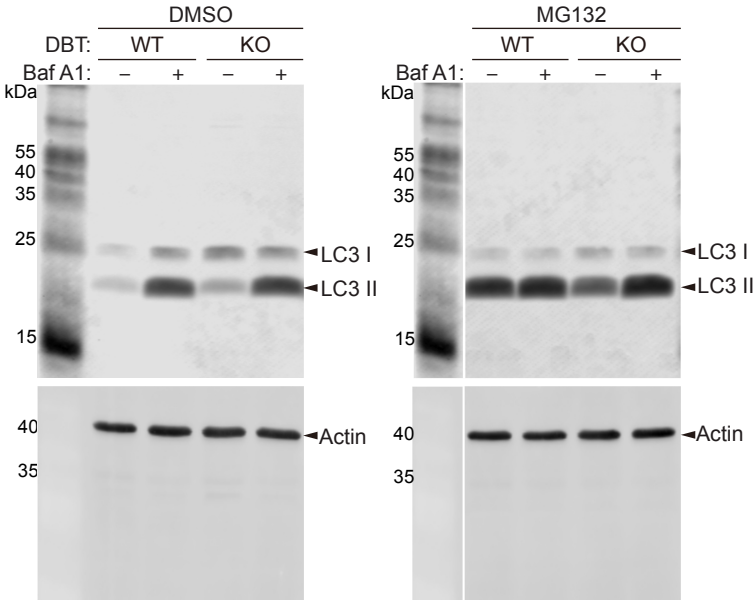

Supplement: Figure 3—source data 1. [file elife-91002-fig3-data1.zip › Figure 3 - Source data 1/Figure_3A_uncropped.pdf]

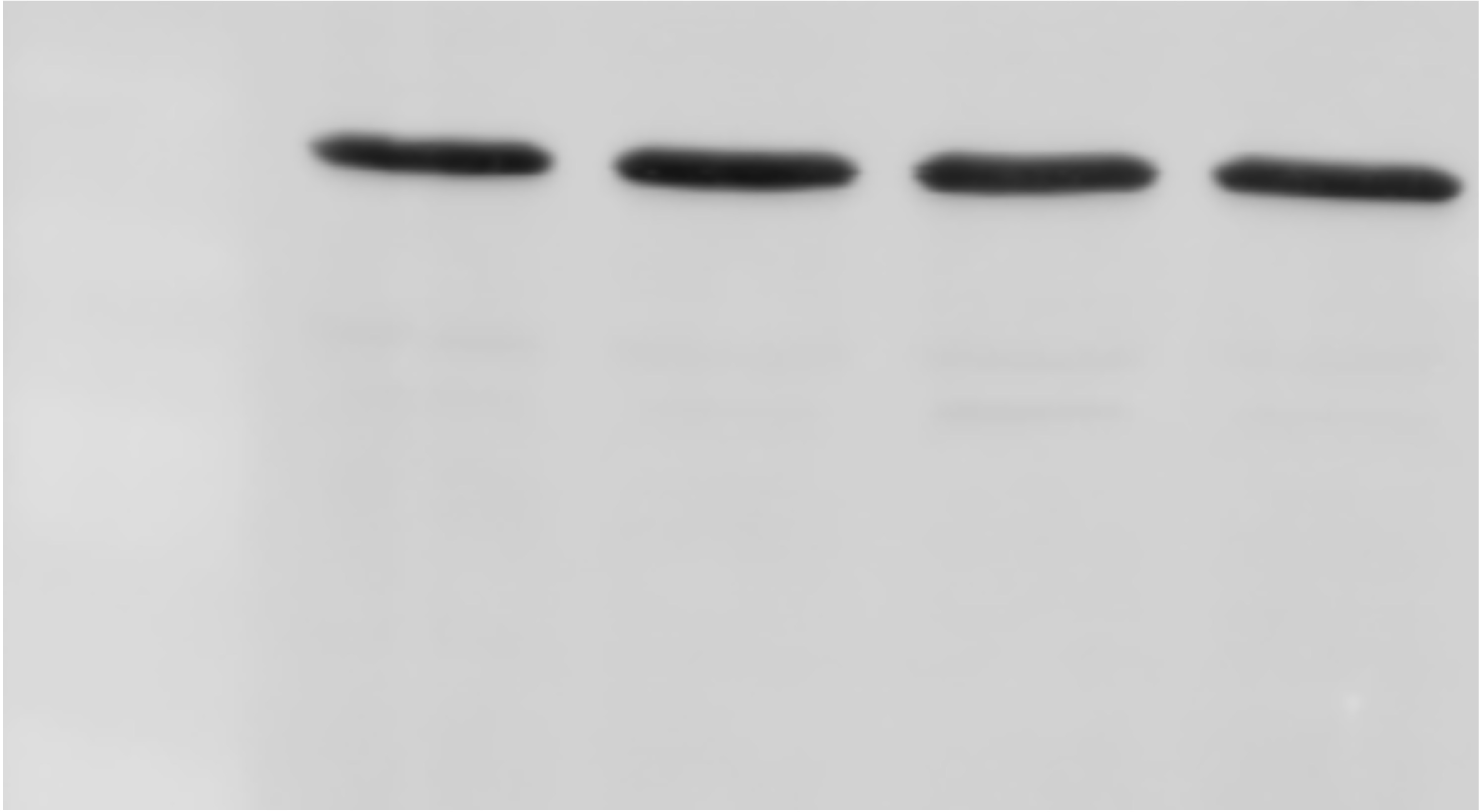

Supplement: Figure 3—source data 1. [file elife-91002-fig3-data1.zip › Figure 3 - Source data 1/Figure_3A- Source data_anti-Actin_raw data.jpg]

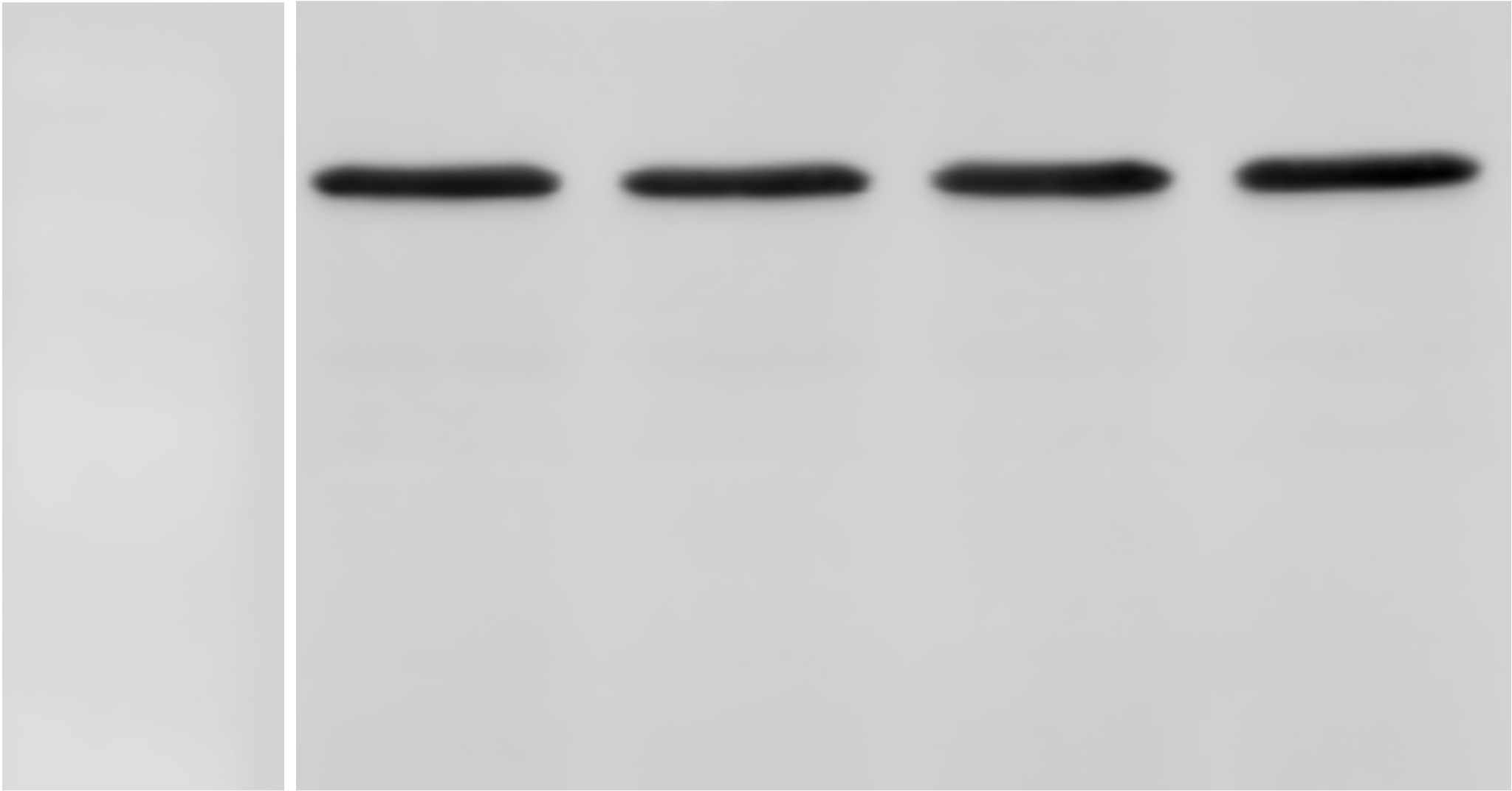

Supplement: Figure 3—source data 1. [file elife-91002-fig3-data1.zip › Figure 3 - Source data 1/Figure_3A- Source data_anti-GAPDH_raw data2.jpg]

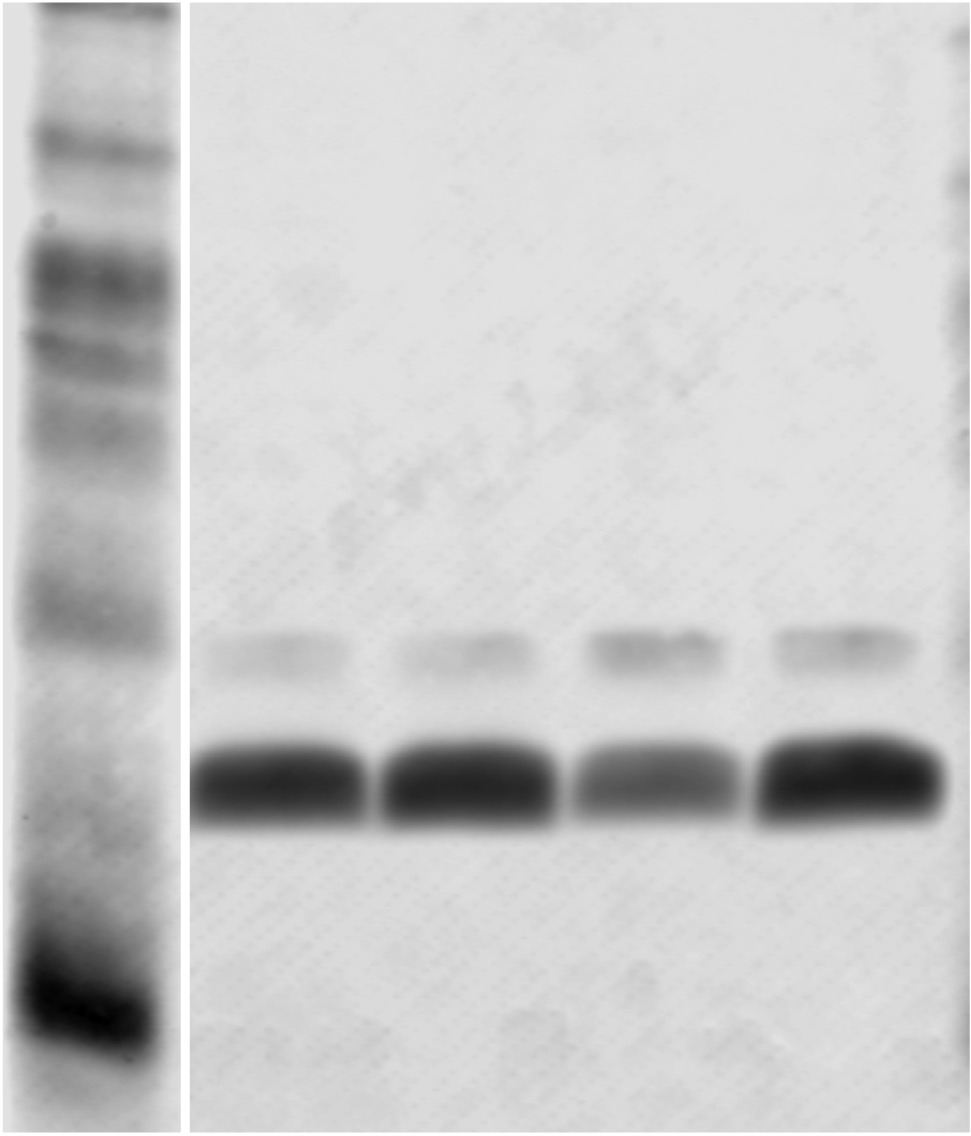

Supplement: Figure 3—source data 1. [file elife-91002-fig3-data1.zip › Figure 3 - Source data 1/Figure_3A- Source data_anti-LC3_raw data2.jpg]

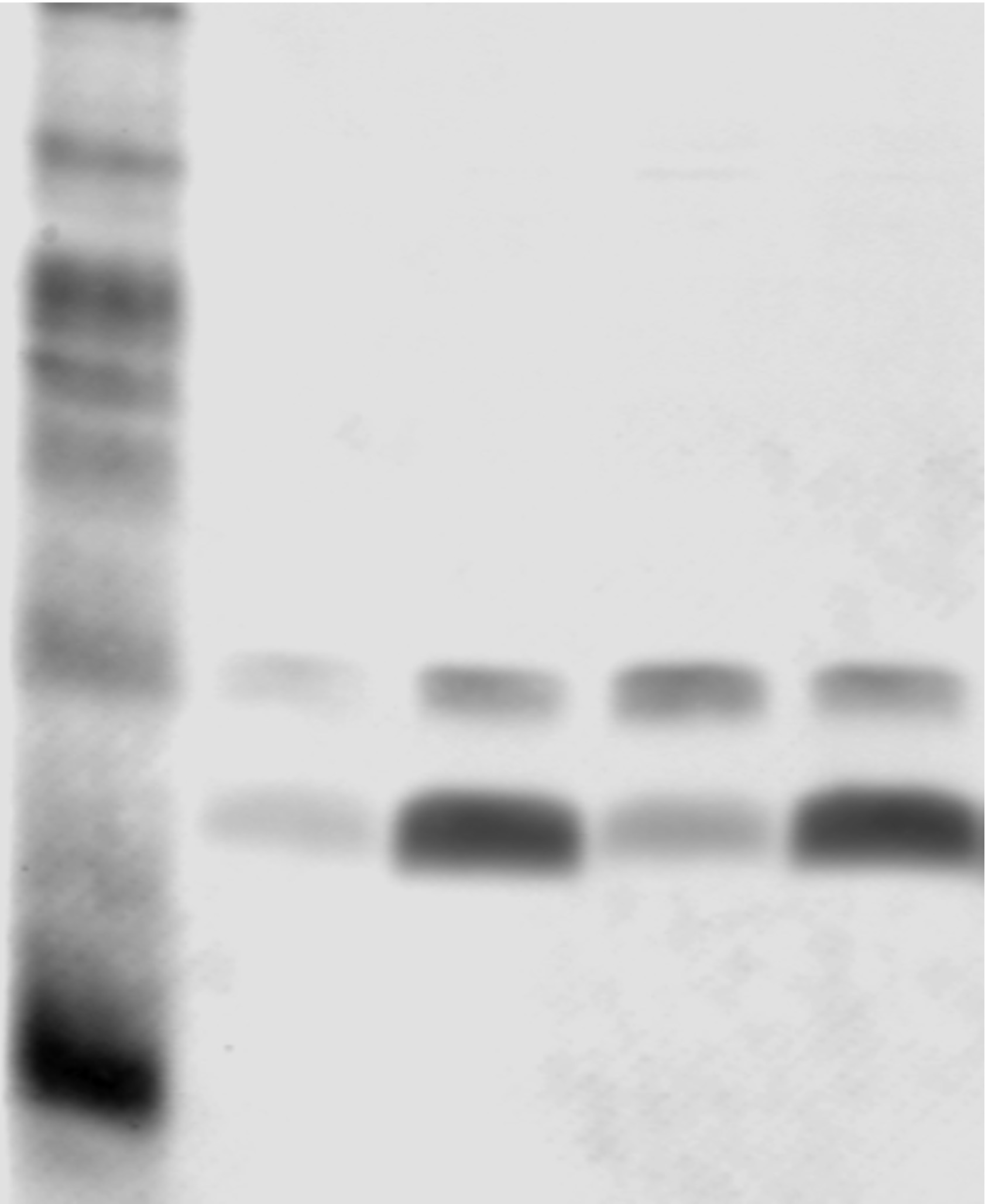

Supplement: Figure 3—source data 1. [file elife-91002-fig3-data1.zip › Figure 3 - Source data 1/Figure_3A- Source data_anti-LC3_raw data.jpg]

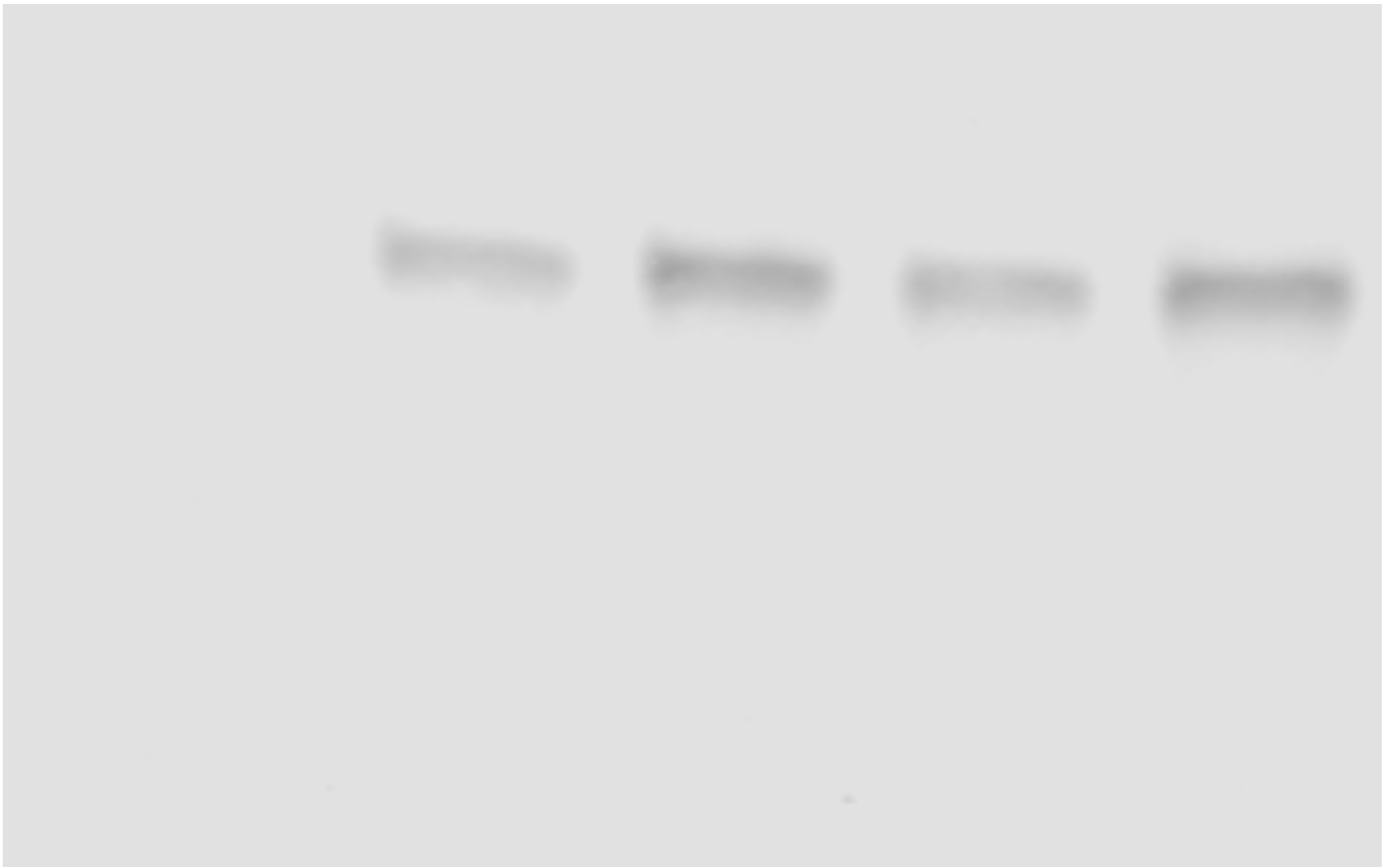

Supplement: Figure 3—source data 2. [file elife-91002-fig3-data2.zip › Figure 3 - Source data 2/Figure_3D- Source data_anti-P62_raw data.jpg]

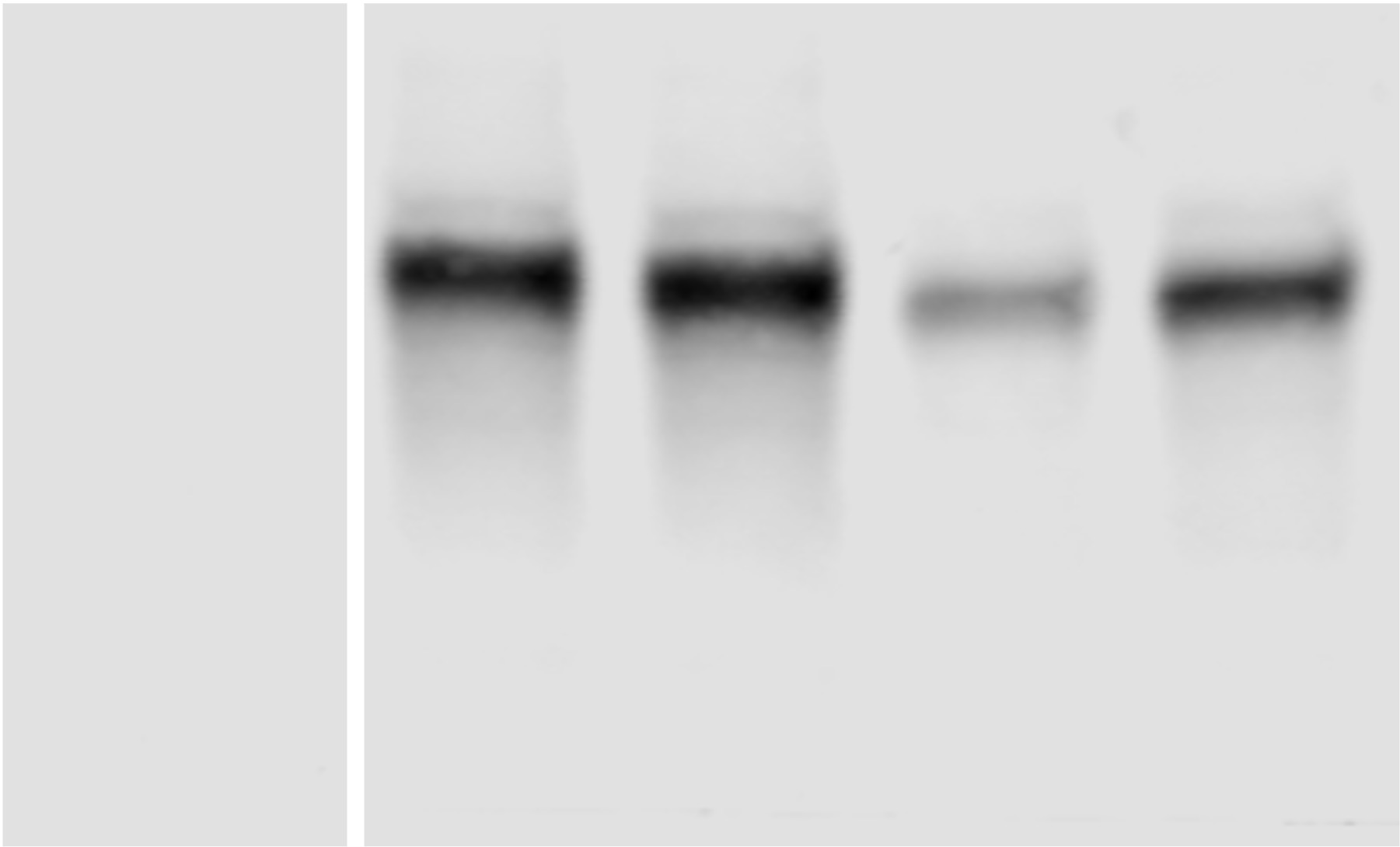

Supplement: Figure 3—source data 2. [file elife-91002-fig3-data2.zip › Figure 3 - Source data 2/Figure_3D- Source data_anti-P62_raw data2.jpg]

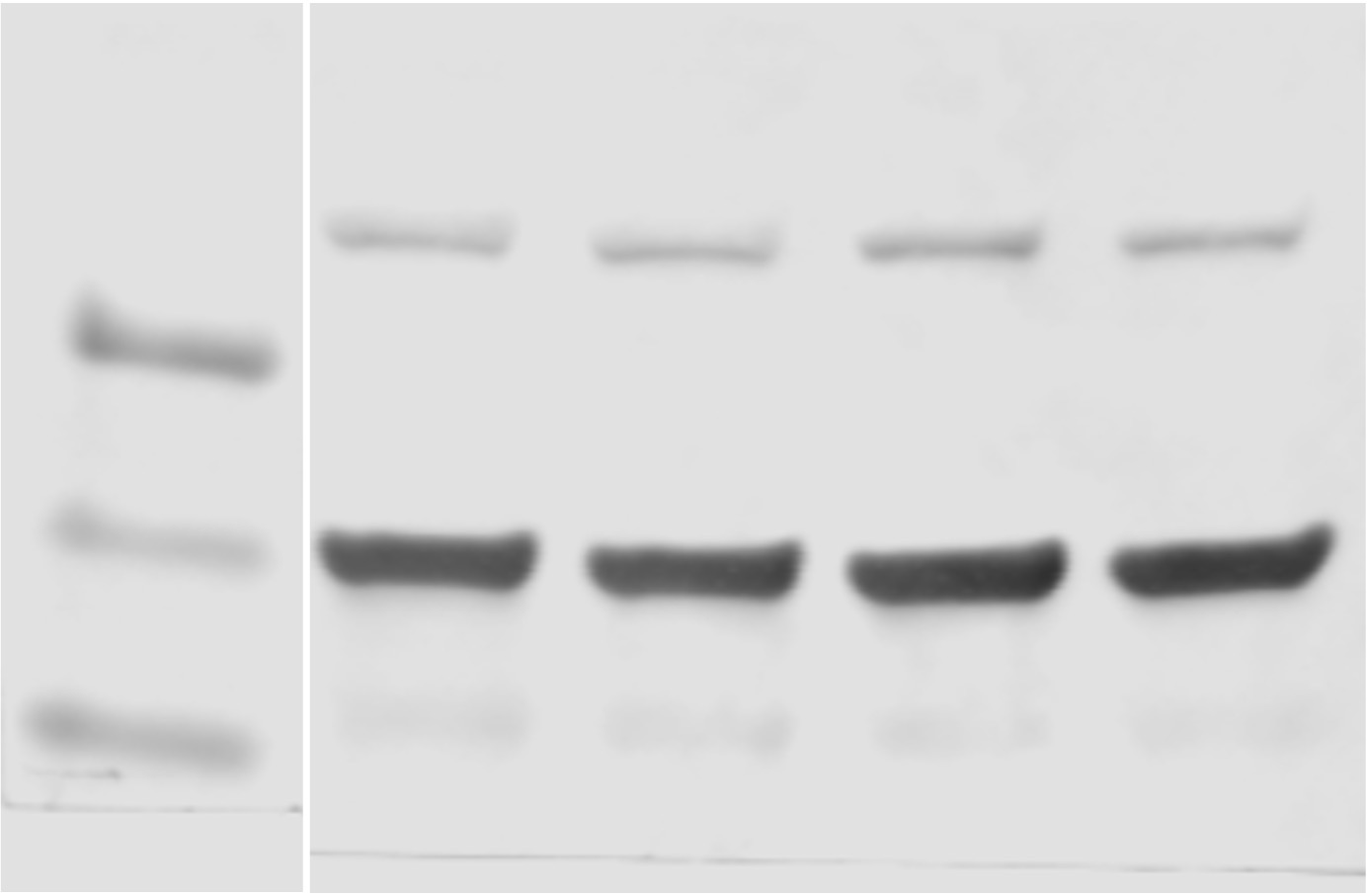

Supplement: Figure 3—source data 2. [file elife-91002-fig3-data2.zip › Figure 3 - Source data 2/Figure_3D- Source data_anti-Actin_raw data2.jpg]

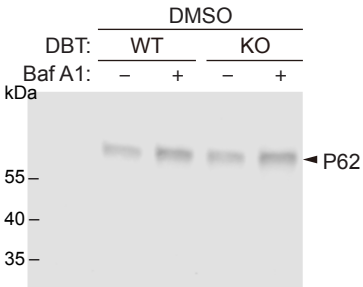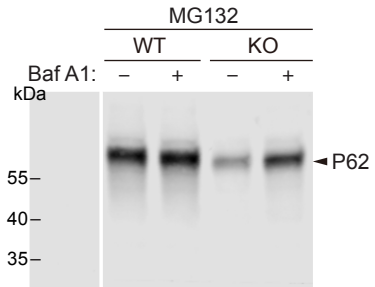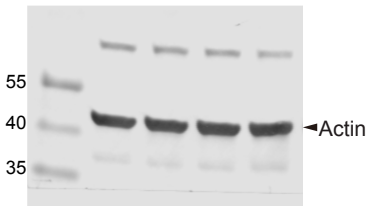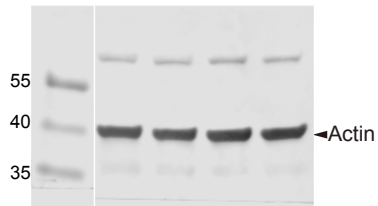

Supplement: Figure 3—source data 2. [file elife-91002-fig3-data2.zip › Figure 3 - Source data 2/Figure_3D_uncropped.pdf]

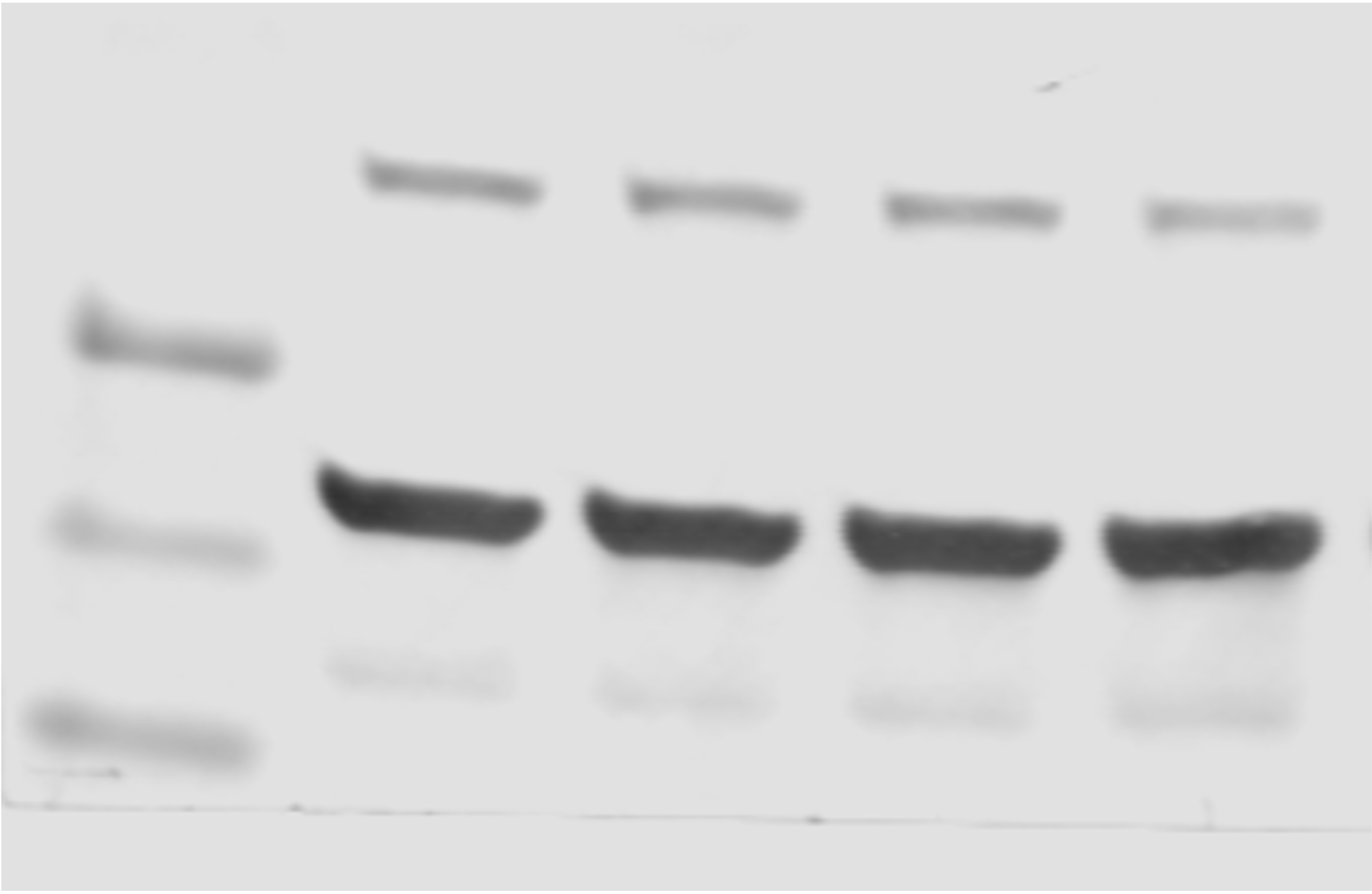

Supplement: Figure 3—source data 2. [file elife-91002-fig3-data2.zip › Figure 3 - Source data 2/Figure_3D- Source data_anti-Actin_raw data.jpg]

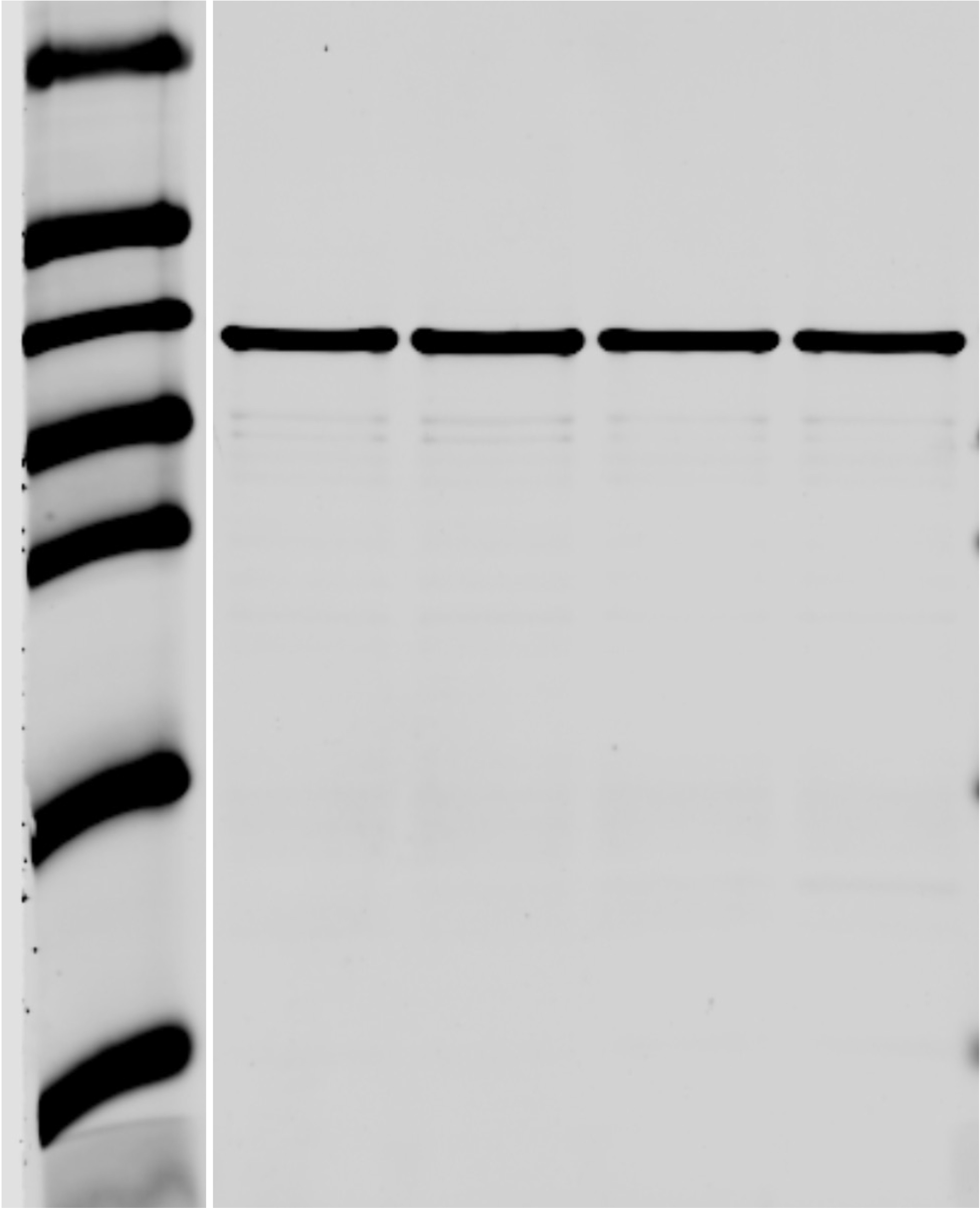

Supplement: Figure 3—figure supplement 1—source data 1. [file elife-91002-fig3-figsupp1-data1.zip › Figure 3 - Figure supplement1 - Source data 1/Figure 3 - Figure supplement1A - Source data_anti-Actin_raw data.jpg]

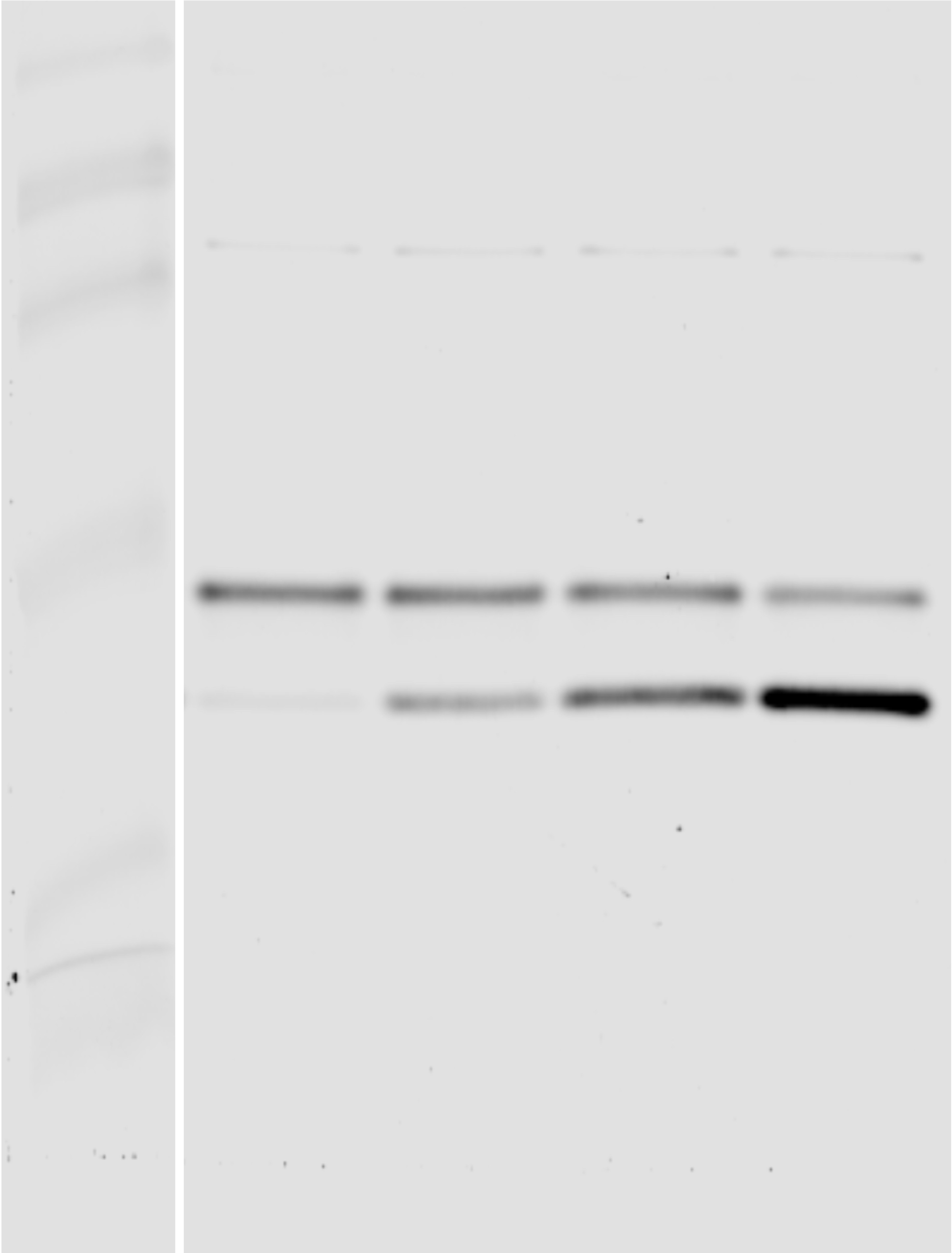

Supplement: Figure 3—figure supplement 1—source data 1. [file elife-91002-fig3-figsupp1-data1.zip › Figure 3 - Figure supplement1 - Source data 1/Figure 3 - Figure supplement1A - Source data_anti-LC3_raw data.jpg]

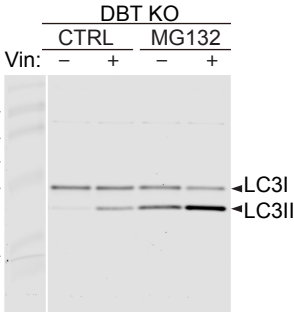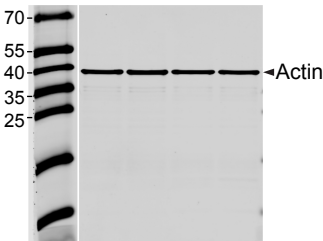

Supplement: Figure 3—figure supplement 1—source data 1. [file elife-91002-fig3-figsupp1-data1.zip › Figure 3 - Figure supplement1 - Source data 1/Figure 3 - Figure supplement1A_uncropped.pdf]

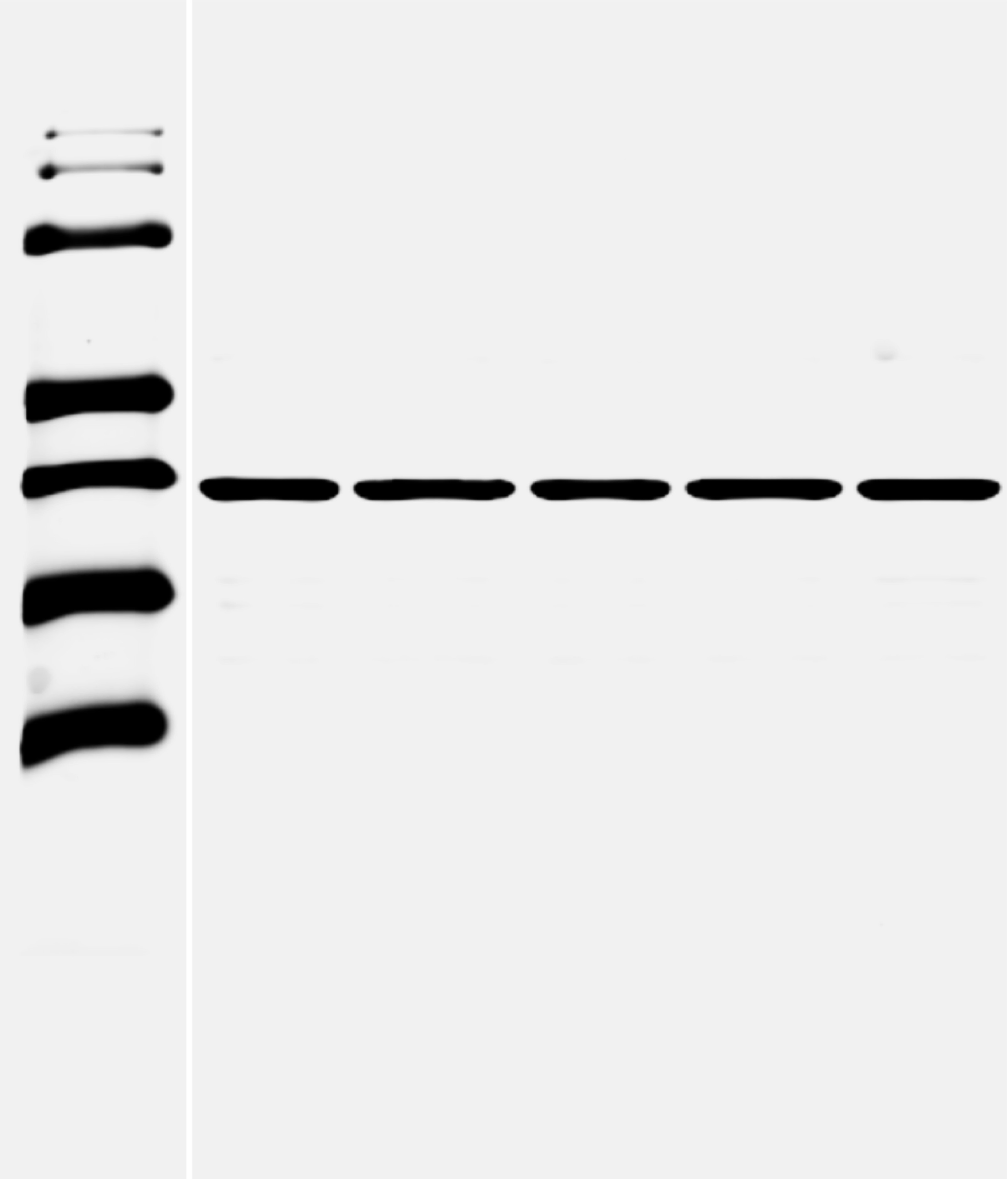

Supplement: Figure 3—figure supplement 1—source data 2. [file elife-91002-fig3-figsupp1-data2.zip › Figure 3 - Figure supplement1 - Source data 2/Figure 3 - Figure supplement1B - Source data_anti-Actin_raw data2.jpg]

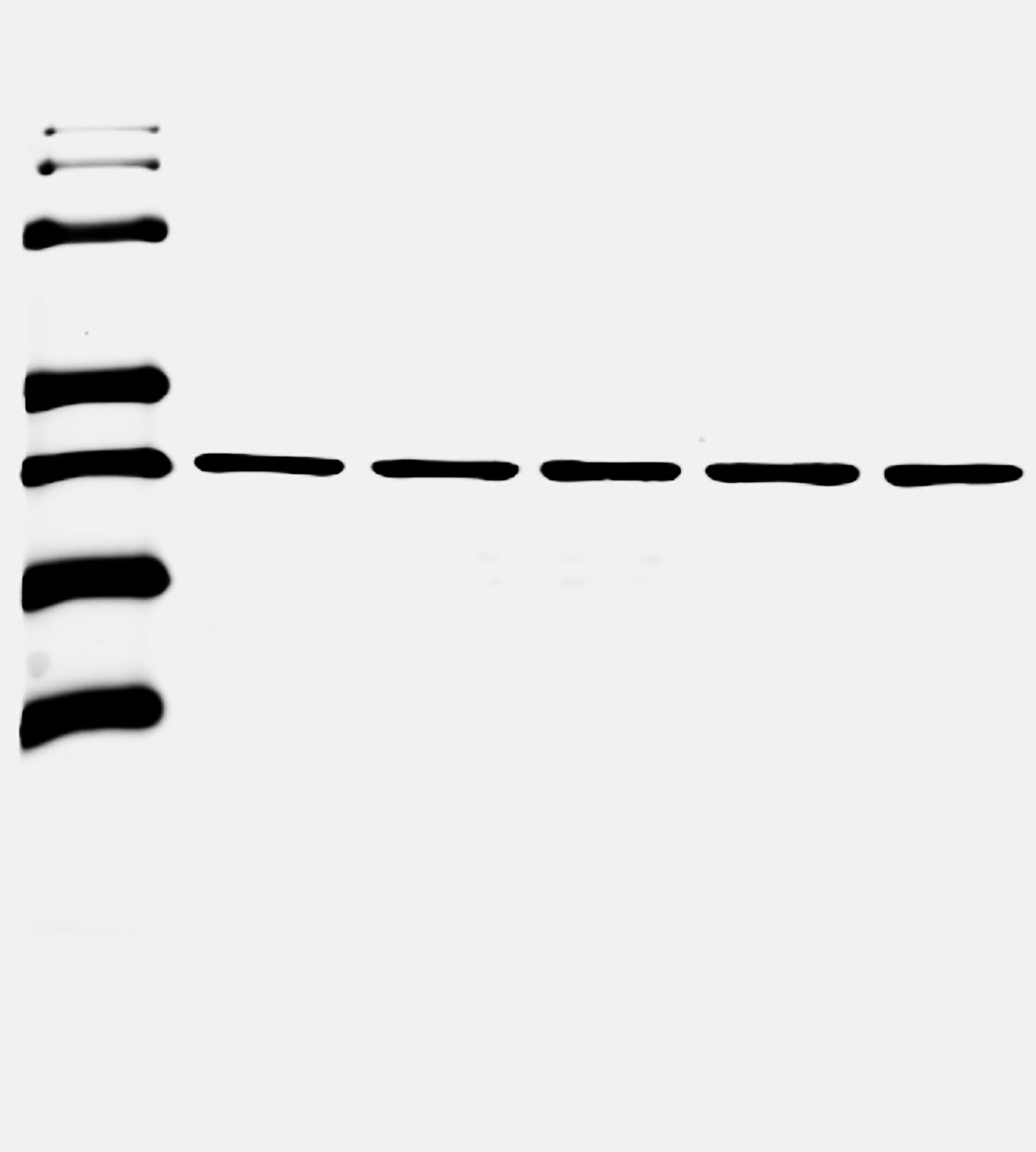

Supplement: Figure 3—figure supplement 1—source data 2. [file elife-91002-fig3-figsupp1-data2.zip › Figure 3 - Figure supplement1 - Source data 2/Figure 3 - Figure supplement1B - Source data_anti-Actin_raw data.jpg]

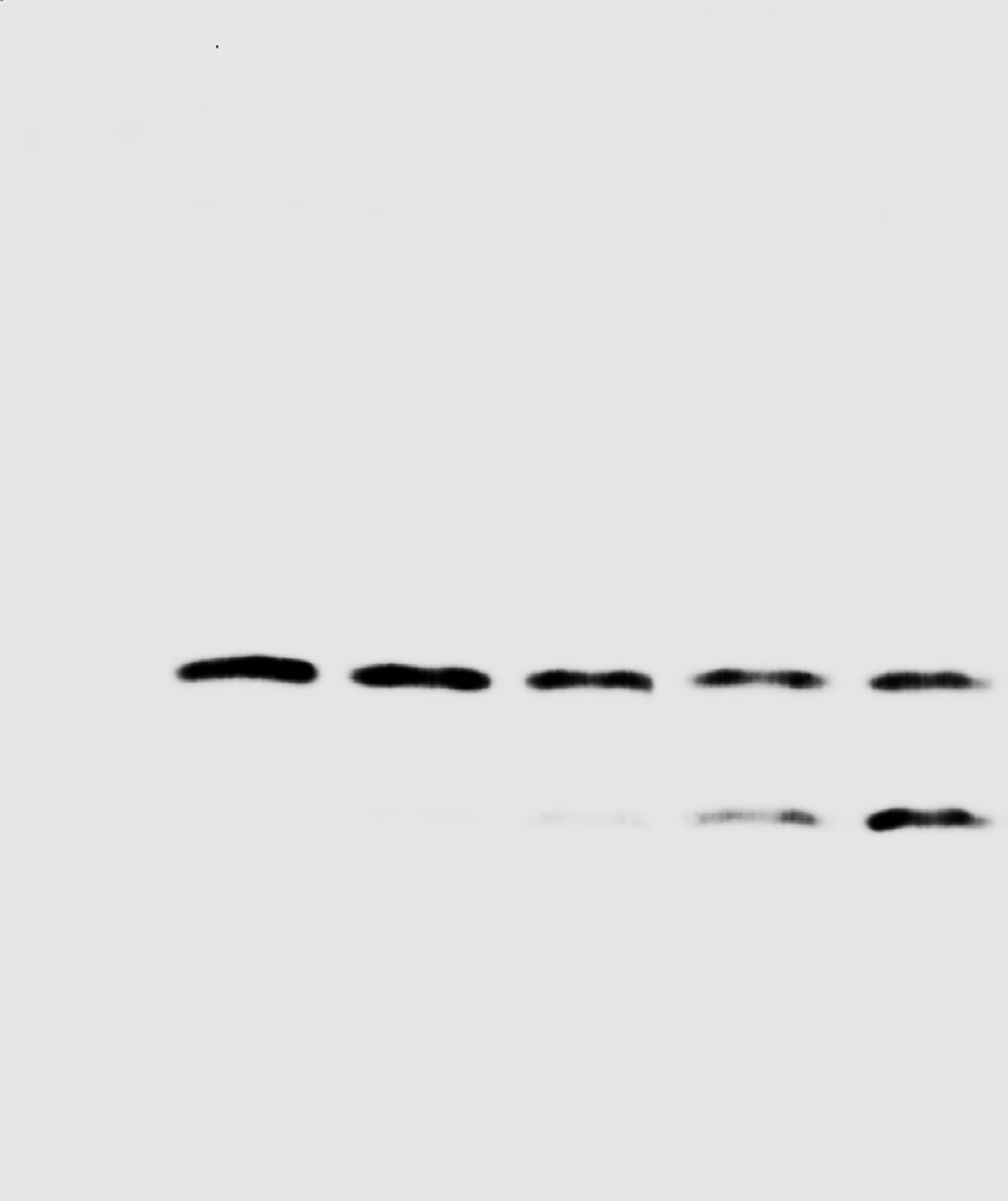

Supplement: Figure 3—figure supplement 1—source data 2. [file elife-91002-fig3-figsupp1-data2.zip › Figure 3 - Figure supplement1 - Source data 2/Figure 3 - Figure supplement1B - Source data_anti-LC3_raw data.jpg]

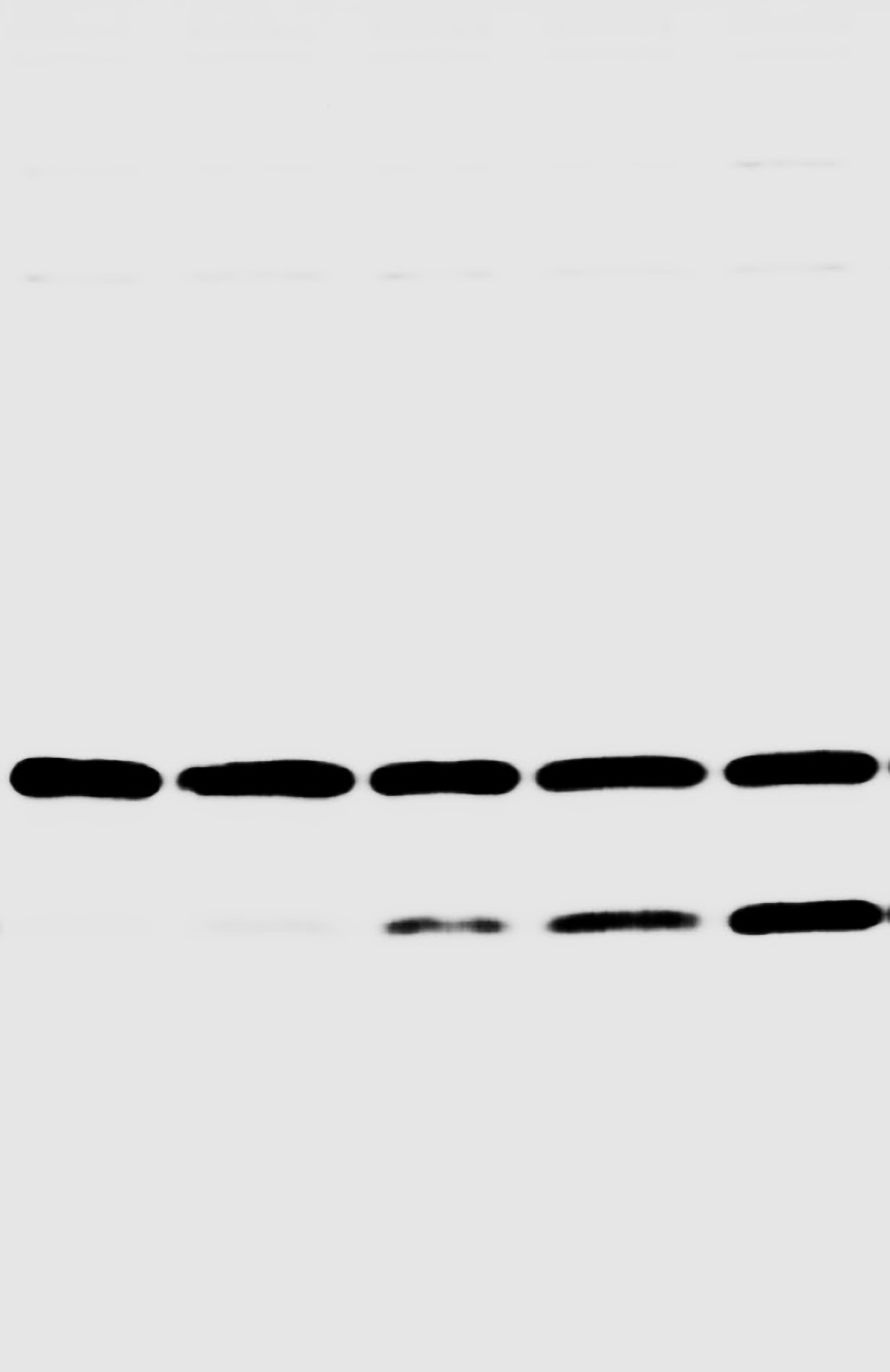

Supplement: Figure 3—figure supplement 1—source data 2. [file elife-91002-fig3-figsupp1-data2.zip › Figure 3 - Figure supplement1 - Source data 2/Figure 3 - Figure supplement1B - Source data_anti-LC3_raw data2.jpg]

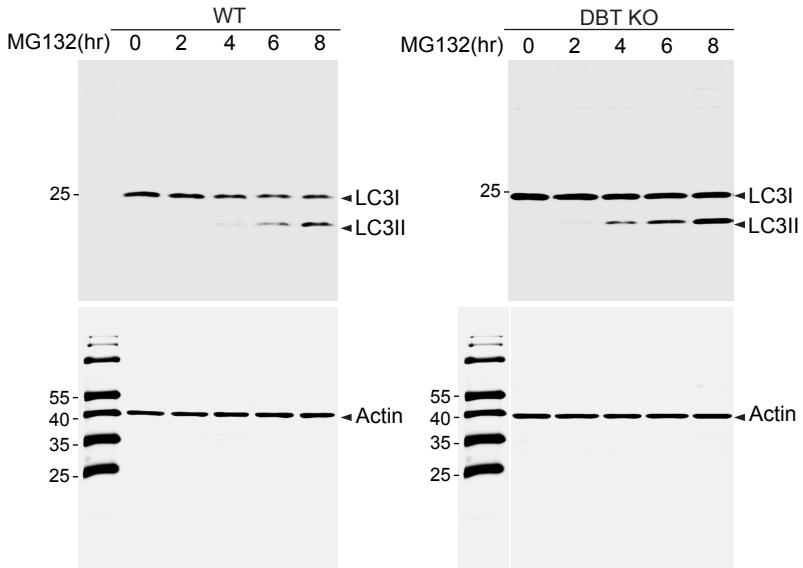

Supplement: Figure 3—figure supplement 1—source data 2. [file elife-91002-fig3-figsupp1-data2.zip › Figure 3 - Figure supplement1 - Source data 2/Figure 3 - Figure supplement1B_uncropped.pdf]

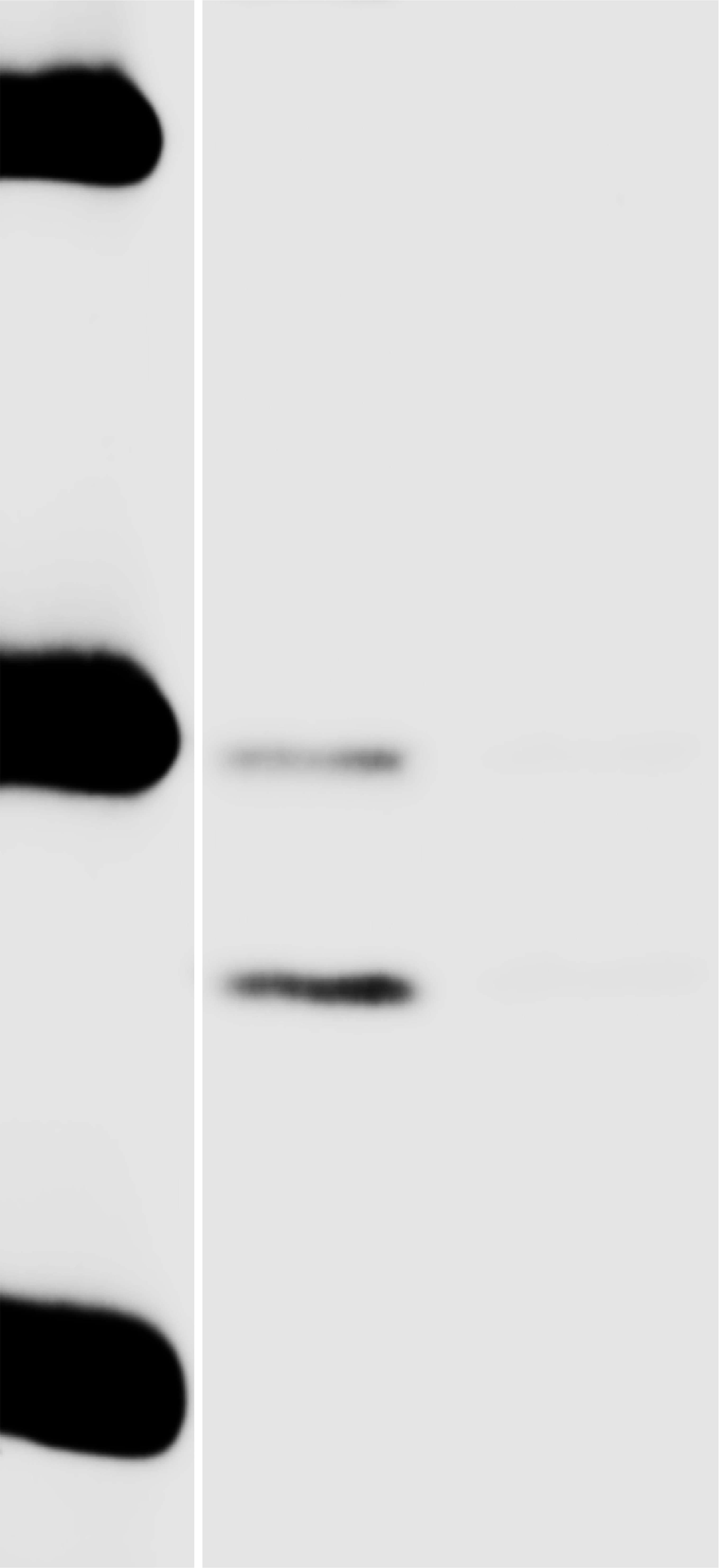

Supplement: Figure 3—figure supplement 1—source data 3. [file elife-91002-fig3-figsupp1-data3.zip › Figure 3 - Figure supplement1 - Source data 3/Figure 3 - Figure supplement1D - Source data_anti-LC3_raw data.jpg]

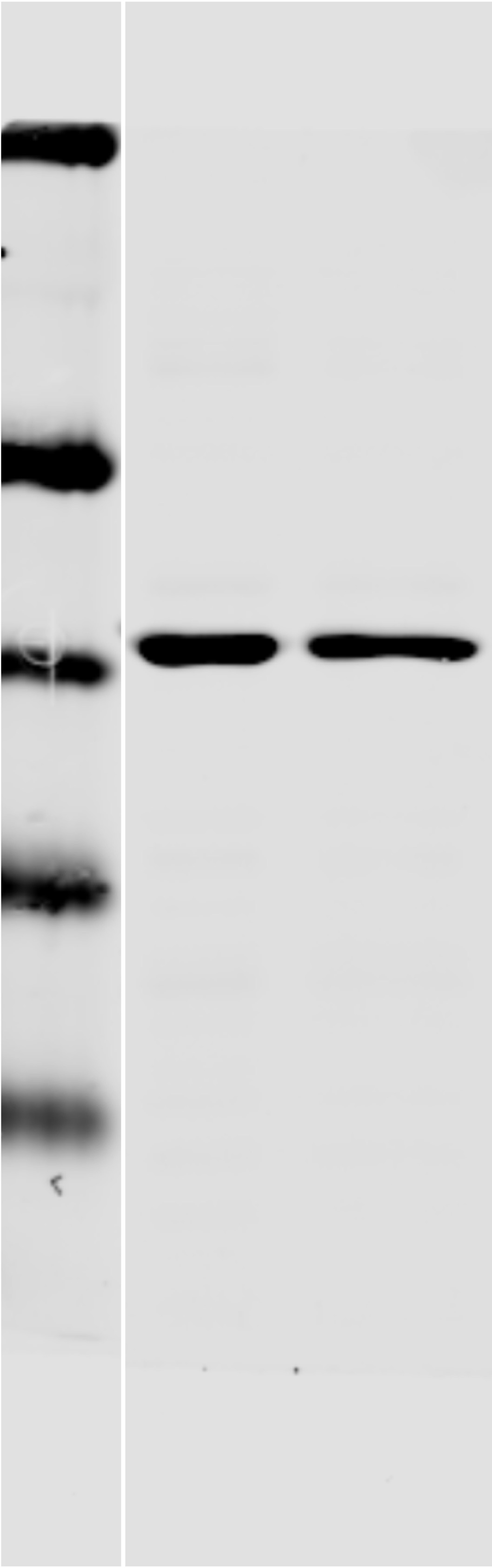

Supplement: Figure 3—figure supplement 1—source data 3. [file elife-91002-fig3-figsupp1-data3.zip › Figure 3 - Figure supplement1 - Source data 3/Figure 3 - Figure supplement1D - Source data_anti-Actin_raw data.jpg]

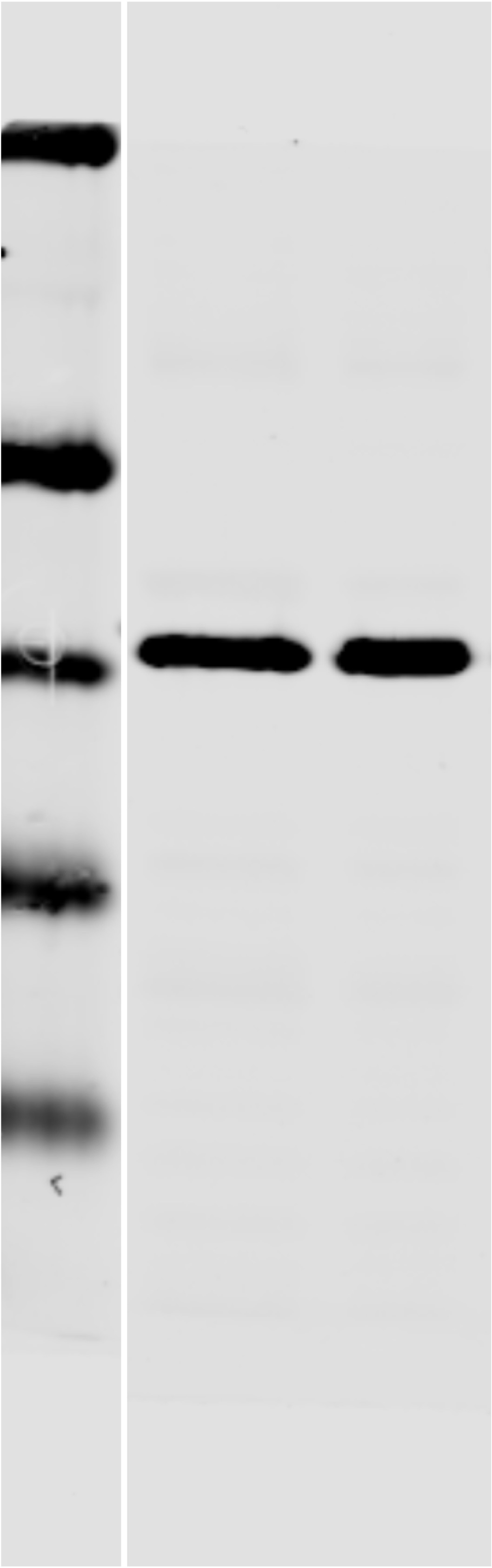

Supplement: Figure 3—figure supplement 1—source data 3. [file elife-91002-fig3-figsupp1-data3.zip › Figure 3 - Figure supplement1 - Source data 3/Figure 3 - Figure supplement1D - Source data_anti-Actin_raw data2.jpg]

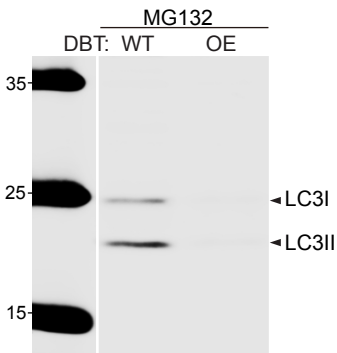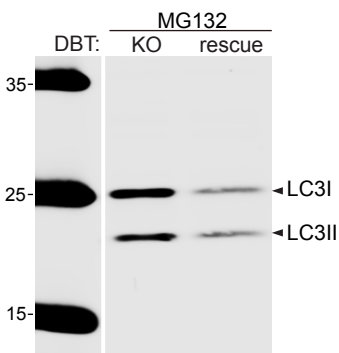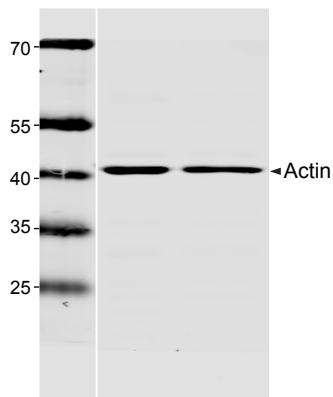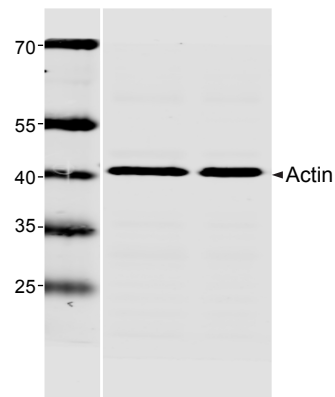

Supplement: Figure 3—figure supplement 1—source data 3. [file elife-91002-fig3-figsupp1-data3.zip › Figure 3 - Figure supplement1 - Source data 3/Figure 3 - Figure supplement1D_uncropped.pdf]

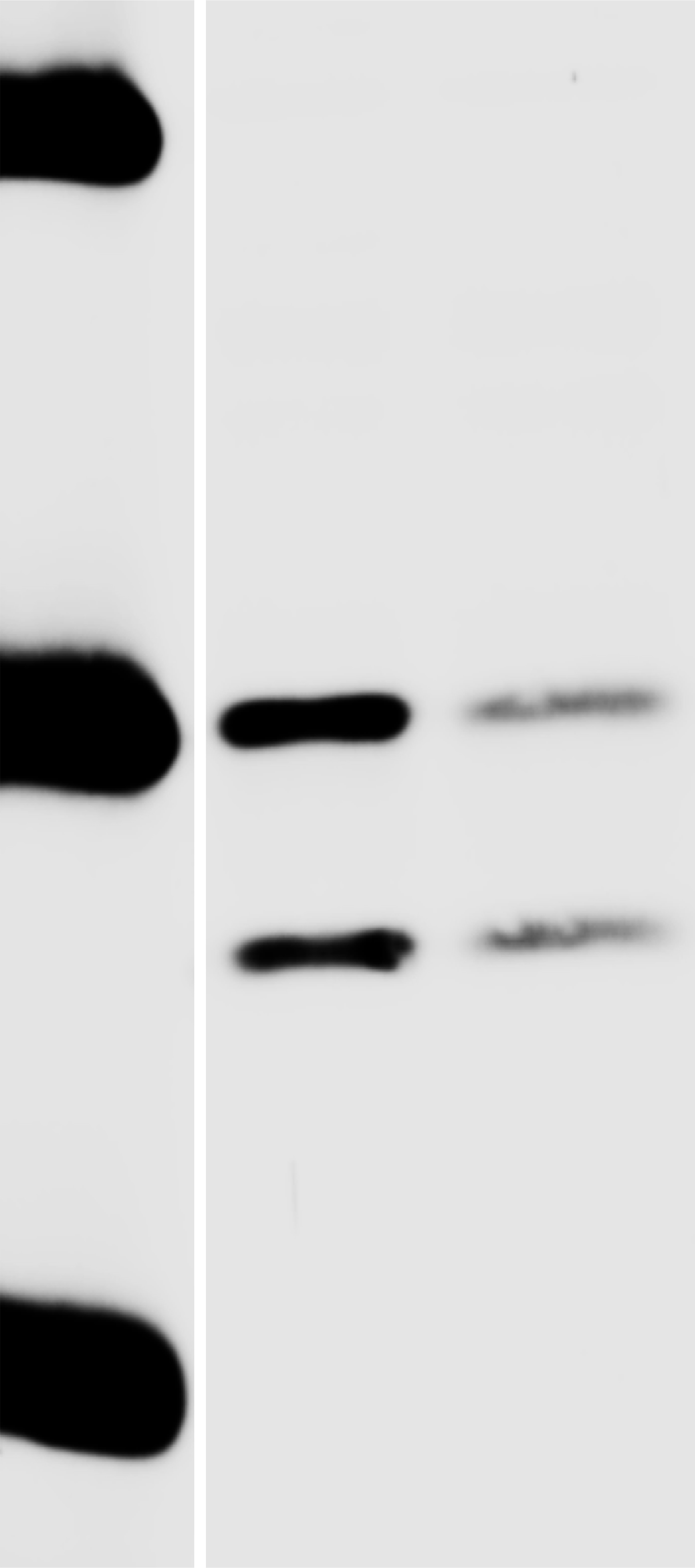

Supplement: Figure 3—figure supplement 1—source data 3. [file elife-91002-fig3-figsupp1-data3.zip › Figure 3 - Figure supplement1 - Source data 3/Figure 3 - Figure supplement1D - Source data_anti-LC3_raw data2.jpg]

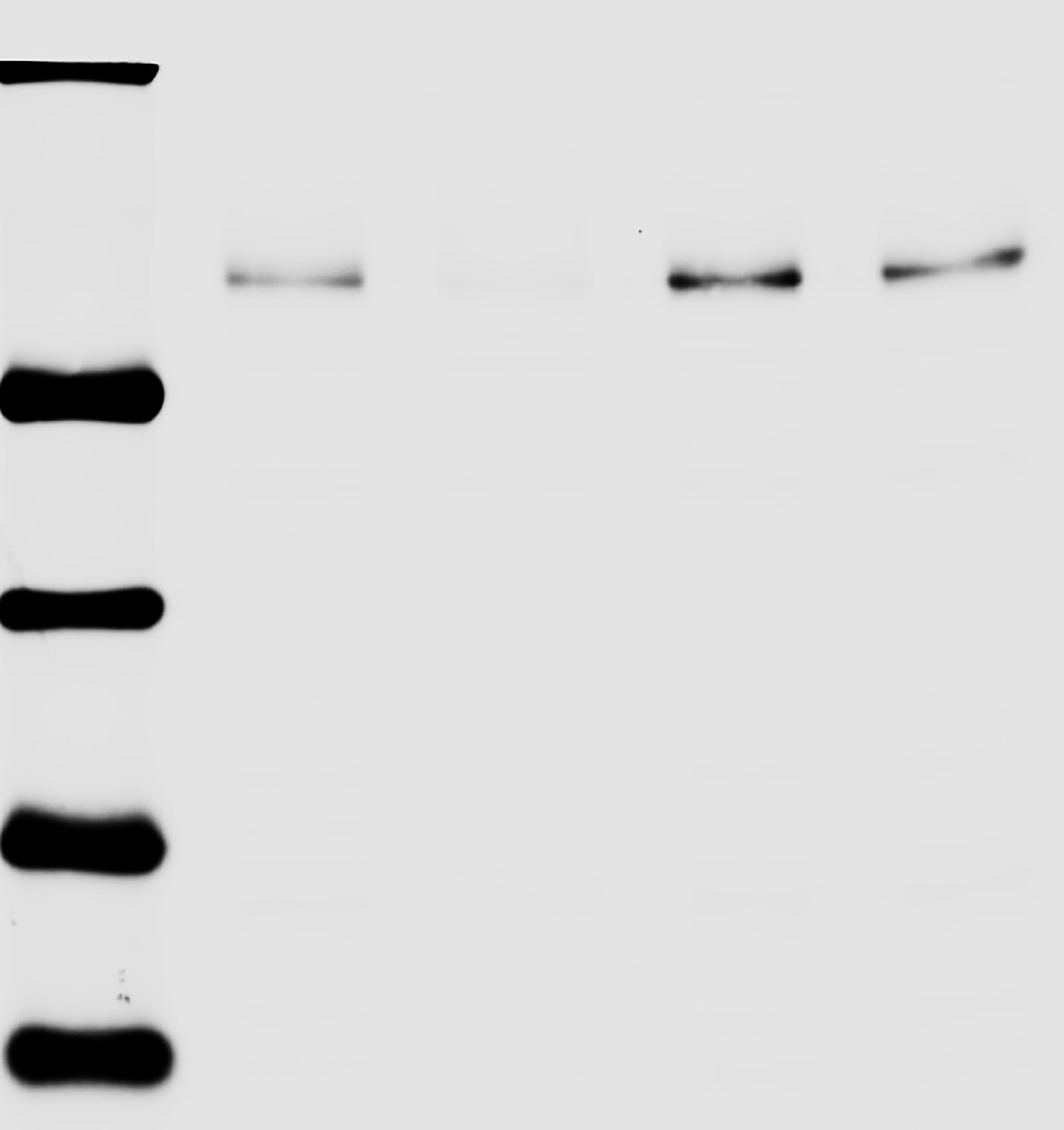

Supplement: Figure 3—figure supplement 1—source data 4. [file elife-91002-fig3-figsupp1-data4.zip › Figure 3 - Figure supplement1 - Source data 4/Figure 3 - Figure supplement1E - Source data_anti-pAMPK_raw data.jpg]

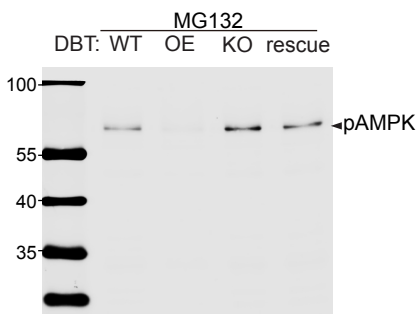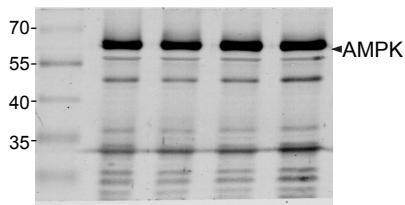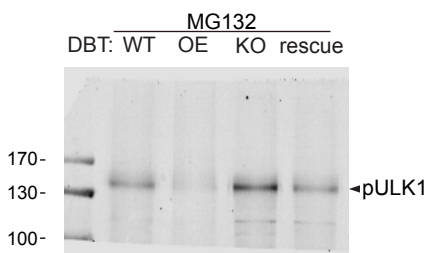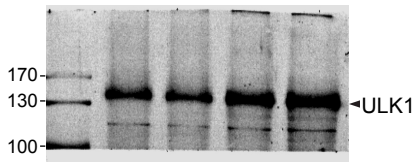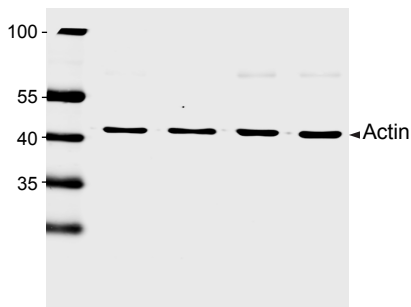

Supplement: Figure 3—figure supplement 1—source data 4. [file elife-91002-fig3-figsupp1-data4.zip › Figure 3 - Figure supplement1 - Source data 4/Figure 3 - Figure supplement1E_uncropped.pdf]

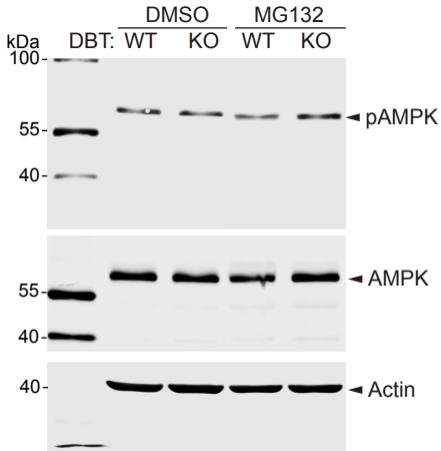

Supplement: Figure 4—source data 1. [file elife-91002-fig4-data1.zip › Figure 4 - Source data 1/Figure_4C_uncropped.pdf]

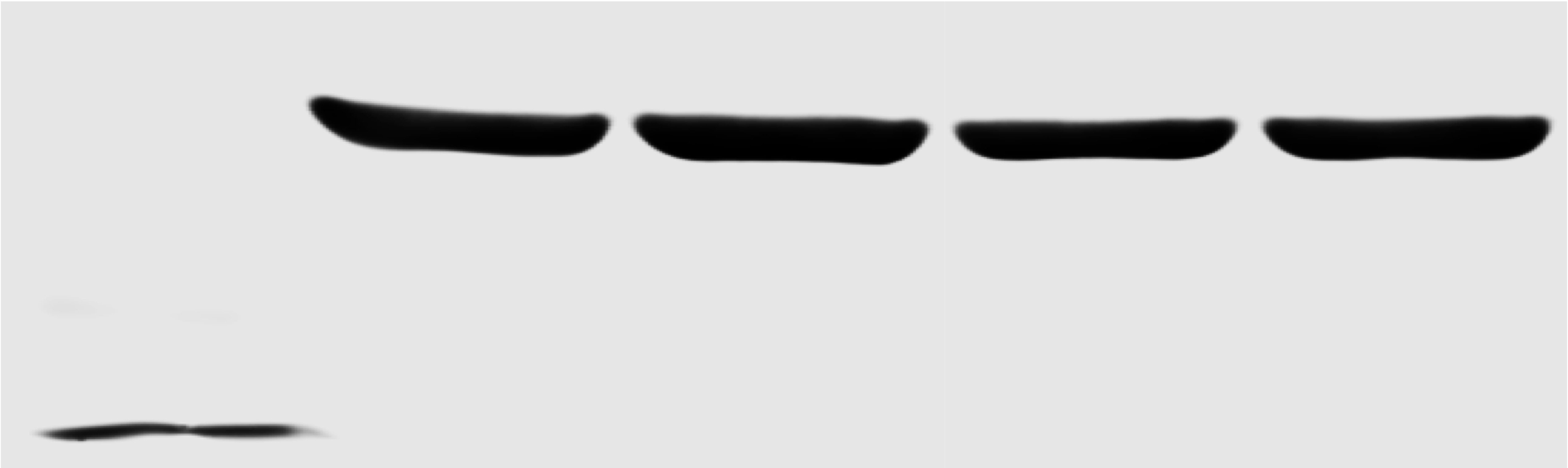

Supplement: Figure 4—source data 1. [file elife-91002-fig4-data1.zip › Figure 4 - Source data 1/Figure_4C- Source data_anti-Actin_raw data.jpg]

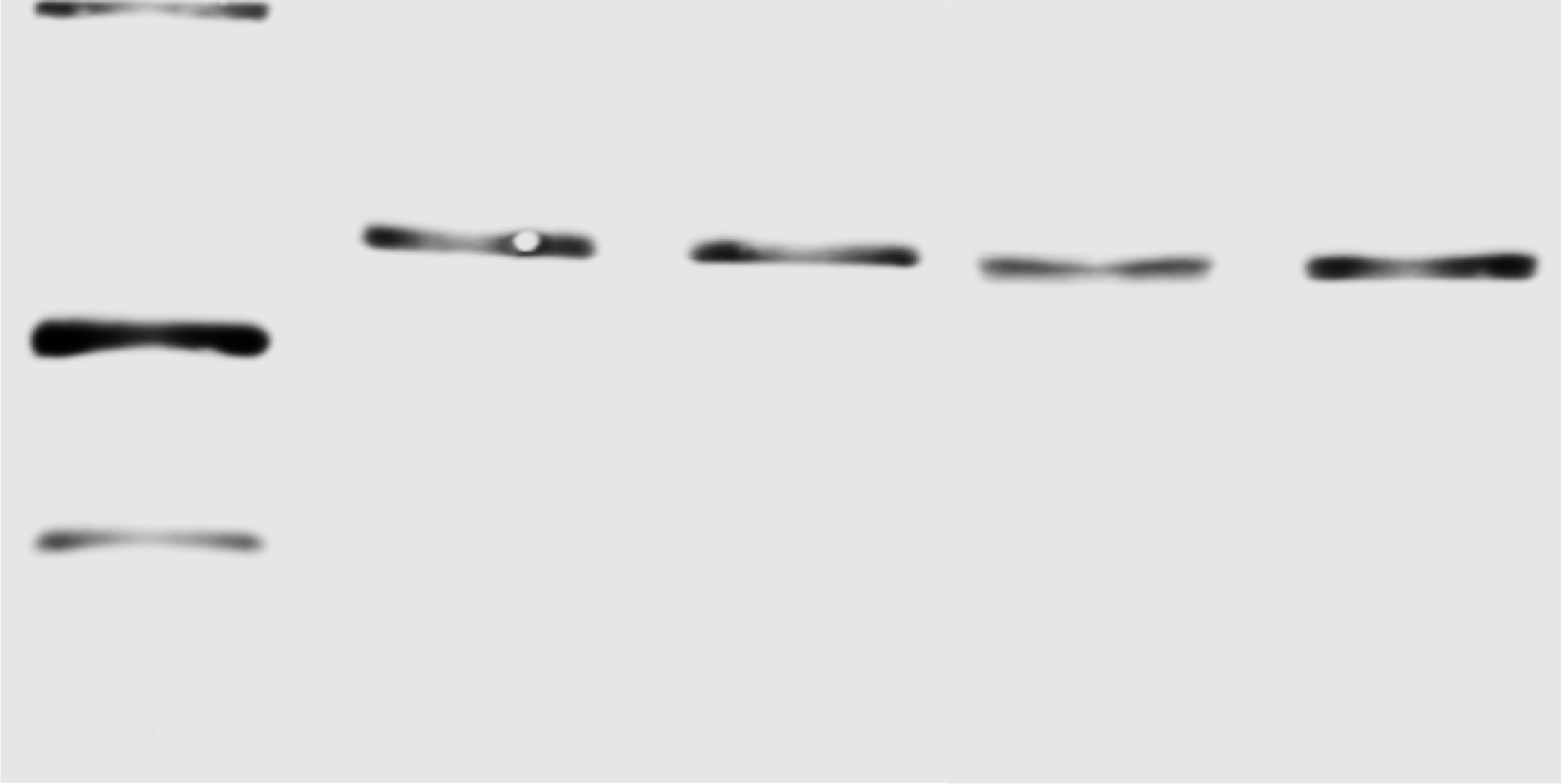

Supplement: Figure 4—source data 1. [file elife-91002-fig4-data1.zip › Figure 4 - Source data 1/Figure_4C- Source data_anti-pAMPK_raw data.jpg]

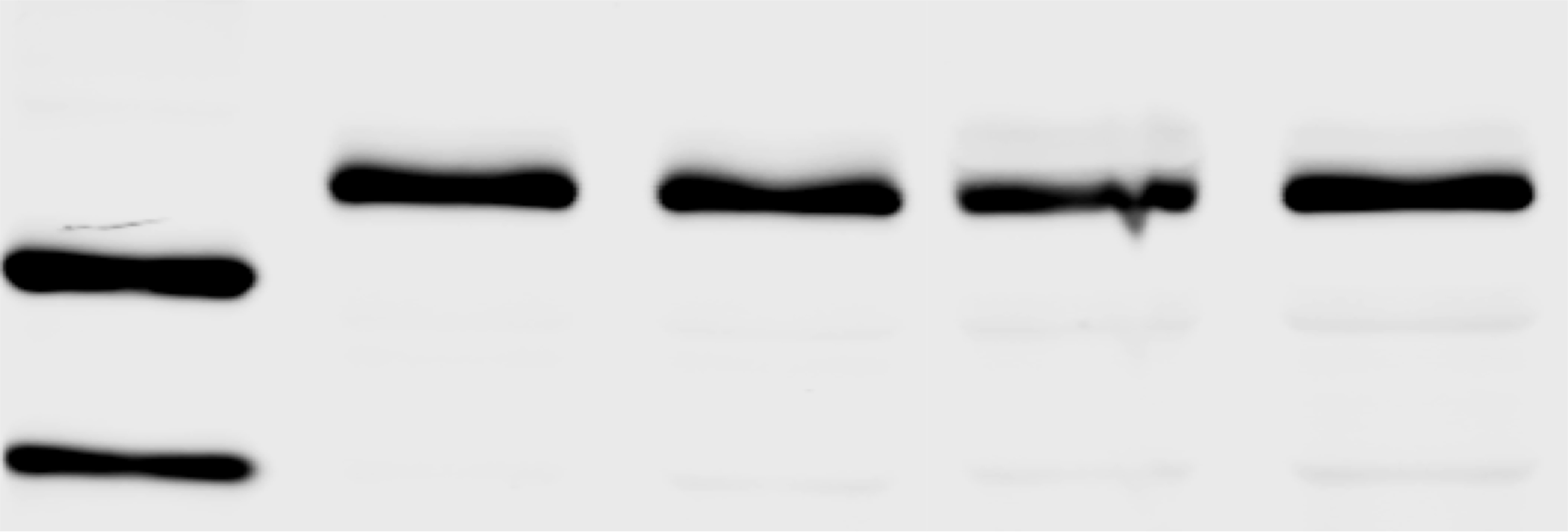

Supplement: Figure 4—source data 1. [file elife-91002-fig4-data1.zip › Figure 4 - Source data 1/Figure_4C- Source data_anti-AMPK_raw data.jpg]

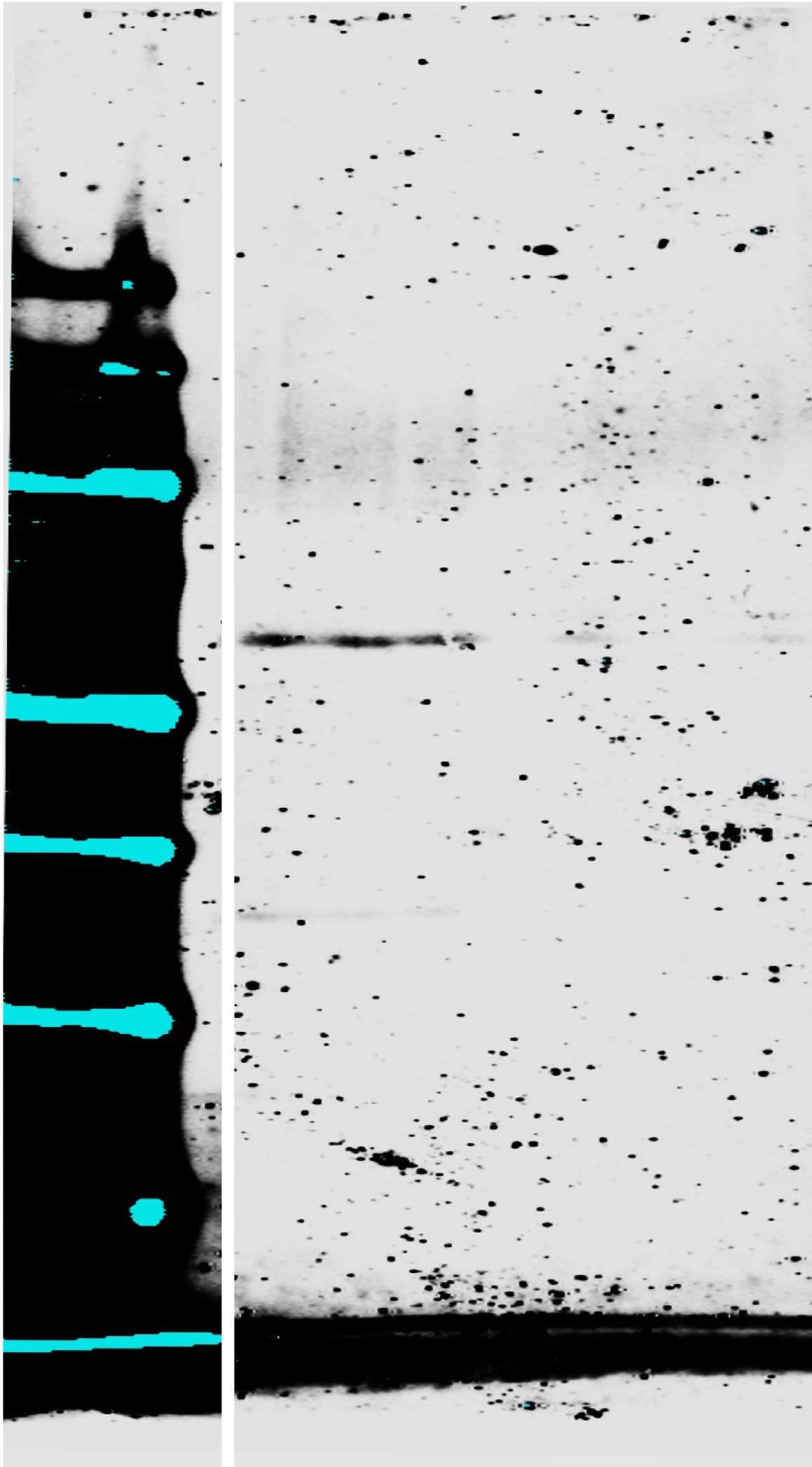

Supplement: Figure 4—source data 2. [file elife-91002-fig4-data2.zip › Figure 4 - Source data 2/Figure_4D- Source data_anti-AMPK_raw data.jpg]

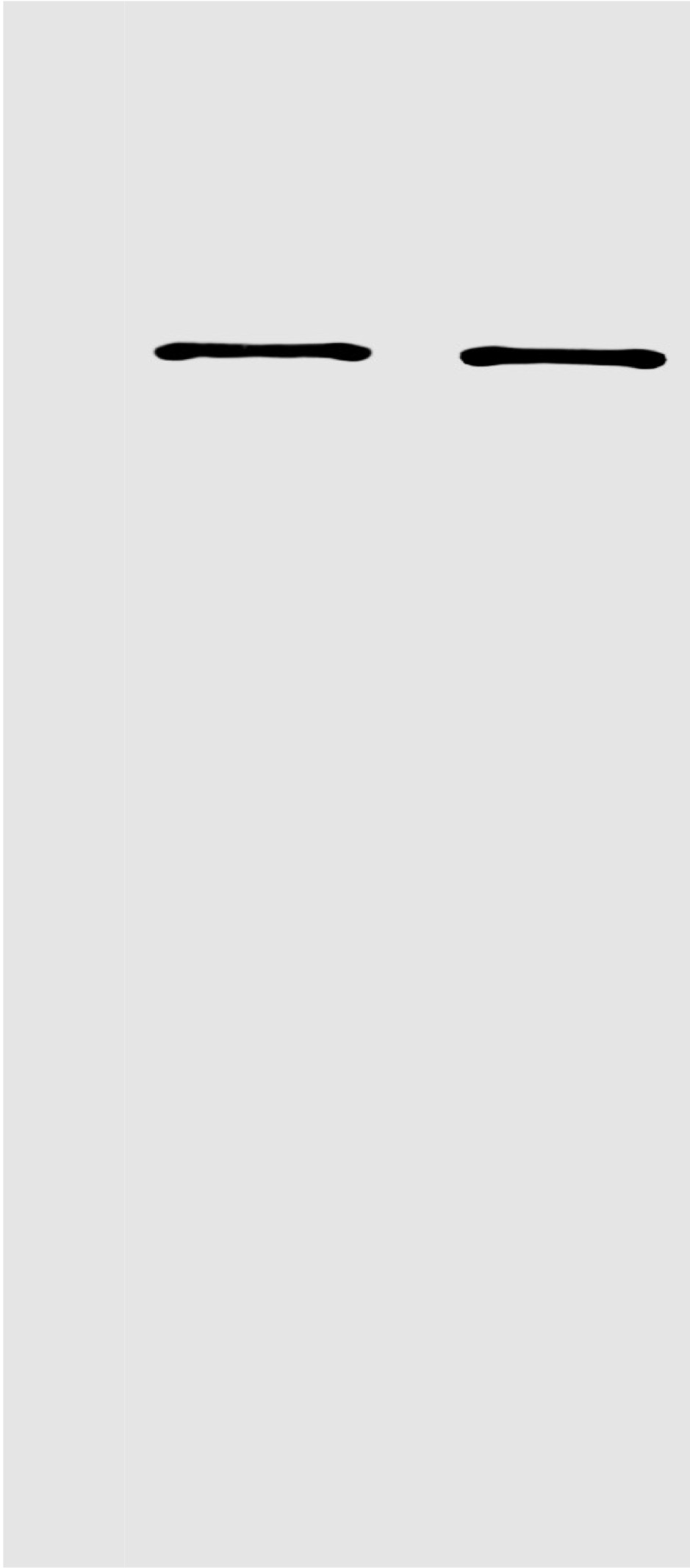

Supplement: Figure 4—source data 2. [file elife-91002-fig4-data2.zip › Figure 4 - Source data 2/Figure_4D- Source data_anti-Actin_raw data2.jpg]

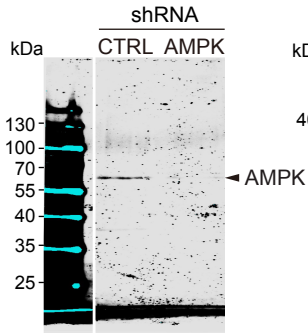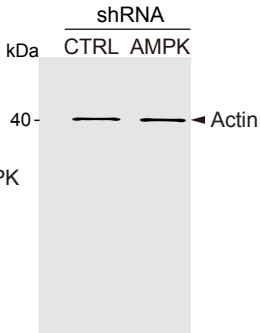

Supplement: Figure 4—source data 2. [file elife-91002-fig4-data2.zip › Figure 4 - Source data 2/Figure_4D_uncropped.pdf]

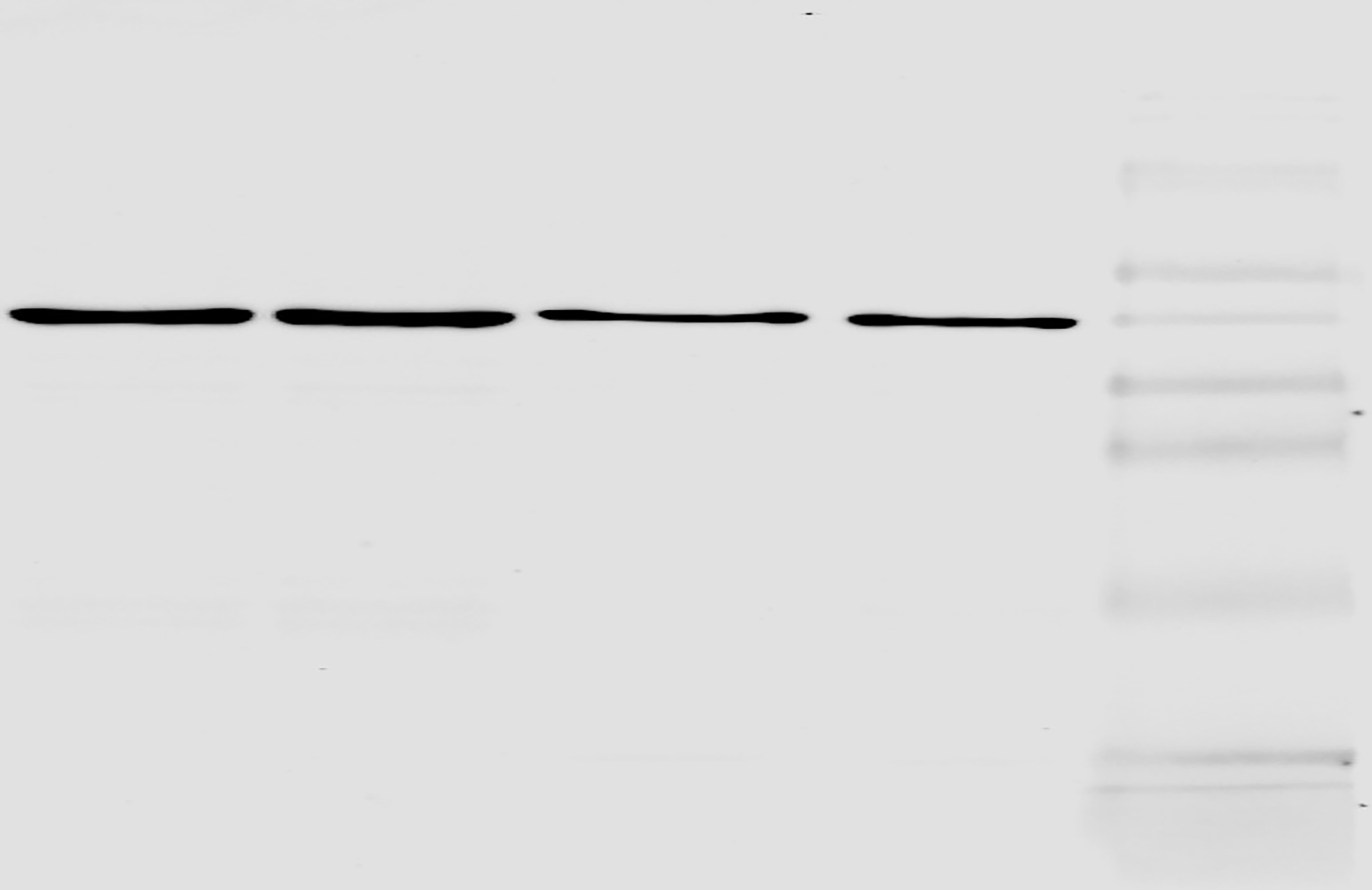

Supplement: Figure 5—source data 1. [file elife-91002-fig5-data1.zip › Figure 5 - Source data 1/Figure_5A- Source data_anti-Actin_raw data.jpg]

| MG132   |      |   |      |   |
|---------|------|---|------|---|
| shRNA:  | CTRL |   | AMPK |   |
| Baf A1: | -    | + | -    | + |

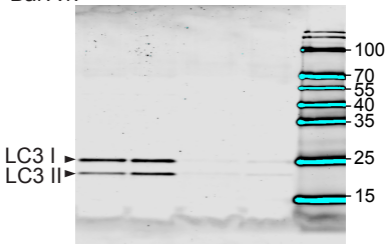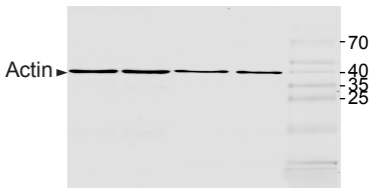

Supplement: Figure 5—source data 1. [file elife-91002-fig5-data1.zip › Figure 5 - Source data 1/Figure_5A_uncropped.pdf]

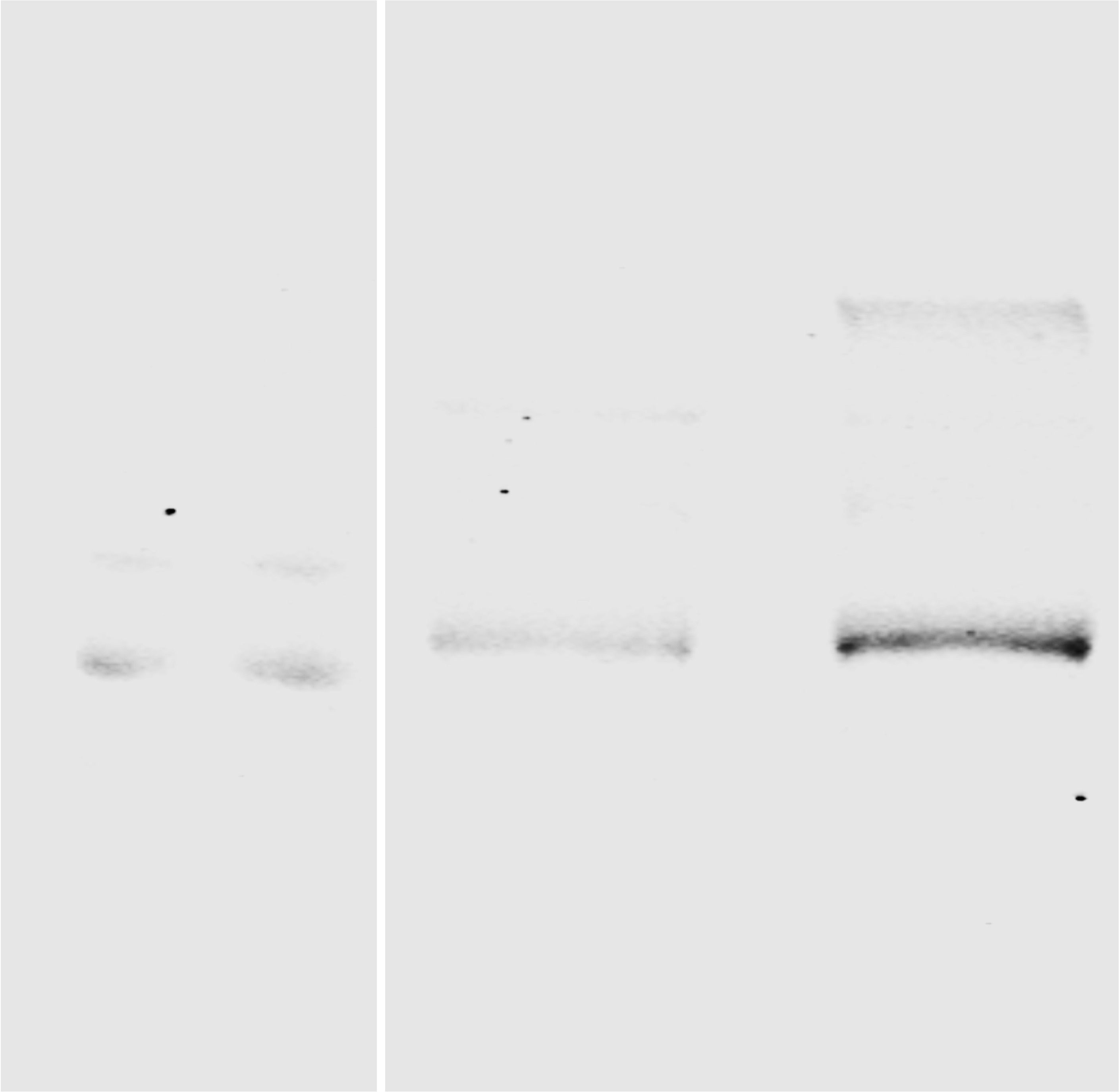

Supplement: Figure 5—source data 2. [file elife-91002-fig5-data2.zip › Figure 5 - Source data 2/Figure_5B- Source data_anti-pULK1_raw data2.jpg]

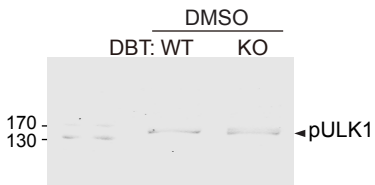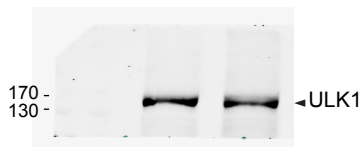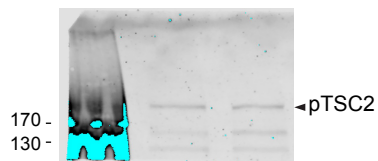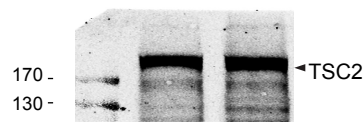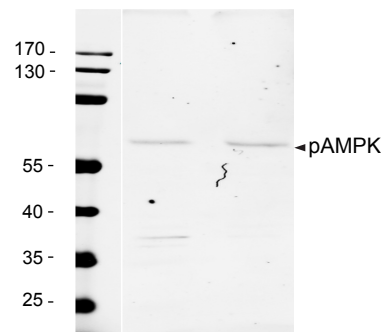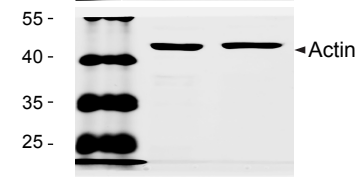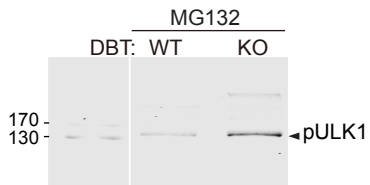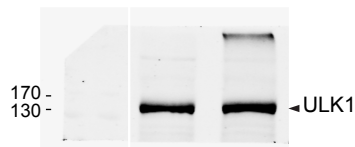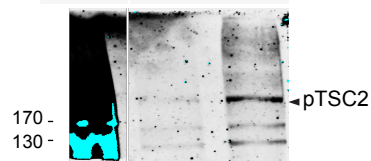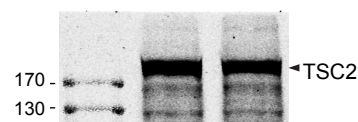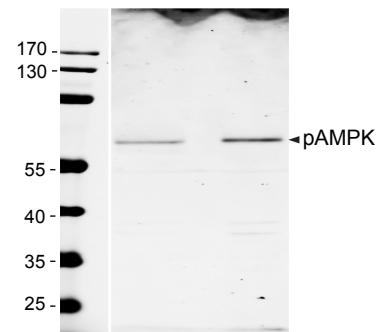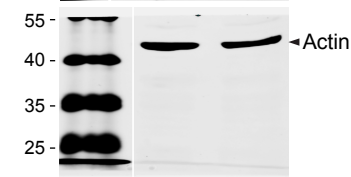

Supplement: Figure 5—source data 2. [file elife-91002-fig5-data2.zip › Figure 5 - Source data 2/Figure_5B_uncropped.pdf]

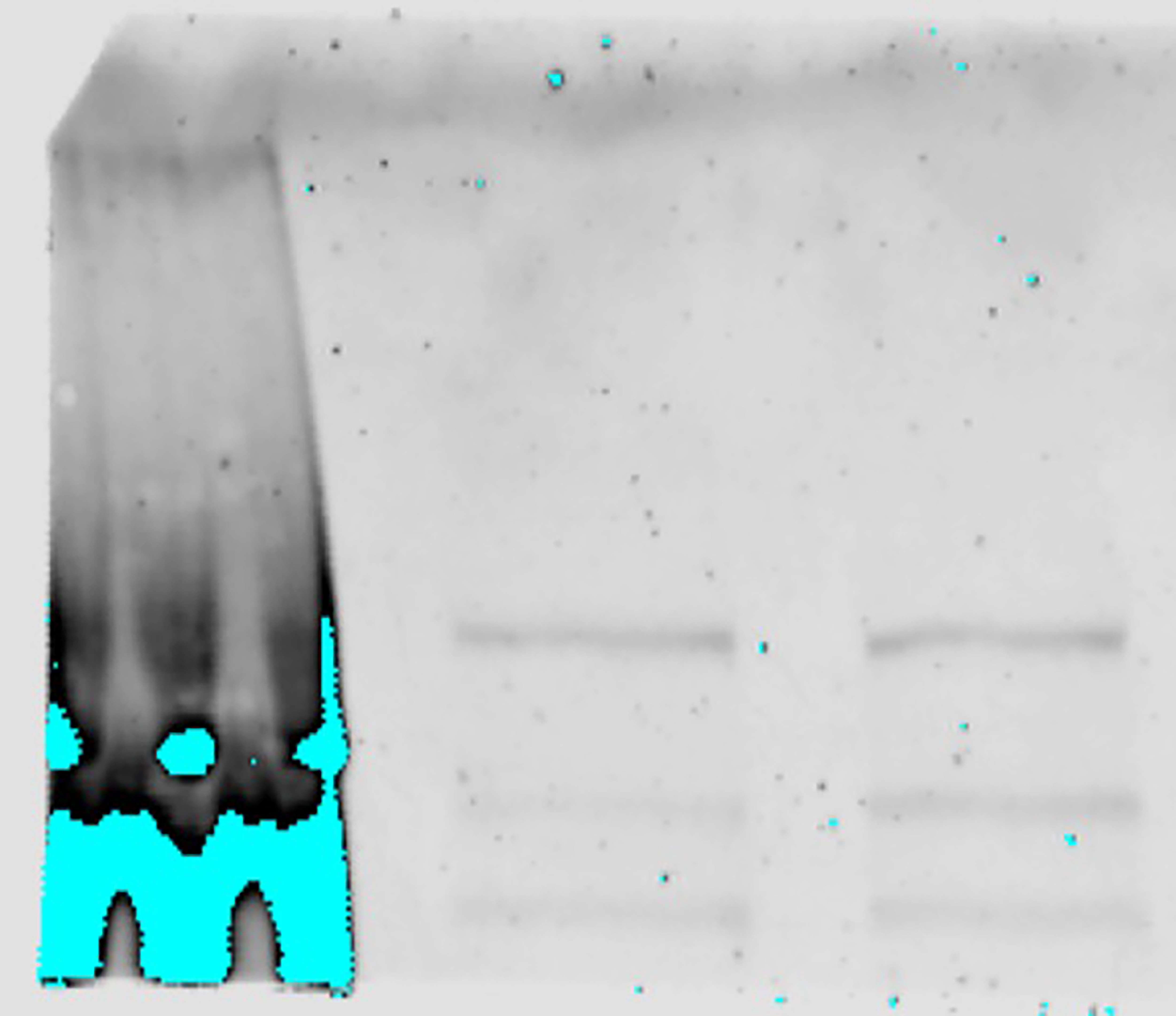

Supplement: Figure 5—source data 2. [file elife-91002-fig5-data2.zip › Figure 5 - Source data 2/Figure_5B- Source data_anti-pTSC2_raw data.jpg]

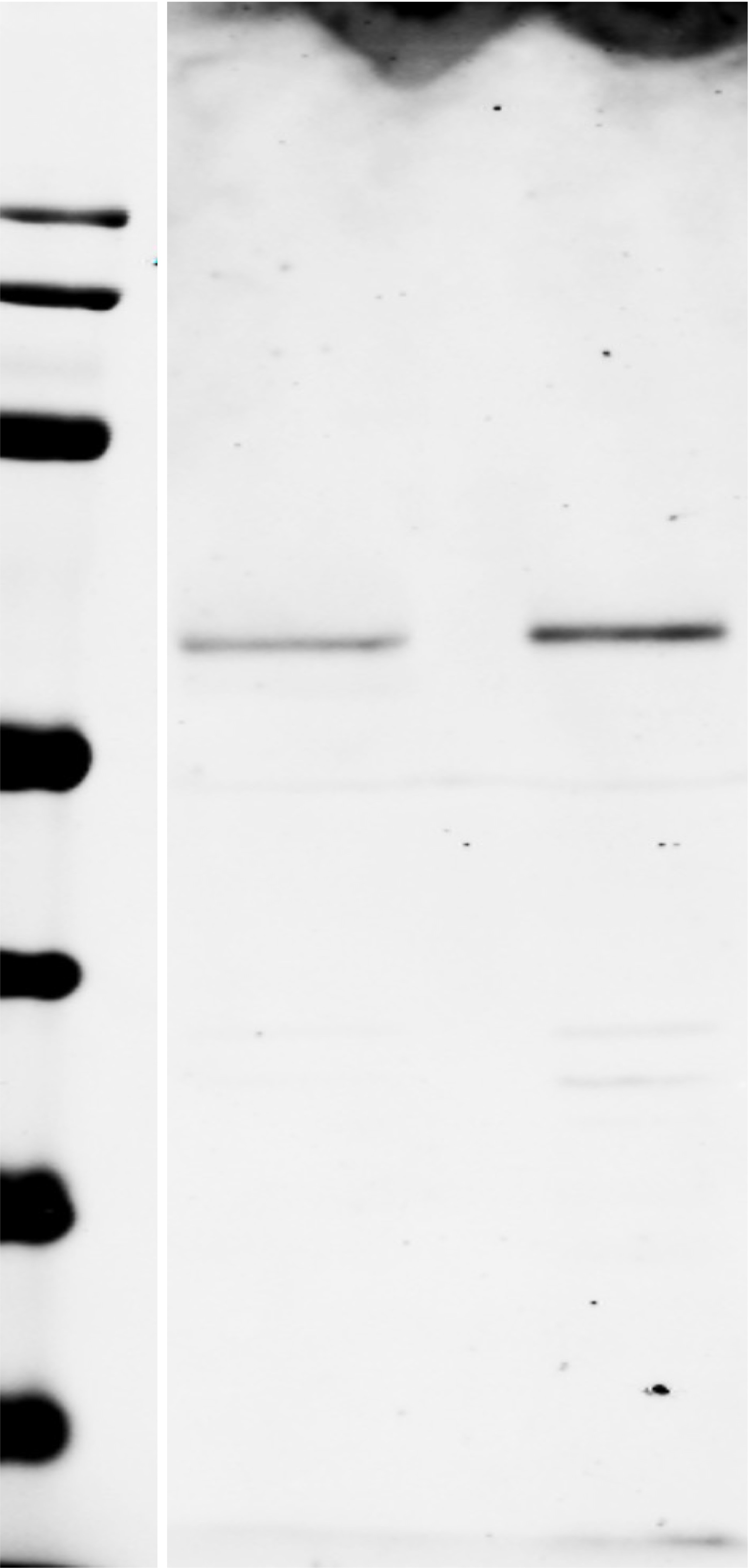

Supplement: Figure 5—source data 2. [file elife-91002-fig5-data2.zip › Figure 5 - Source data 2/Figure_5B- Source data_anti-pAMPK_raw data2.jpg]

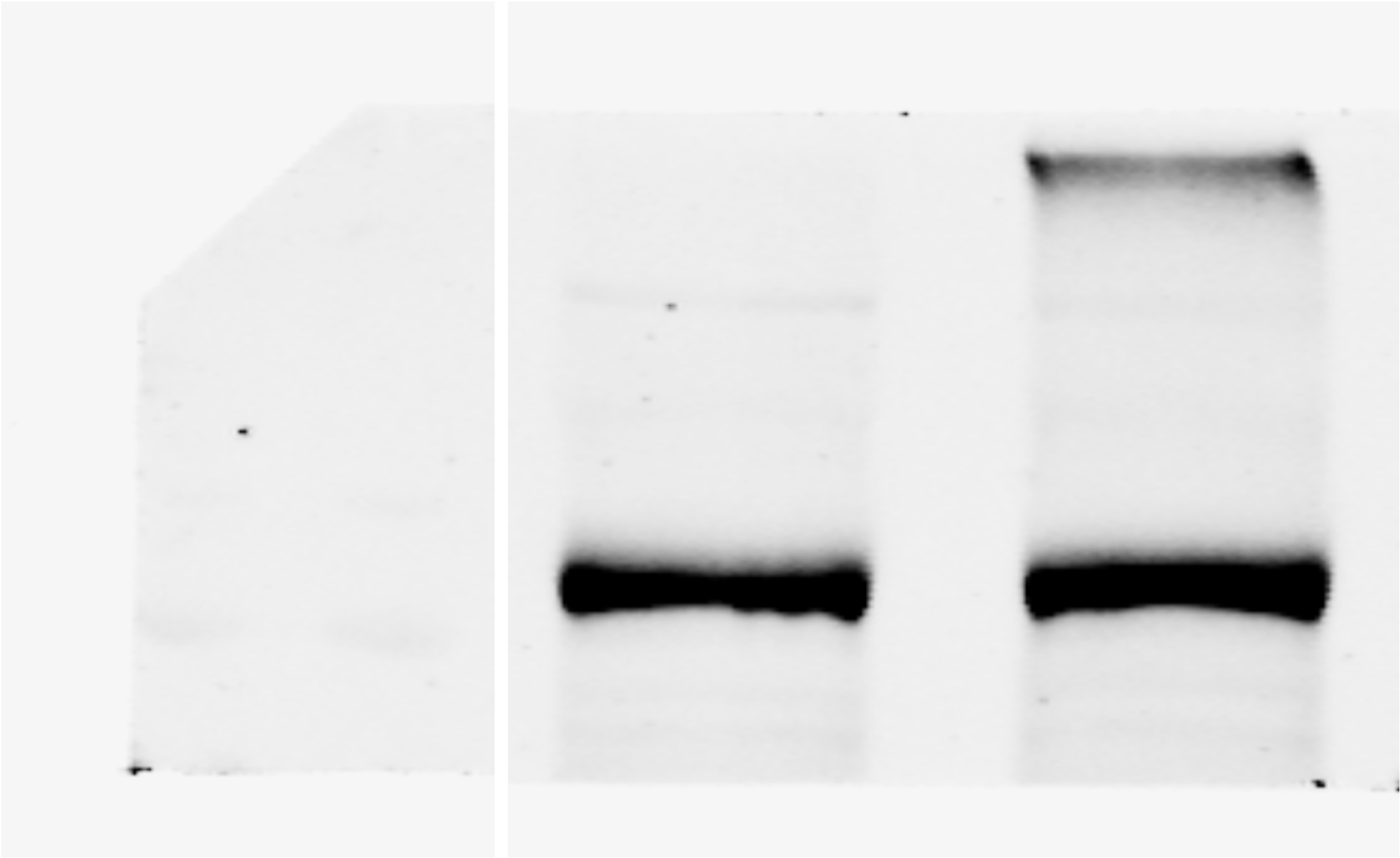

Supplement: Figure 5—source data 2. [file elife-91002-fig5-data2.zip › Figure 5 - Source data 2/Figure_5B- Source data_anti-ULK1_raw data2.jpg]

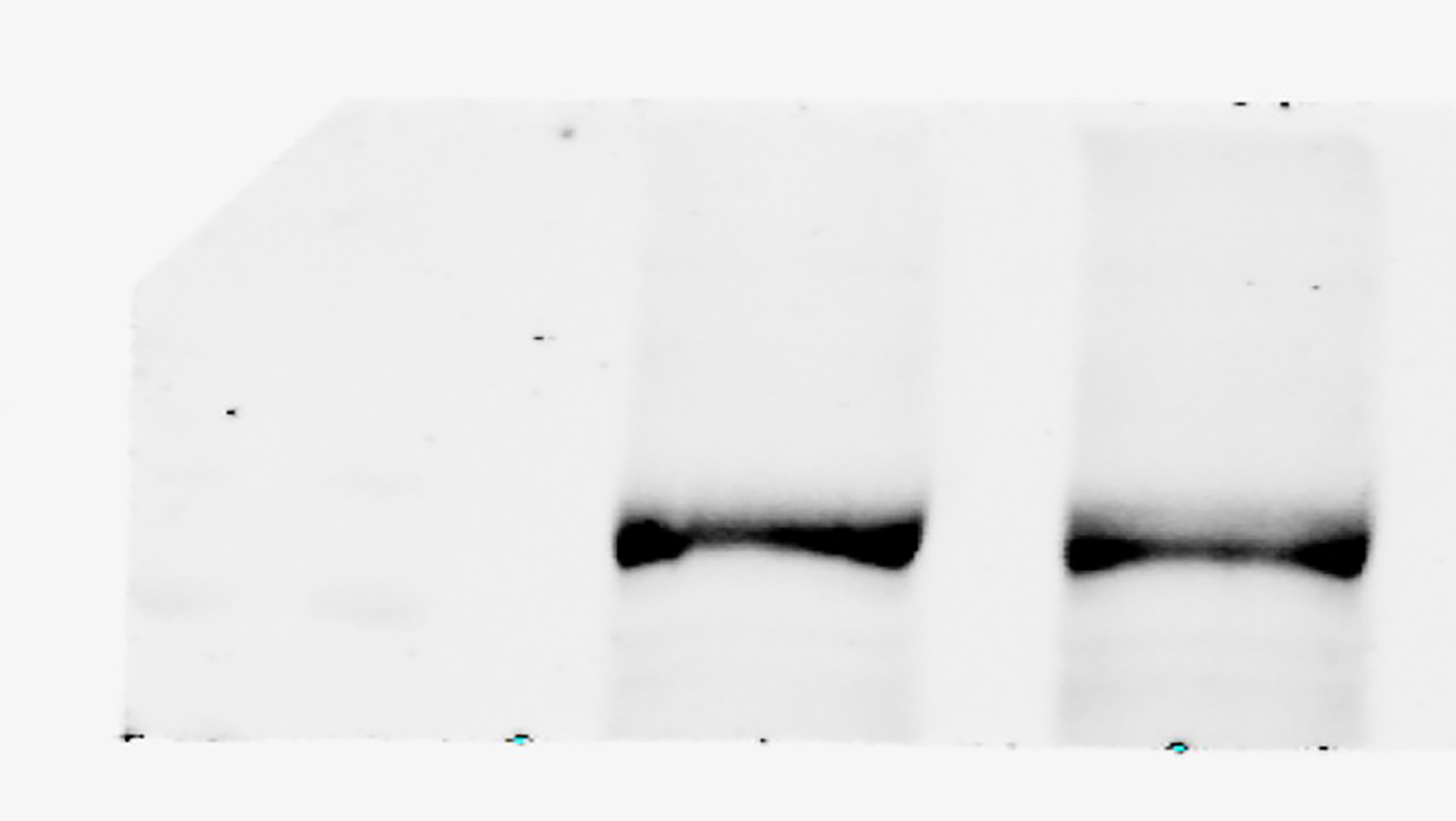

Supplement: Figure 5—source data 2. [file elife-91002-fig5-data2.zip › Figure 5 - Source data 2/Figure_5B- Source data_anti-ULK1_raw data.jpg]

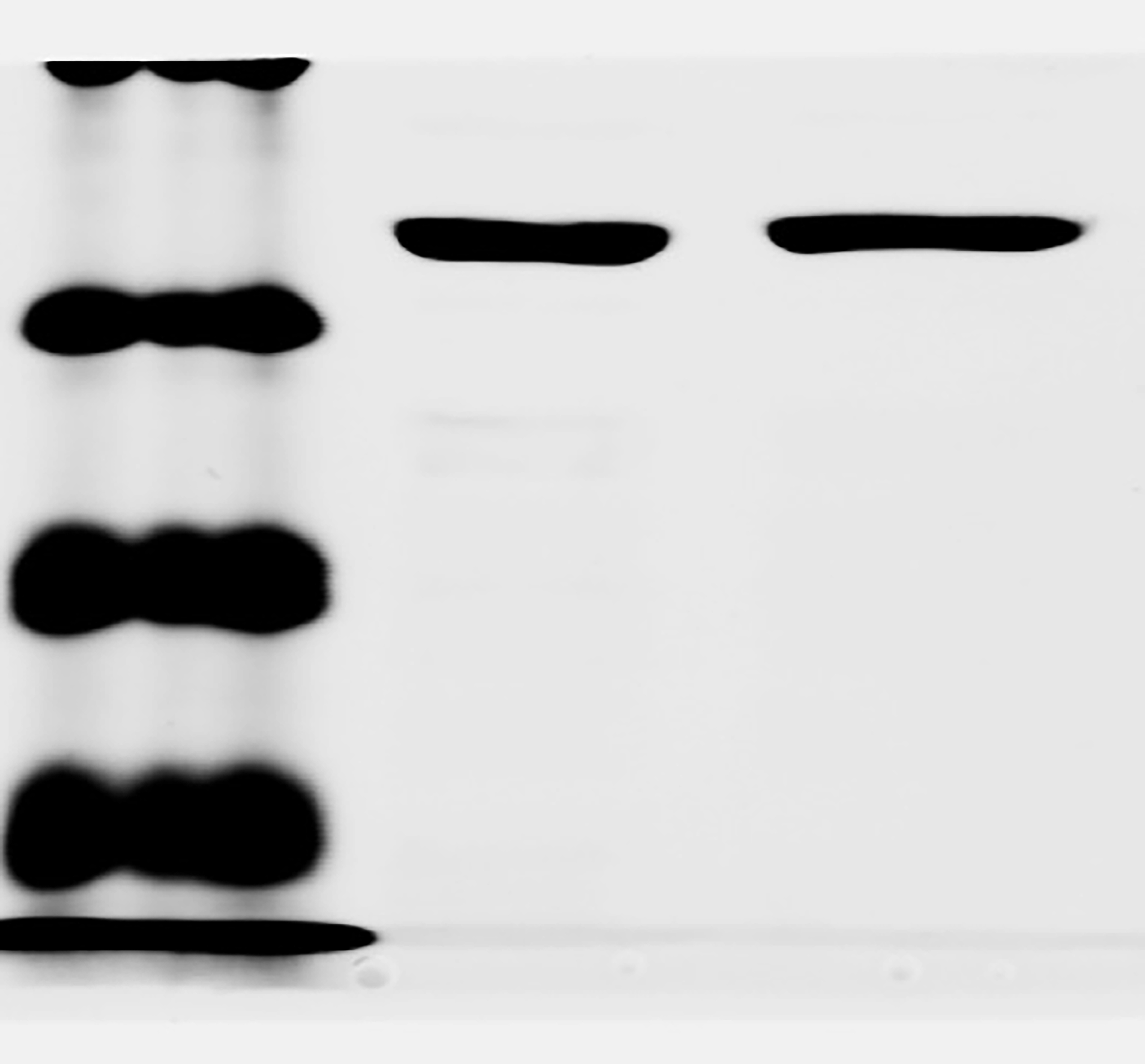

Supplement: Figure 5—source data 2. [file elife-91002-fig5-data2.zip › Figure 5 - Source data 2/Figure_5B- Source data_anti-Actin_raw data.jpg]

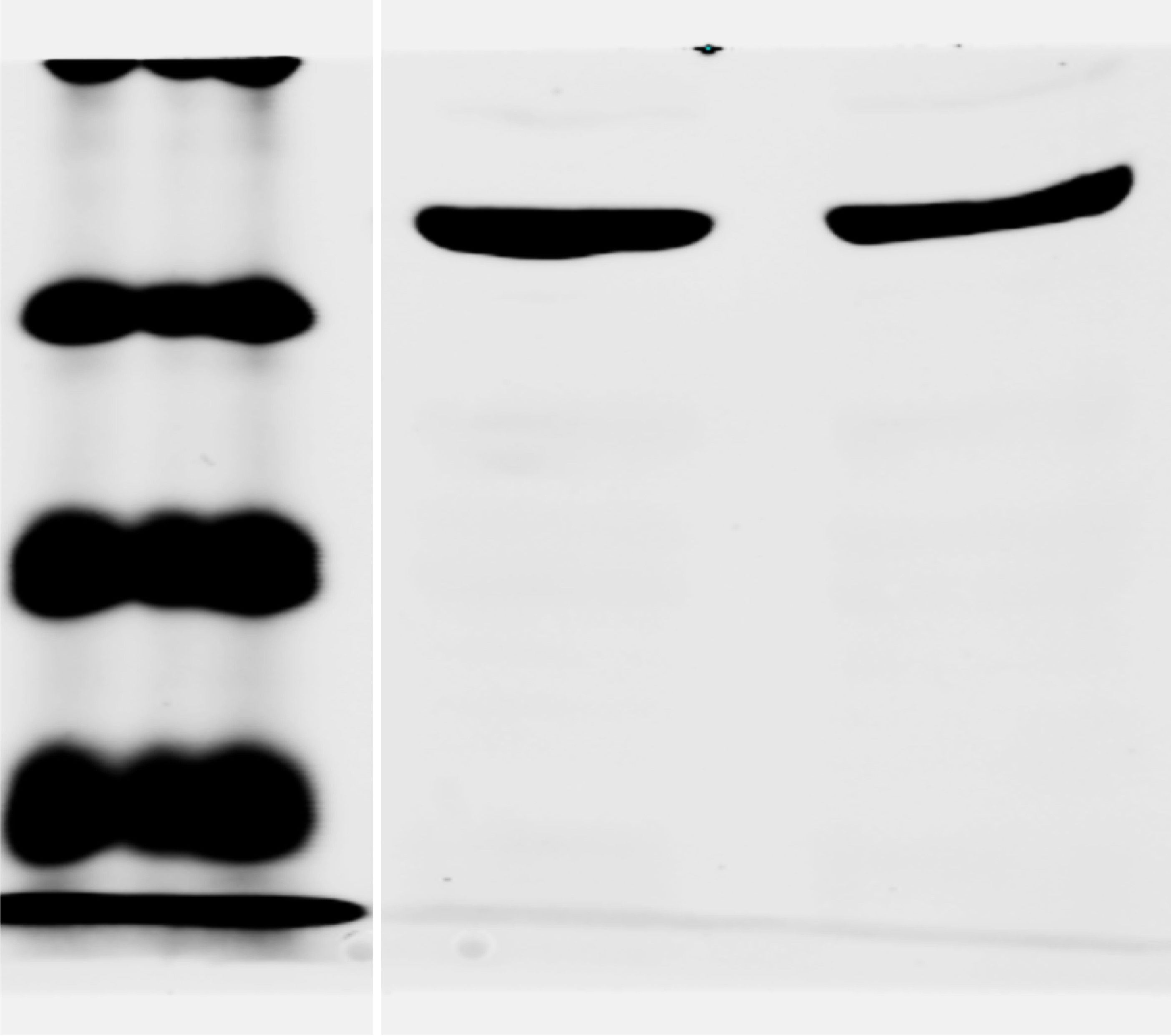

Supplement: Figure 5—source data 2. [file elife-91002-fig5-data2.zip › Figure 5 - Source data 2/Figure_5B- Source data_anti-Actin_raw data2.jpg]

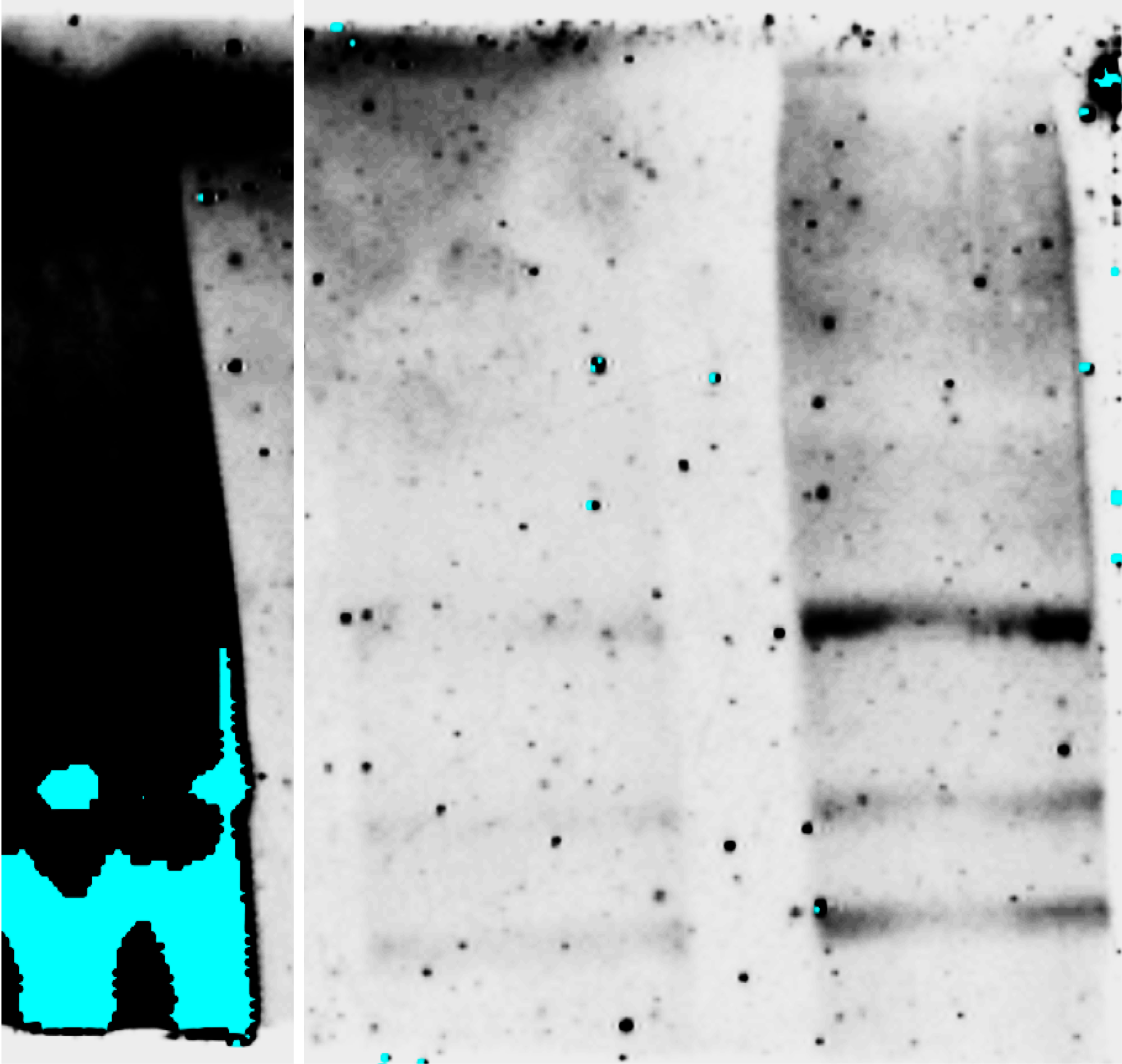

Supplement: Figure 5—source data 2. [file elife-91002-fig5-data2.zip › Figure 5 - Source data 2/Figure_5B- Source data_anti-pTSC2_raw data2.png]

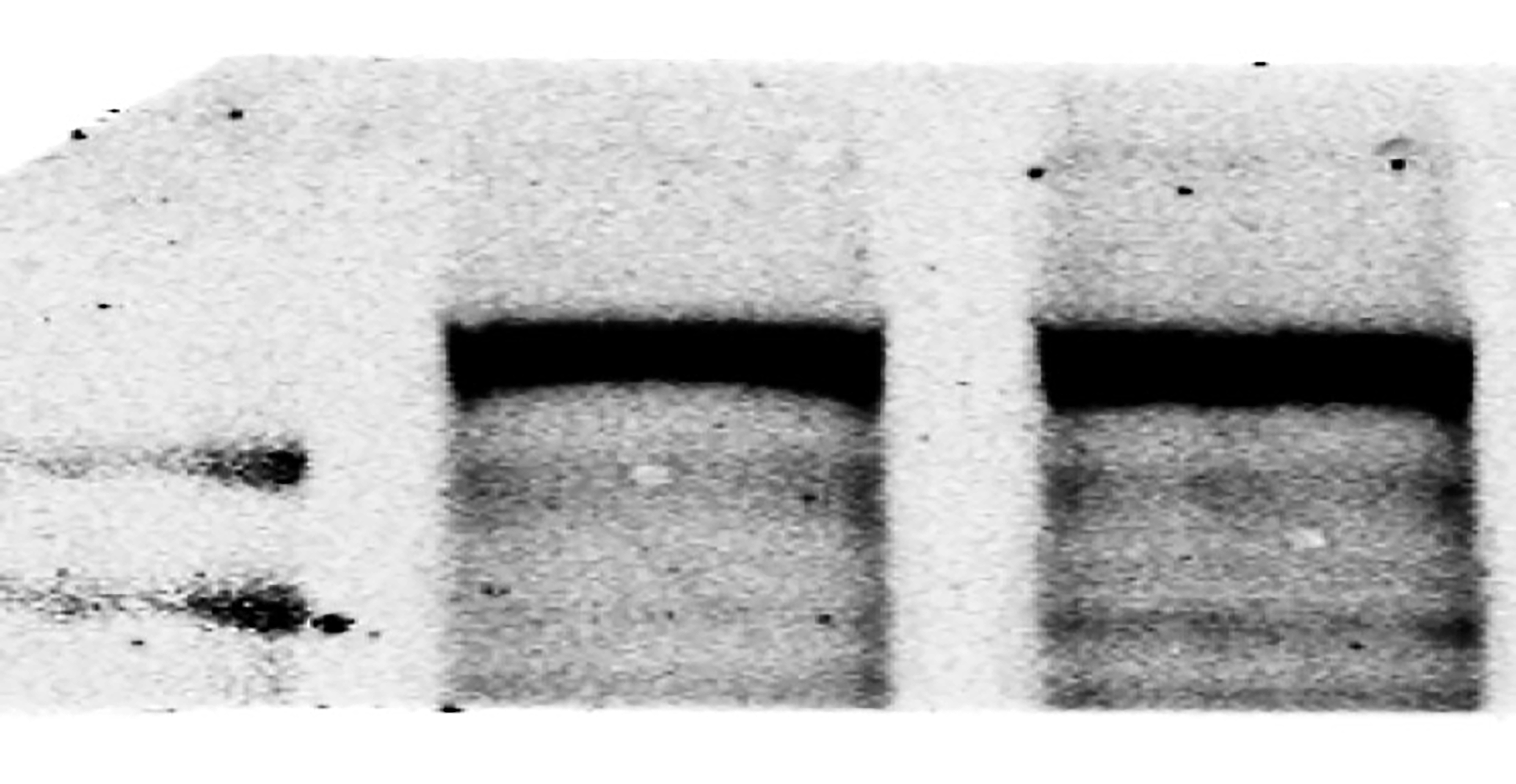

Supplement: Figure 5—source data 2. [file elife-91002-fig5-data2.zip › Figure 5 - Source data 2/Figure_5B- Source data_anti-TSC2_raw data.jpg]

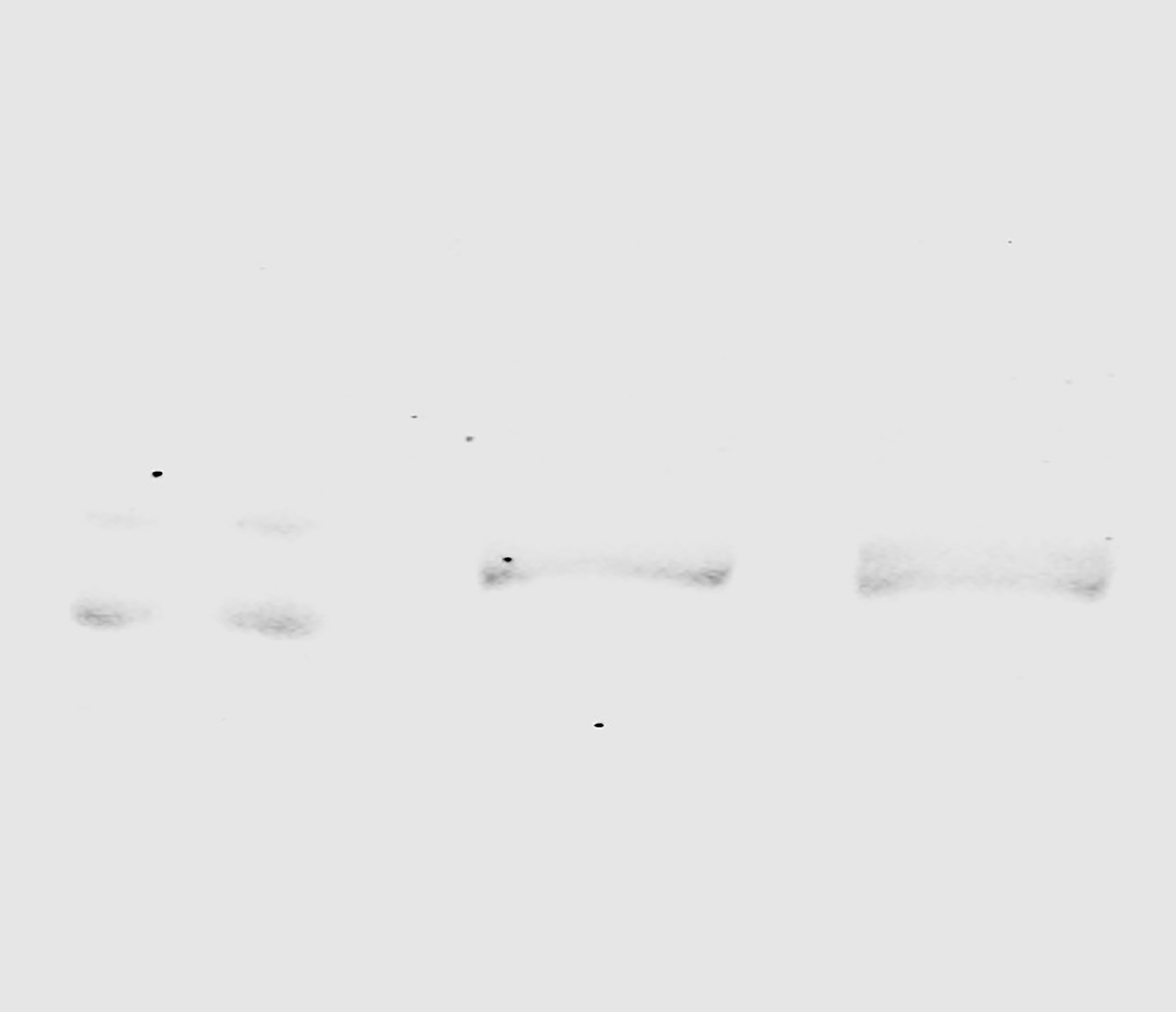

Supplement: Figure 5—source data 2. [file elife-91002-fig5-data2.zip › Figure 5 - Source data 2/Figure_5B- Source data_anti-pULK1_raw data.jpg]

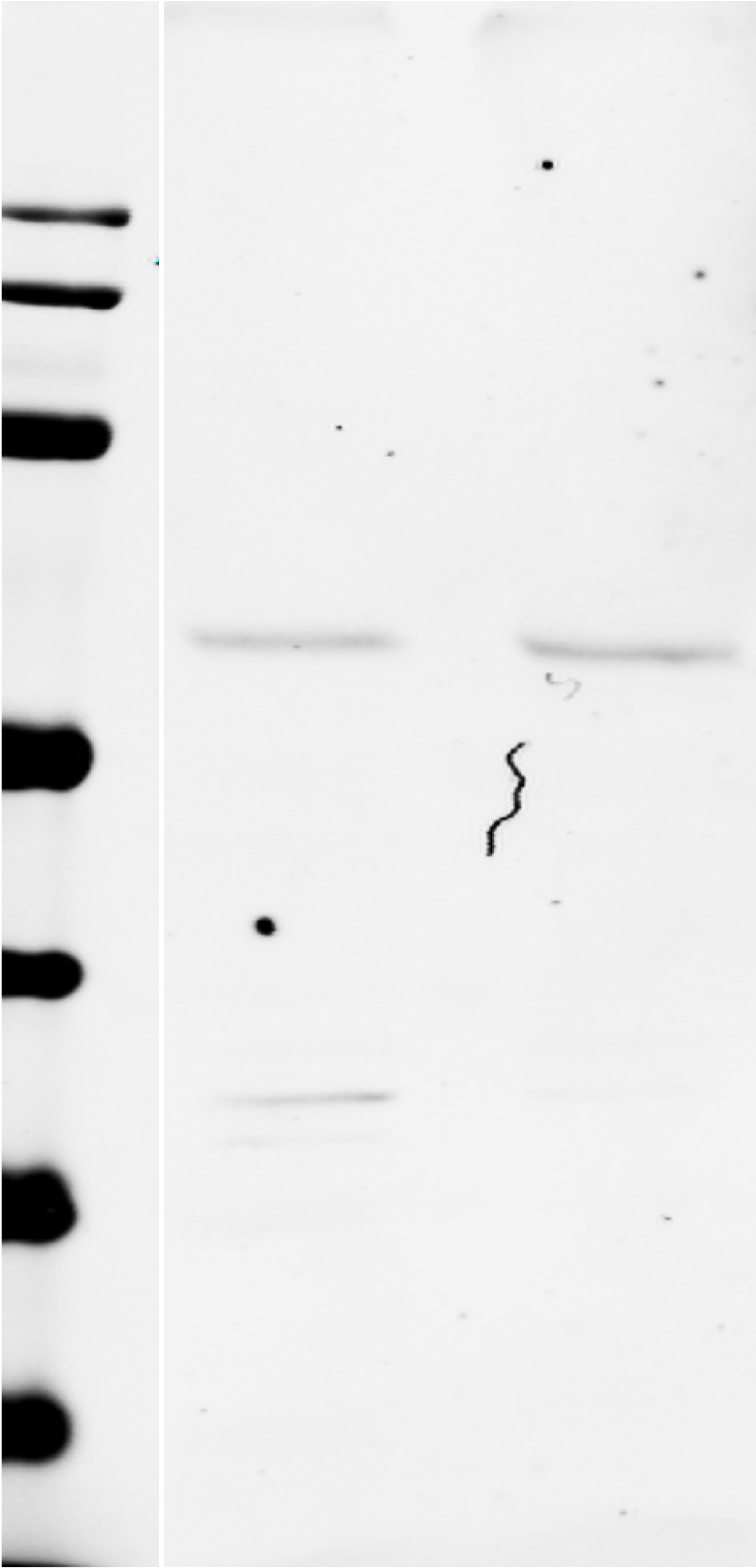

Supplement: Figure 5—source data 2. [file elife-91002-fig5-data2.zip › Figure 5 - Source data 2/Figure_5B- Source data_anti-pAMPK_raw data.jpg]

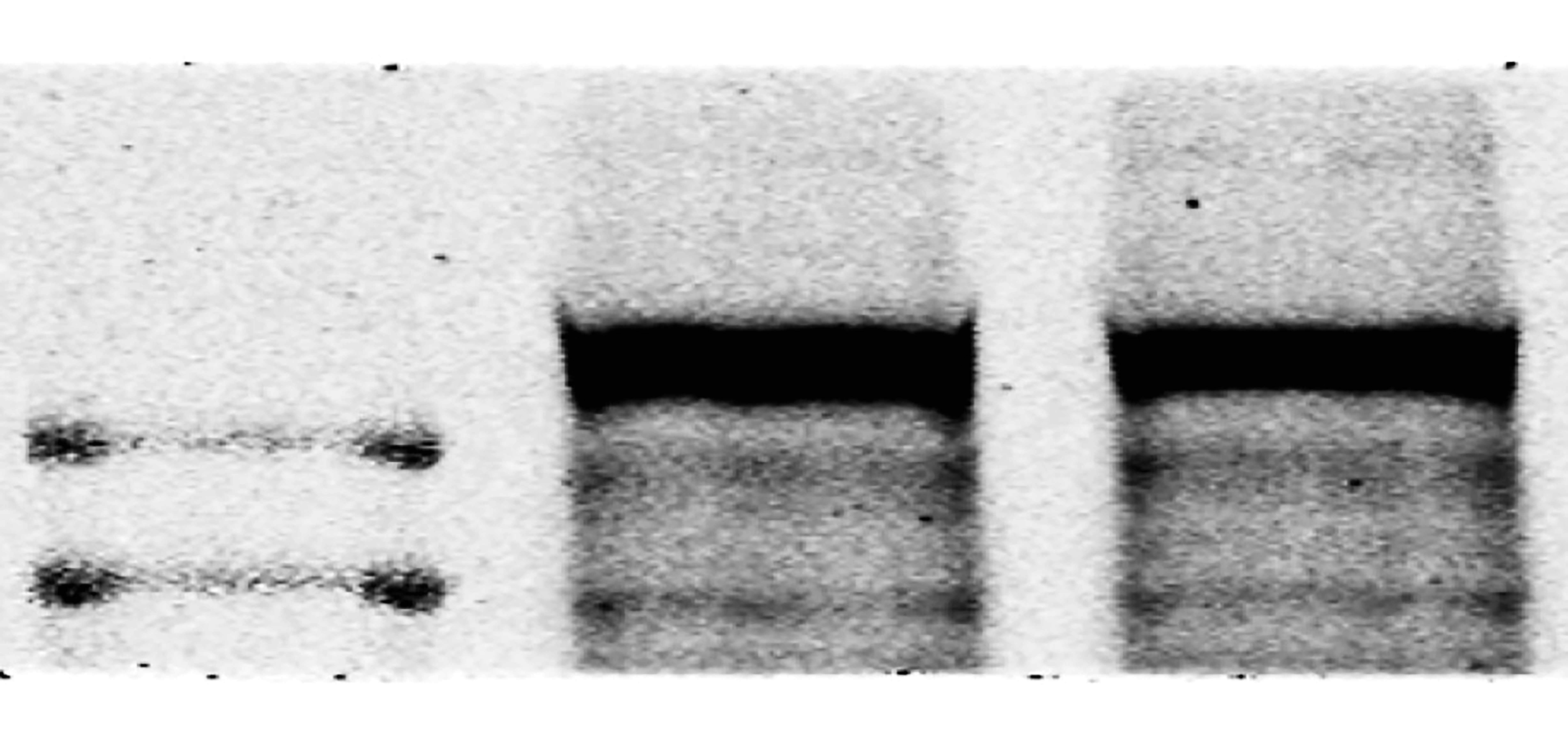

Supplement: Figure 5—source data 2. [file elife-91002-fig5-data2.zip › Figure 5 - Source data 2/Figure_5B- Source data_anti-TSC2_raw data2.jpg]

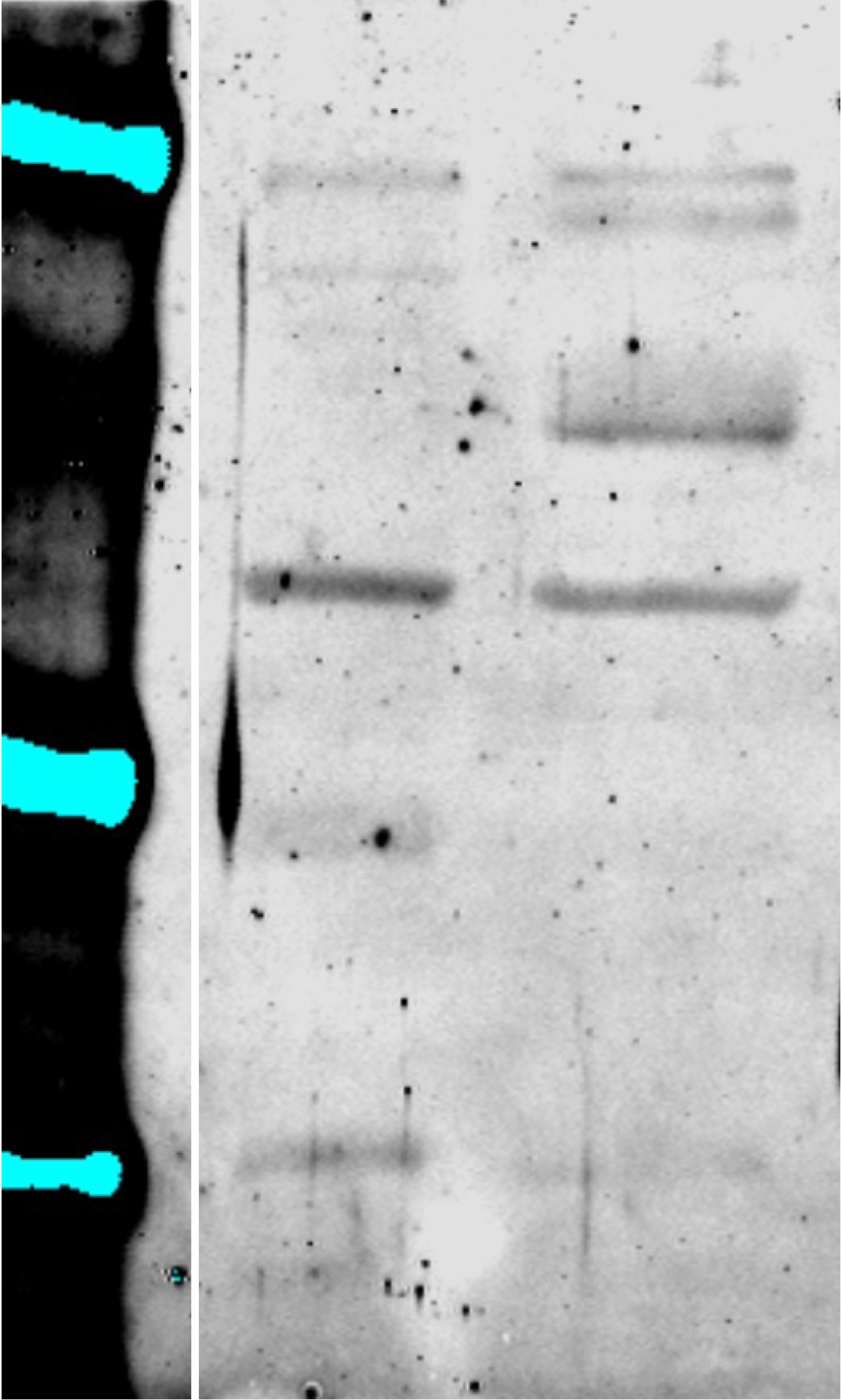

Supplement: Figure 5—source data 3. [file elife-91002-fig5-data3.zip › Figure 5 - Source data 3/Figure_5C- Source data_anti-pS6K_raw data.jpg]

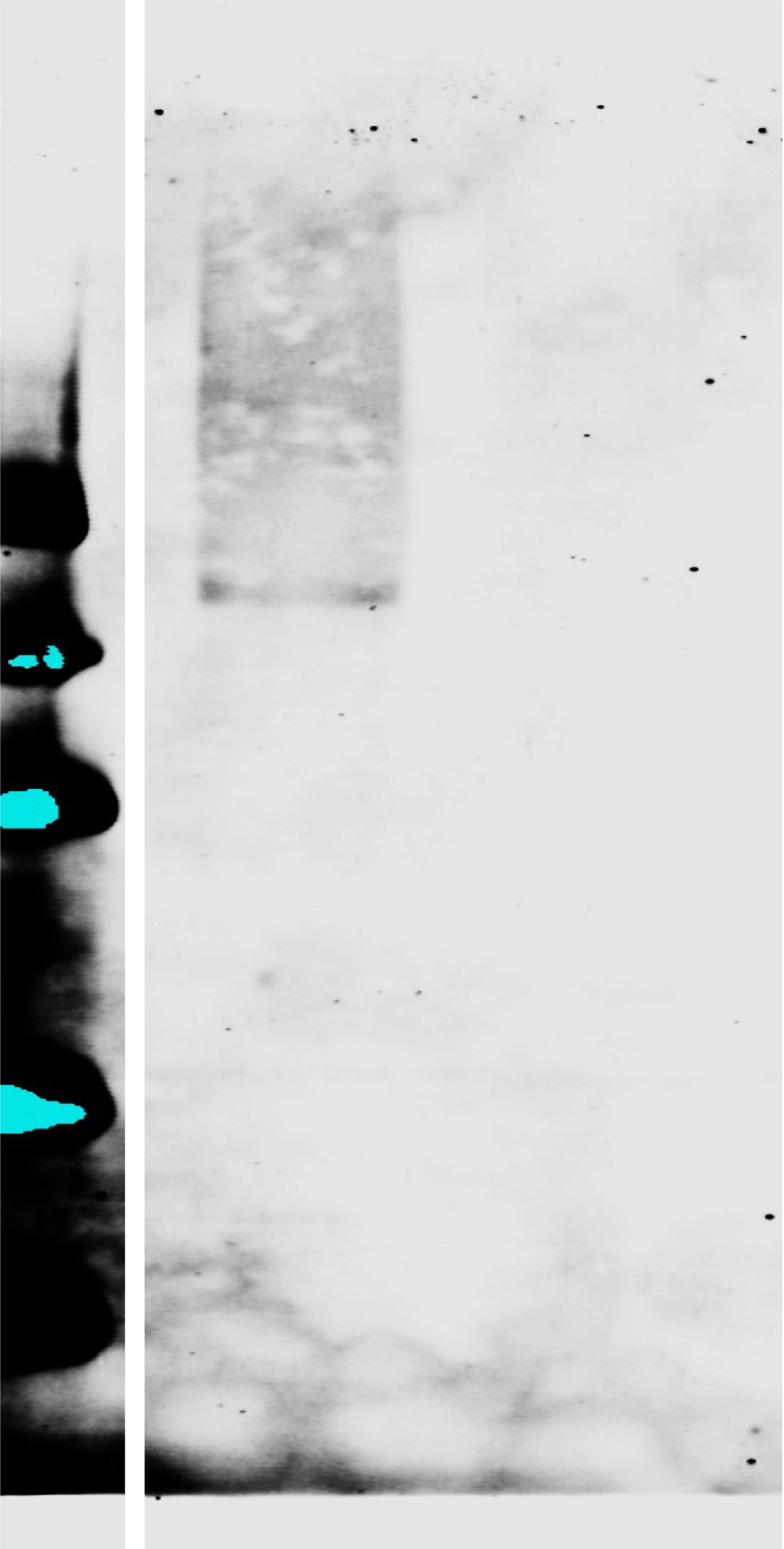

Supplement: Figure 5—source data 3. [file elife-91002-fig5-data3.zip › Figure 5 - Source data 3/Figure_5C- Source data_anti-TSC1_raw data.jpg]

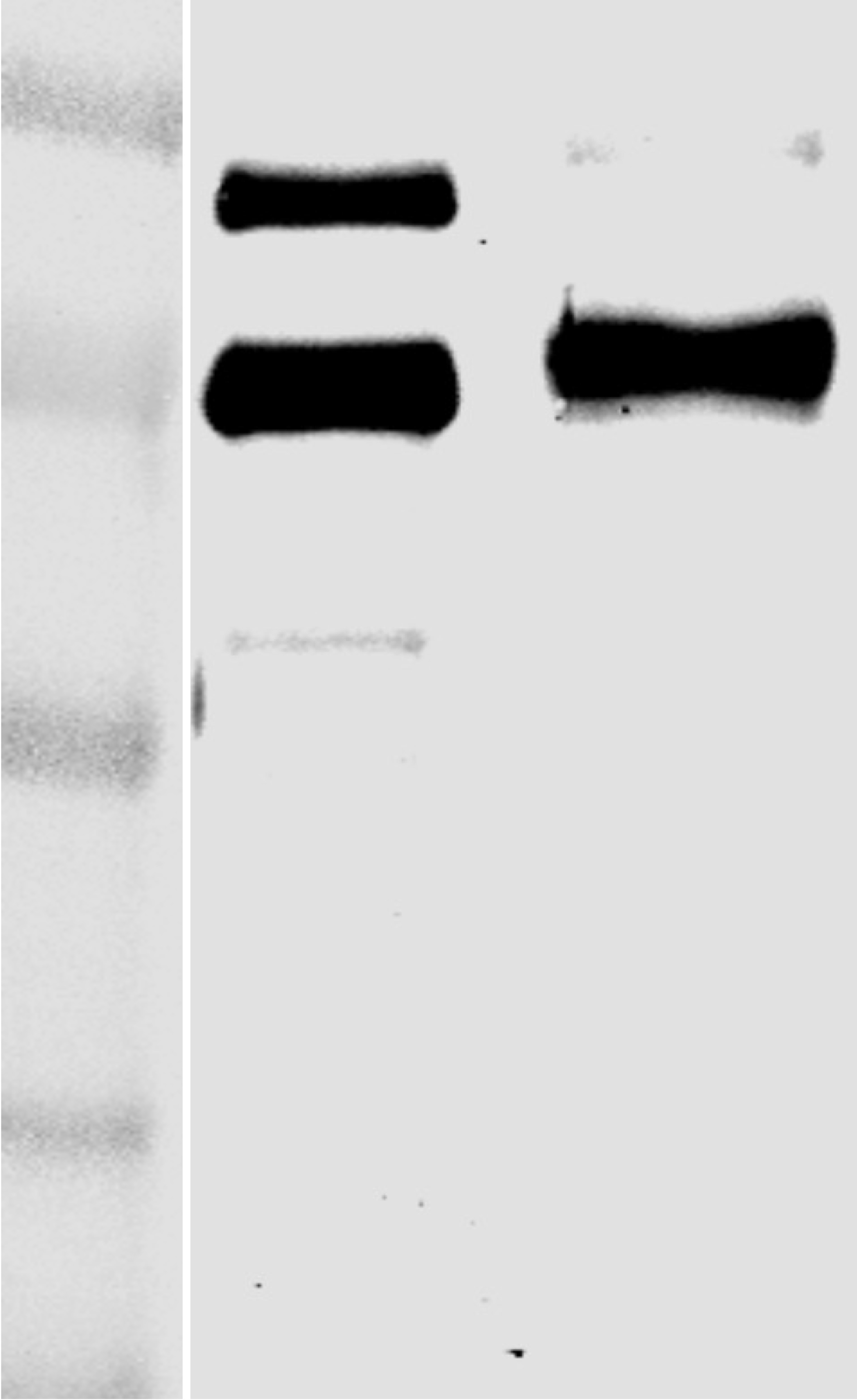

Supplement: Figure 5—source data 3. [file elife-91002-fig5-data3.zip › Figure 5 - Source data 3/Figure_5C- Source data_anti-S6K_raw data.jpg]

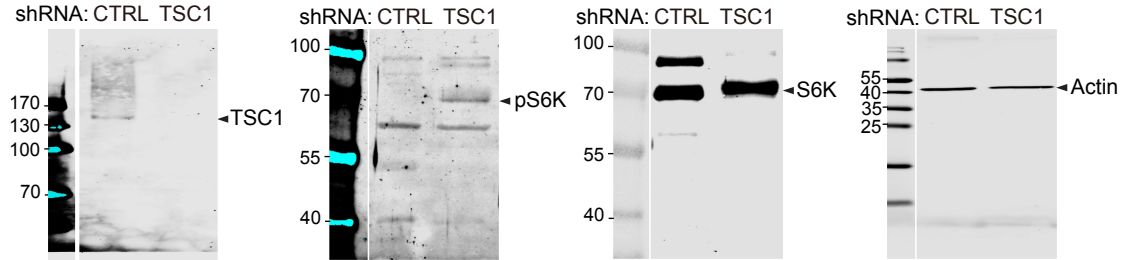

Supplement: Figure 5—source data 3. [file elife-91002-fig5-data3.zip › Figure 5 - Source data 3/Figure_5C_uncropped.pdf]

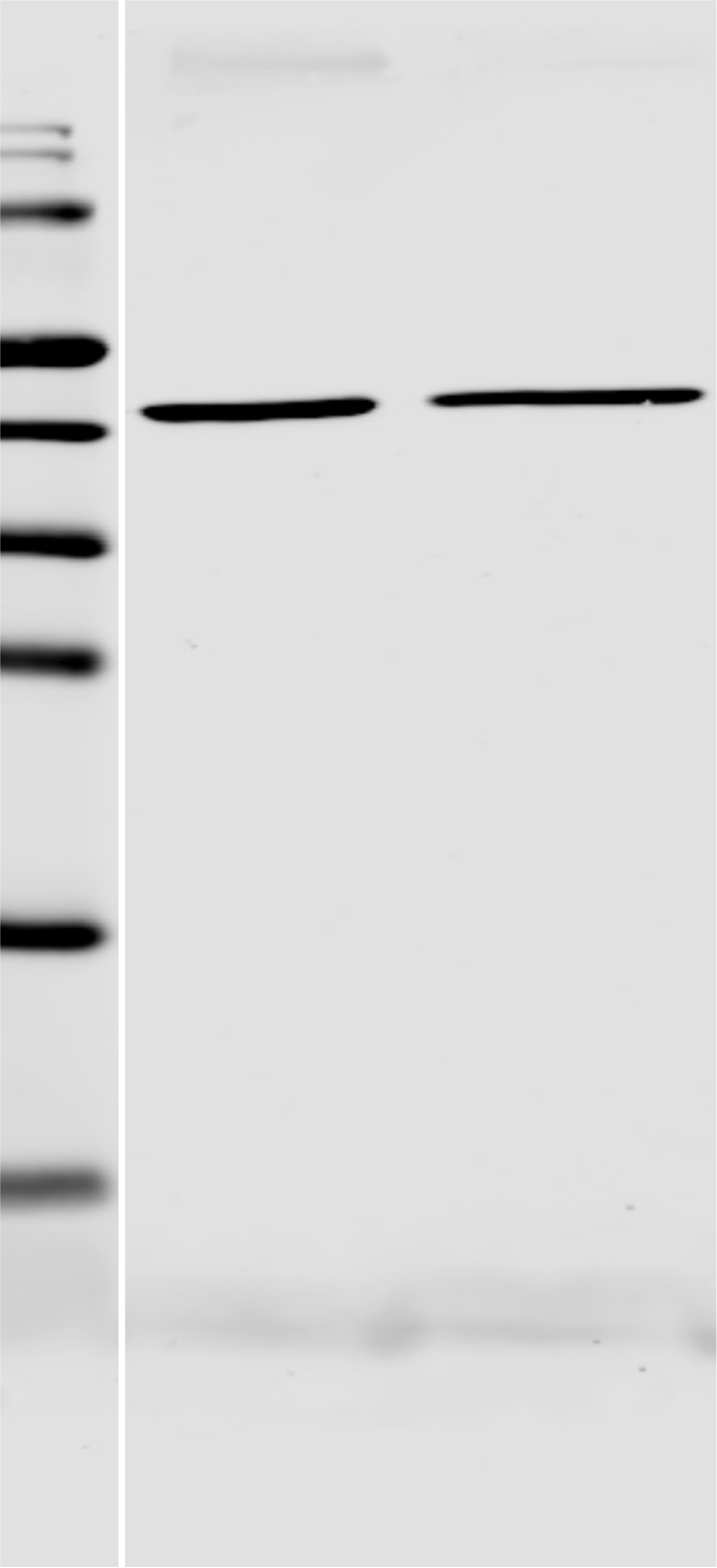

Supplement: Figure 5—source data 3. [file elife-91002-fig5-data3.zip › Figure 5 - Source data 3/Figure_5C- Source data_anti-Actin_raw data.jpg]

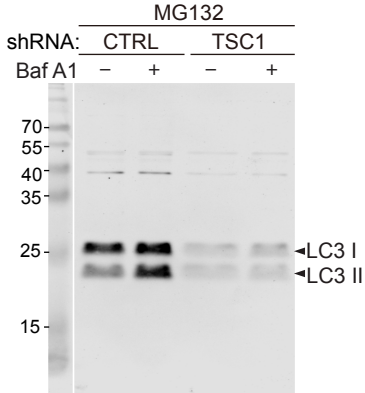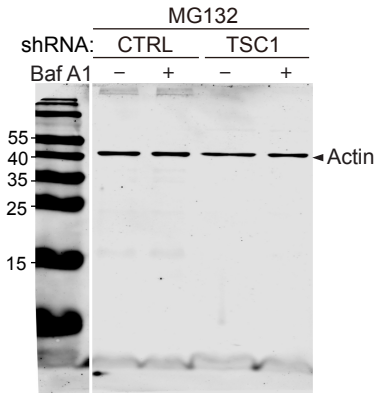

Supplement: Figure 5—source data 4. [file elife-91002-fig5-data4.zip › Figure 5 - Source data 4/Figure_5D_uncropped.pdf]

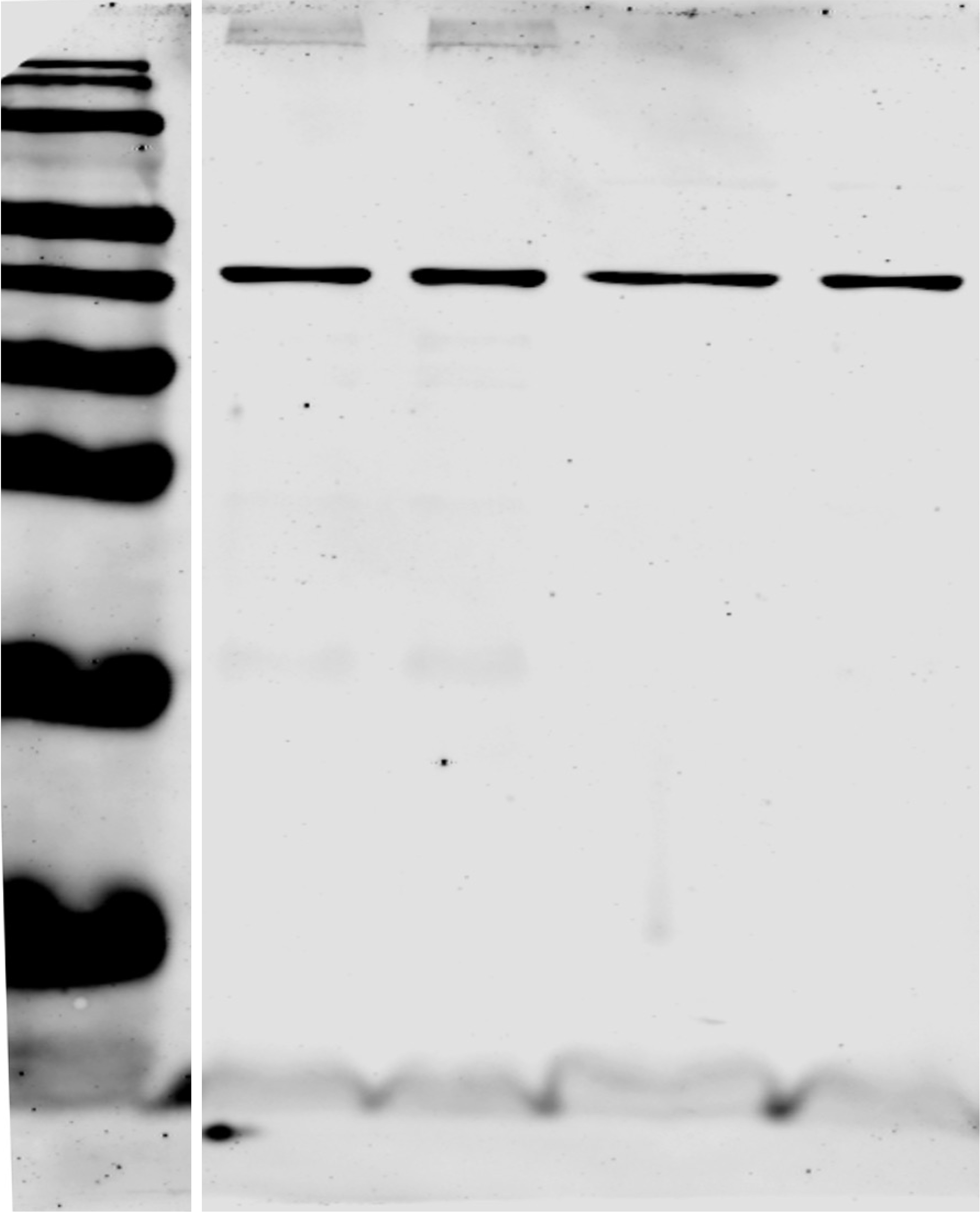

Supplement: Figure 5—source data 4. [file elife-91002-fig5-data4.zip › Figure 5 - Source data 4/Figure_5D- Source data_anti-Actin_raw data.jpg]

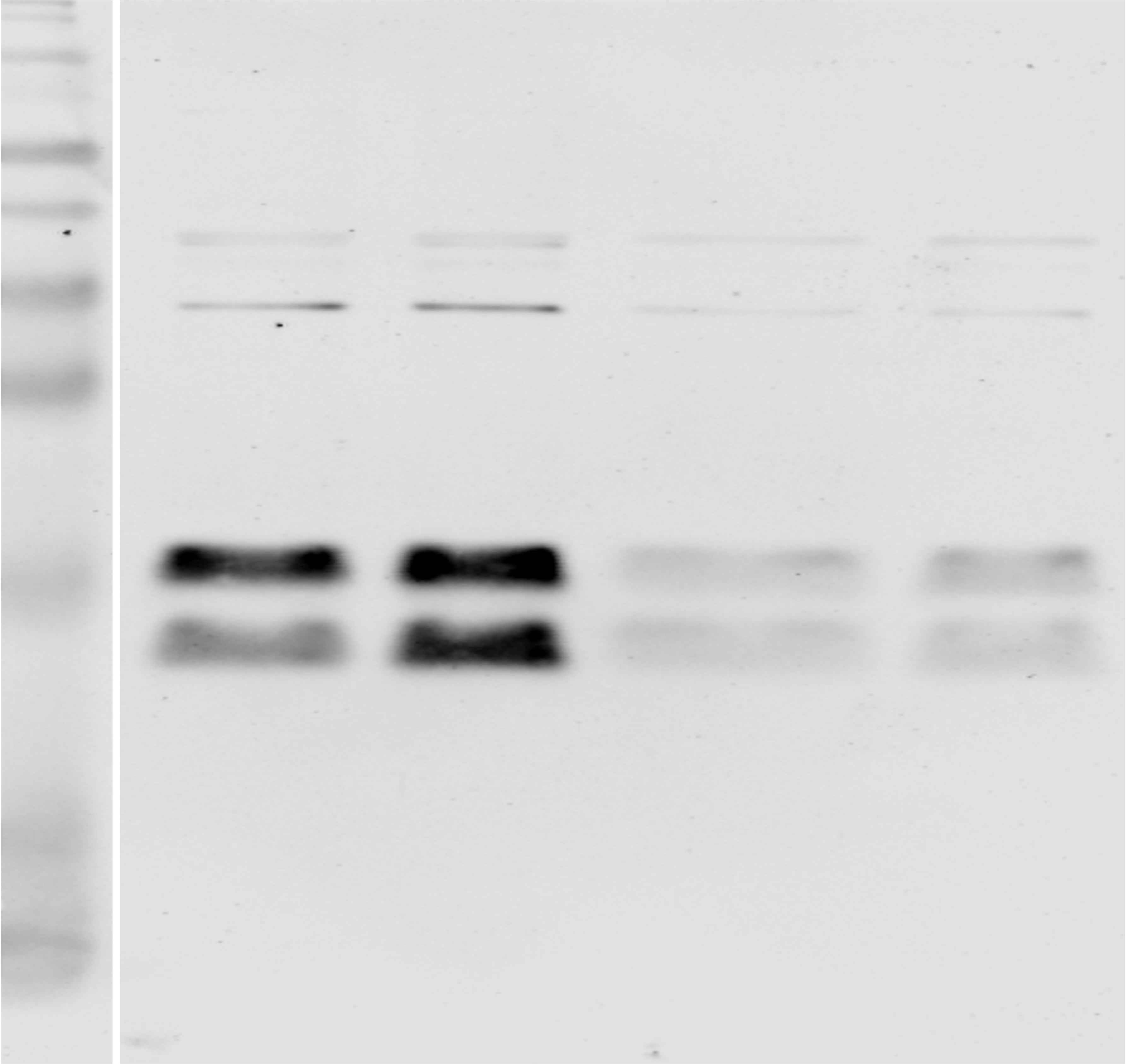

Supplement: Figure 5—source data 4. [file elife-91002-fig5-data4.zip › Figure 5 - Source data 4/Figure_5D- Source data_anti-LC3_raw data.jpg]

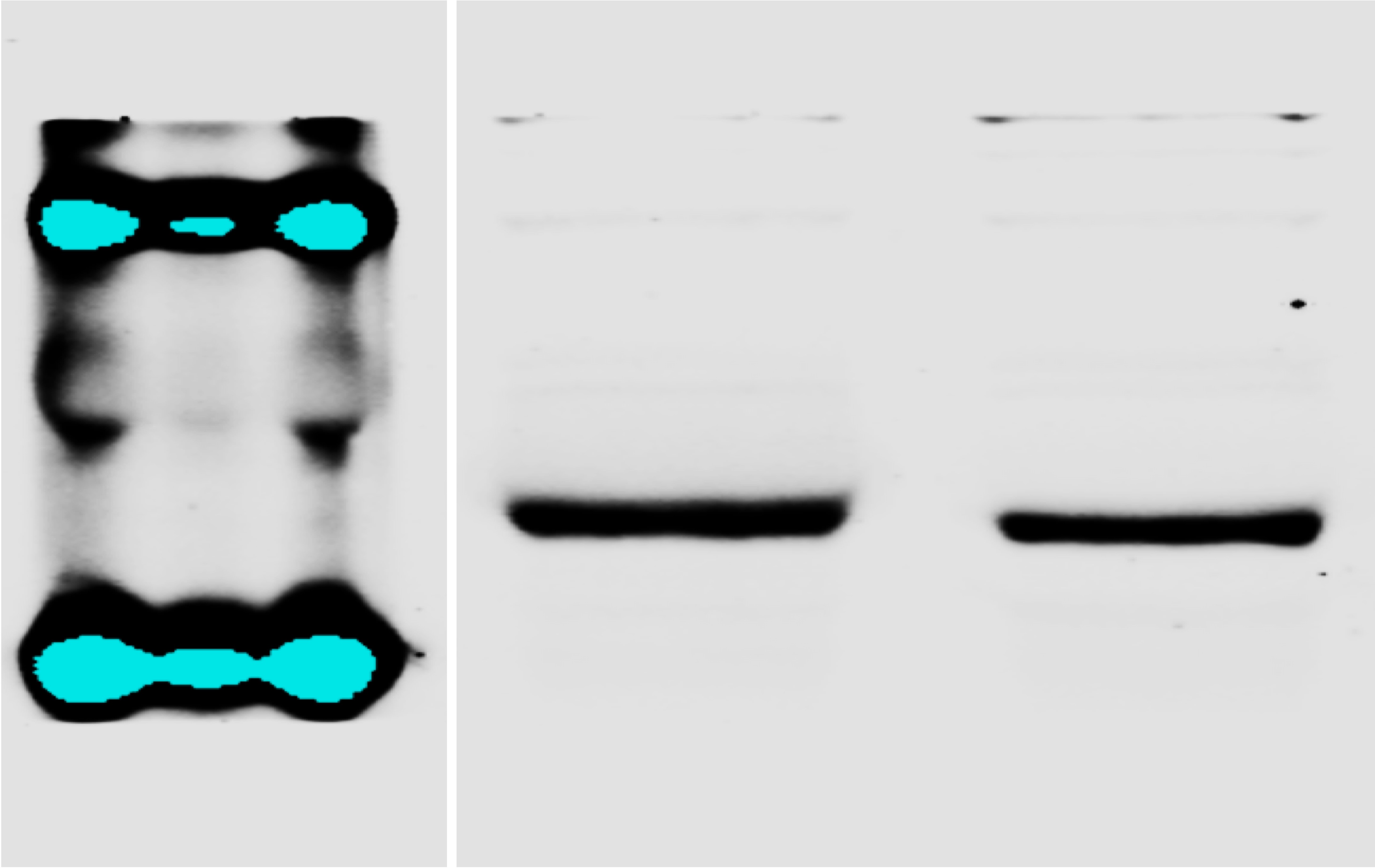

Supplement: Figure 5—figure supplement 1—source data 1. [file elife-91002-fig5-figsupp1-data1.zip › Figure 5 - Figure supplement1 - Source data 1/Figure 5 - Figure supplement1A - Source data_anti-AMPK_raw data.jpg]

VEH      Comp.C

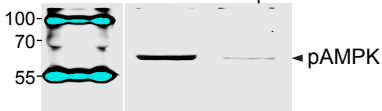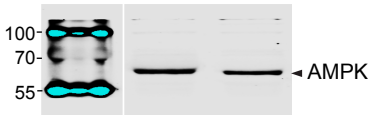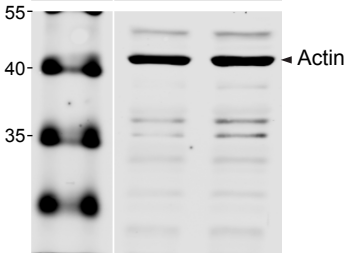

Supplement: Figure 5—figure supplement 1—source data 1. [file elife-91002-fig5-figsupp1-data1.zip › Figure 5 - Figure supplement1 - Source data 1/Figure 5 - Figure supplement1A_uncropped.pdf]

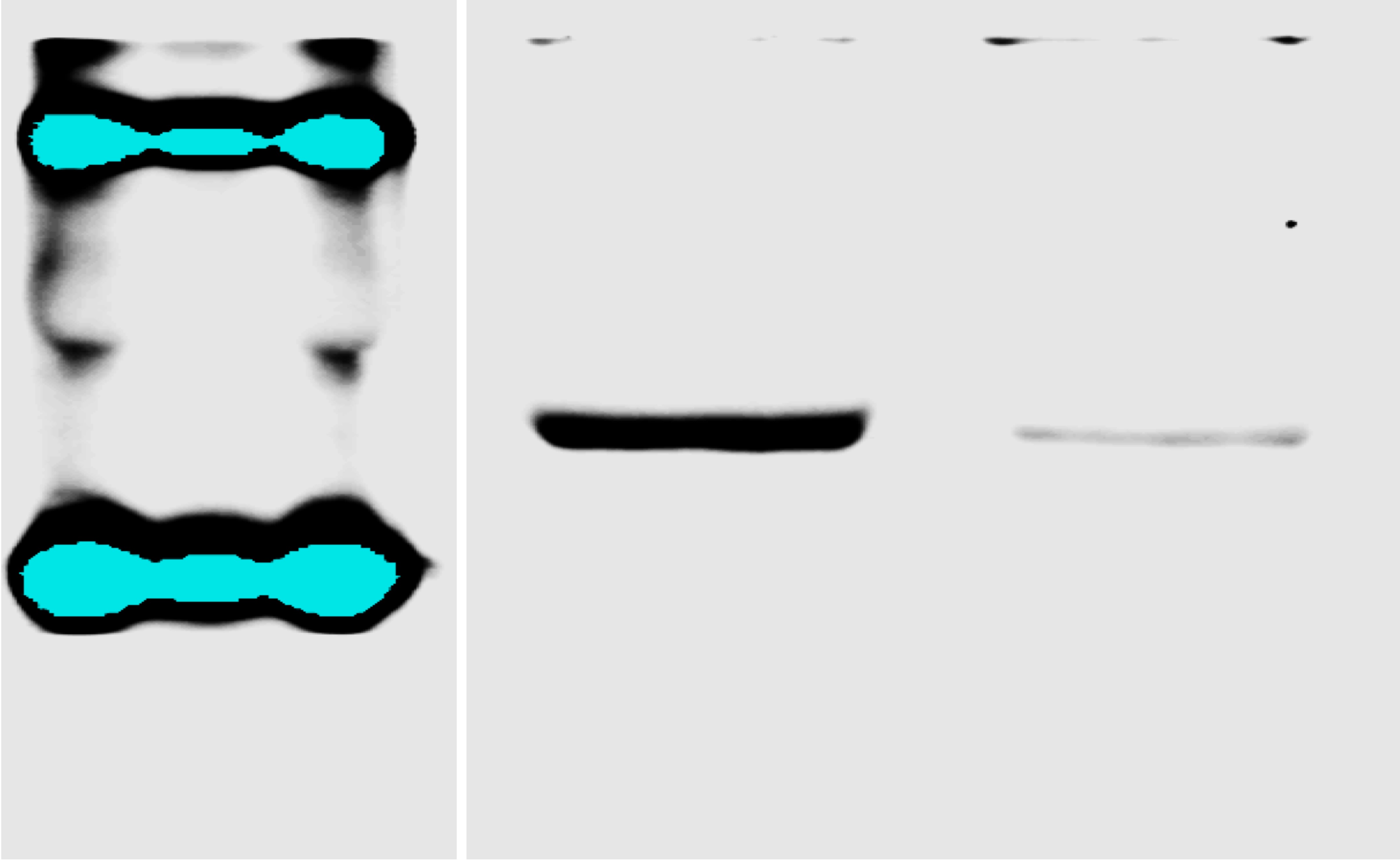

Supplement: Figure 5—figure supplement 1—source data 1. [file elife-91002-fig5-figsupp1-data1.zip › Figure 5 - Figure supplement1 - Source data 1/Figure 5 - Figure supplement1A - Source data_anti-pAMPK_raw data.jpg]

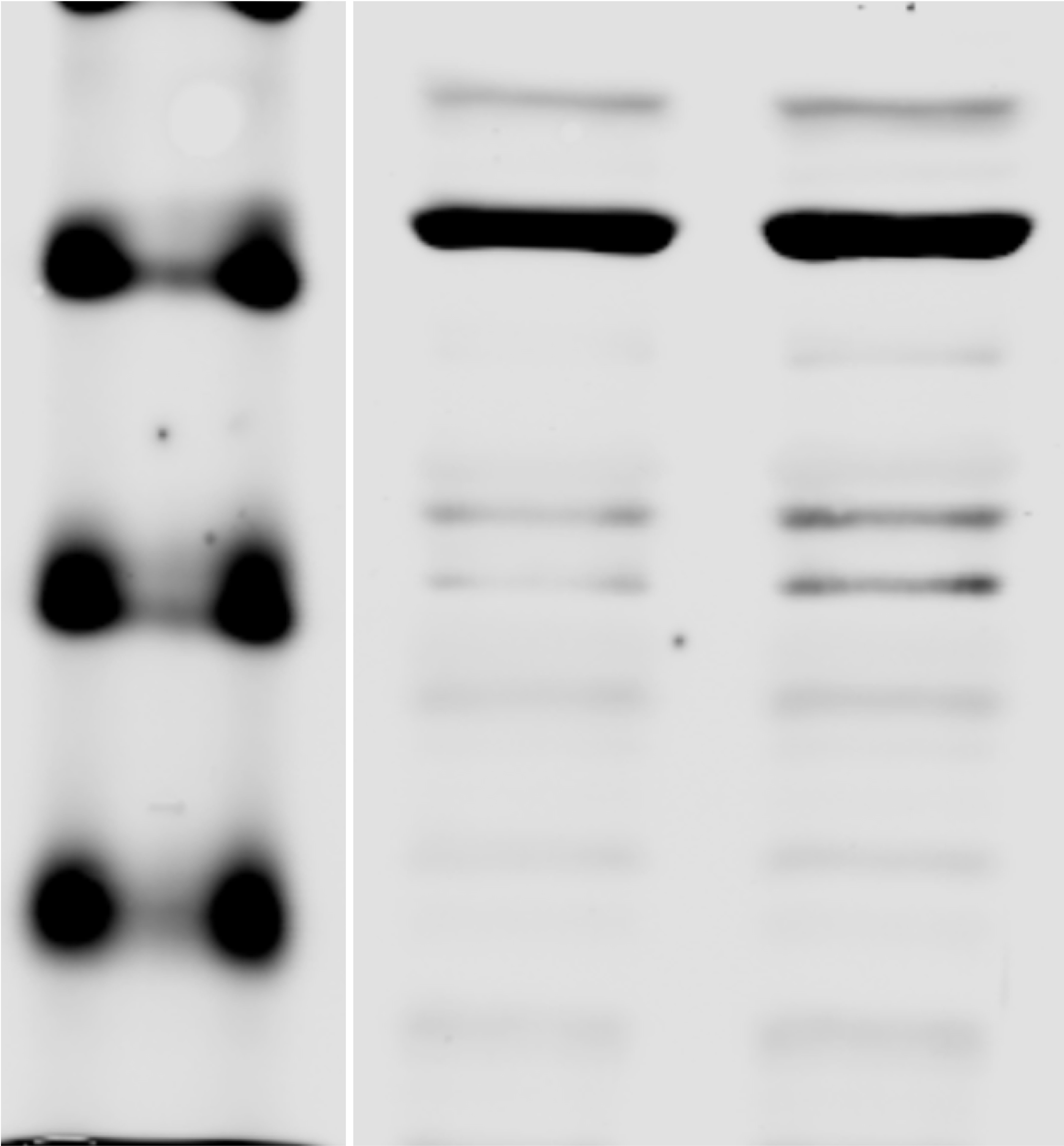

Supplement: Figure 5—figure supplement 1—source data 1. [file elife-91002-fig5-figsupp1-data1.zip › Figure 5 - Figure supplement1 - Source data 1/Figure 5 - Figure supplement1A - Source data_anti-Actin_raw data.jpg]

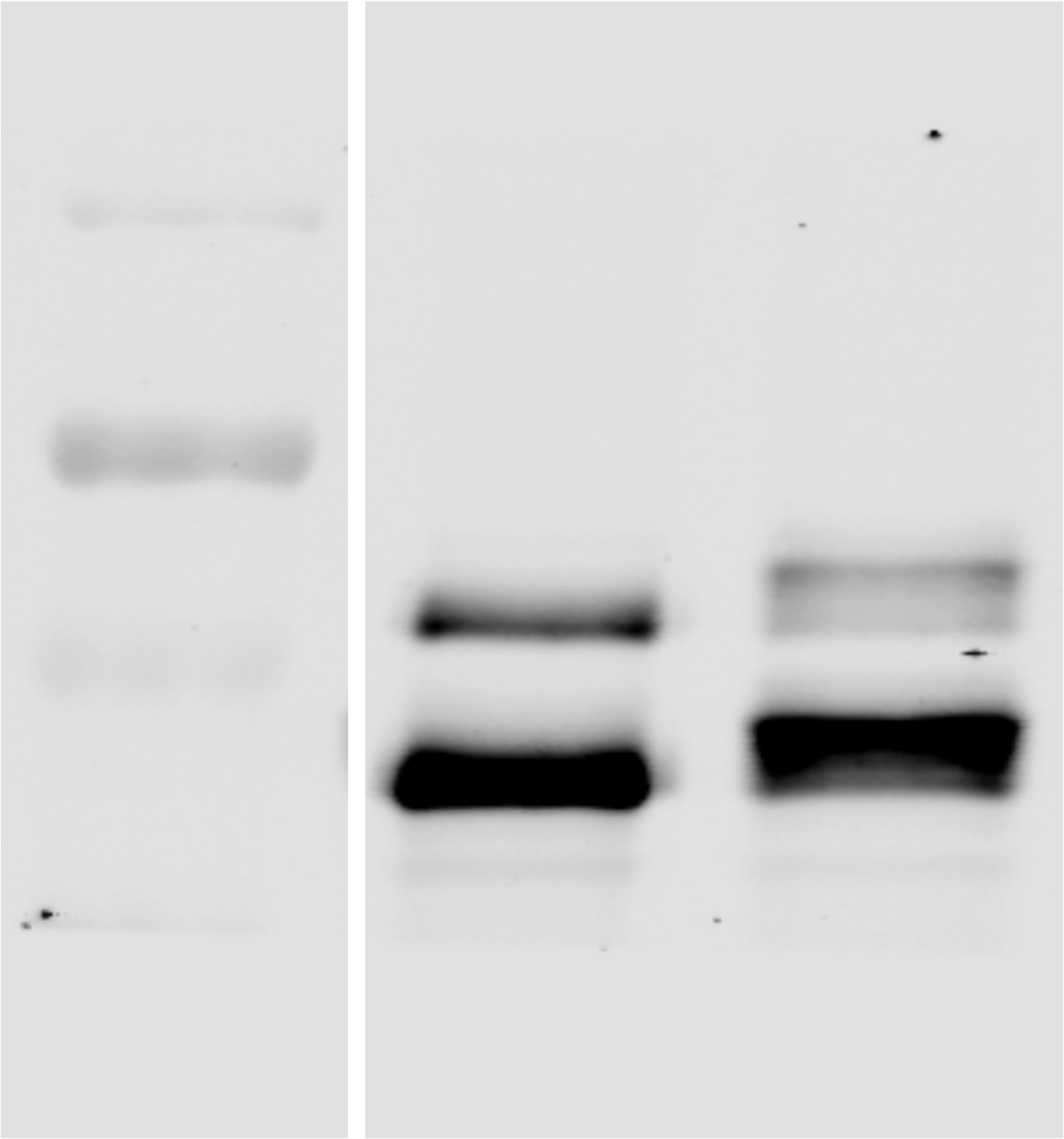

Supplement: Figure 5—figure supplement 1—source data 2. [file elife-91002-fig5-figsupp1-data2.zip › Figure 5 - Figure supplement1 - Source data 2/Figure 5 - Figure supplement1E - Source data_anti-S6K_raw data.jpg]

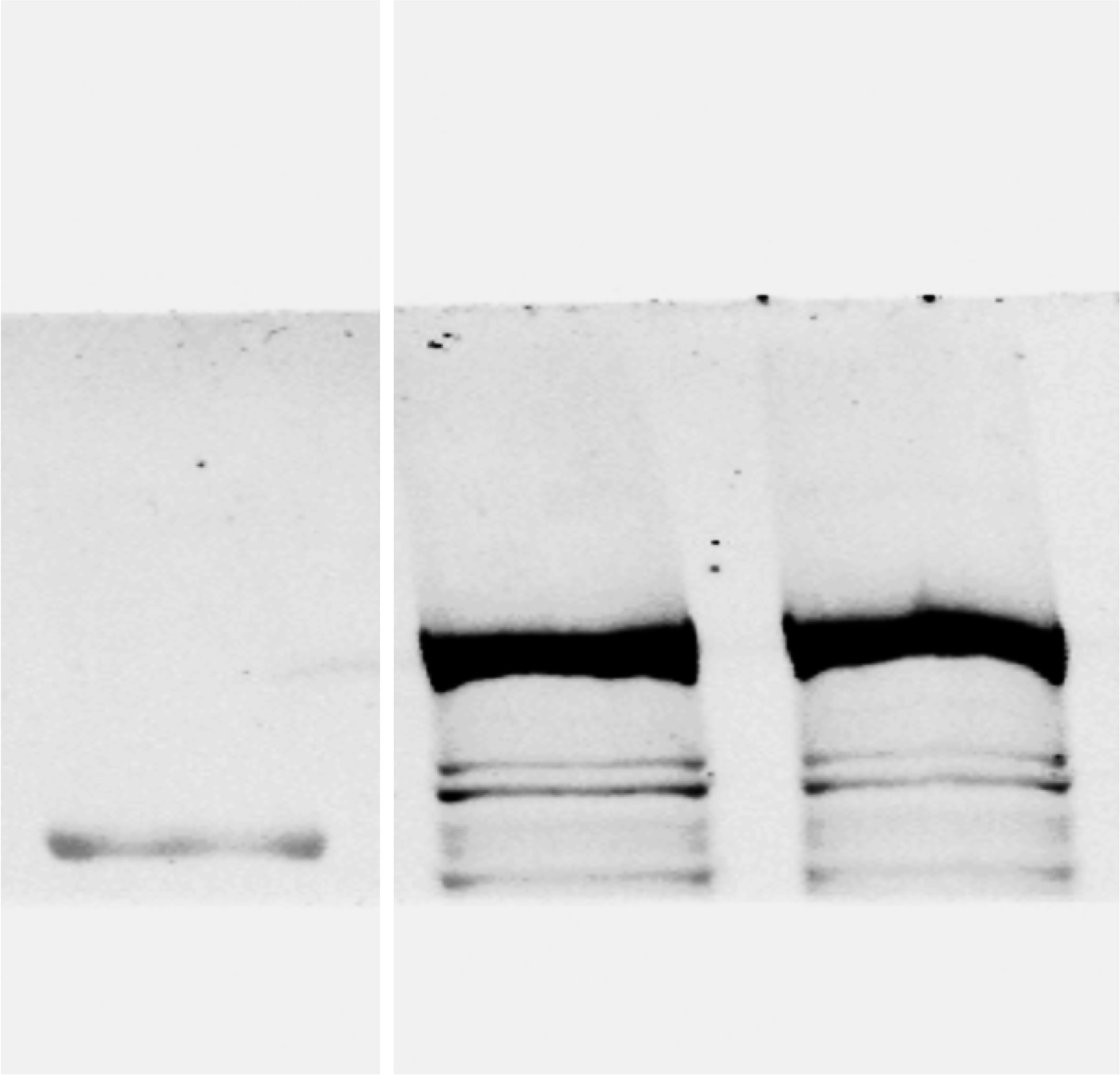

Supplement: Figure 5—figure supplement 1—source data 2. [file elife-91002-fig5-figsupp1-data2.zip › Figure 5 - Figure supplement1 - Source data 2/Figure 5 - Figure supplement1E - Source data_anti-mTOR_raw data.jpg]

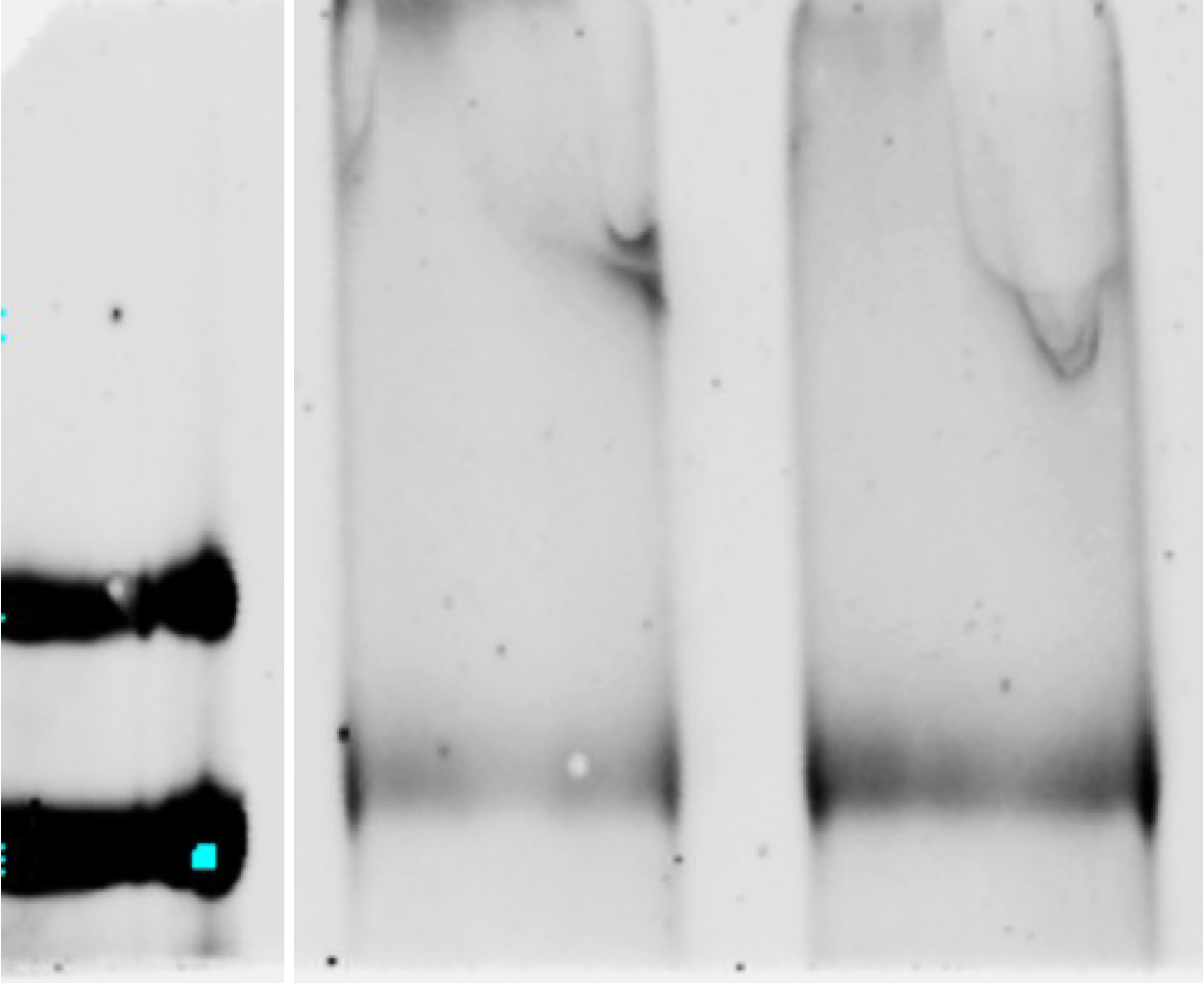

Supplement: Figure 5—figure supplement 1—source data 2. [file elife-91002-fig5-figsupp1-data2.zip › Figure 5 - Figure supplement1 - Source data 2/Figure 5 - Figure supplement1E - Source data_anti-ULK1_raw data.jpg]

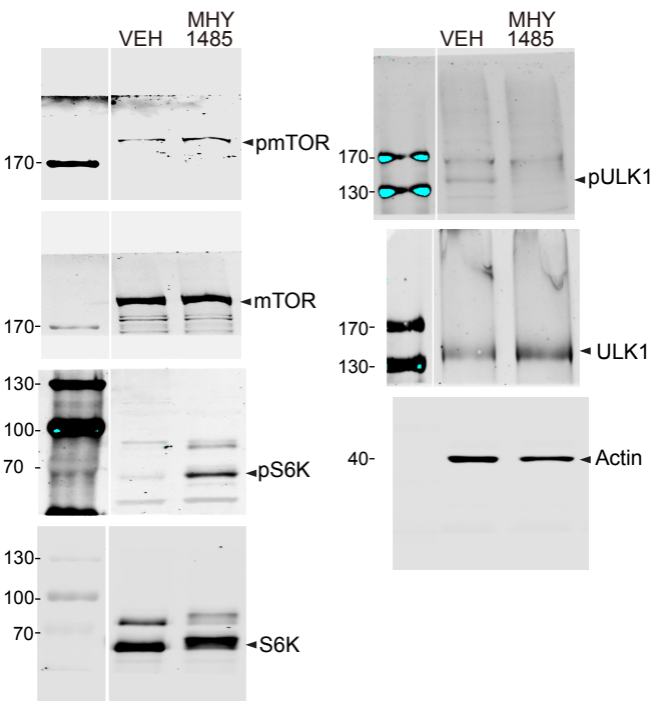

Supplement: Figure 5—figure supplement 1—source data 2. [file elife-91002-fig5-figsupp1-data2.zip › Figure 5 - Figure supplement1 - Source data 2/Figure 5 - Figure supplement1E_uncropped.pdf]

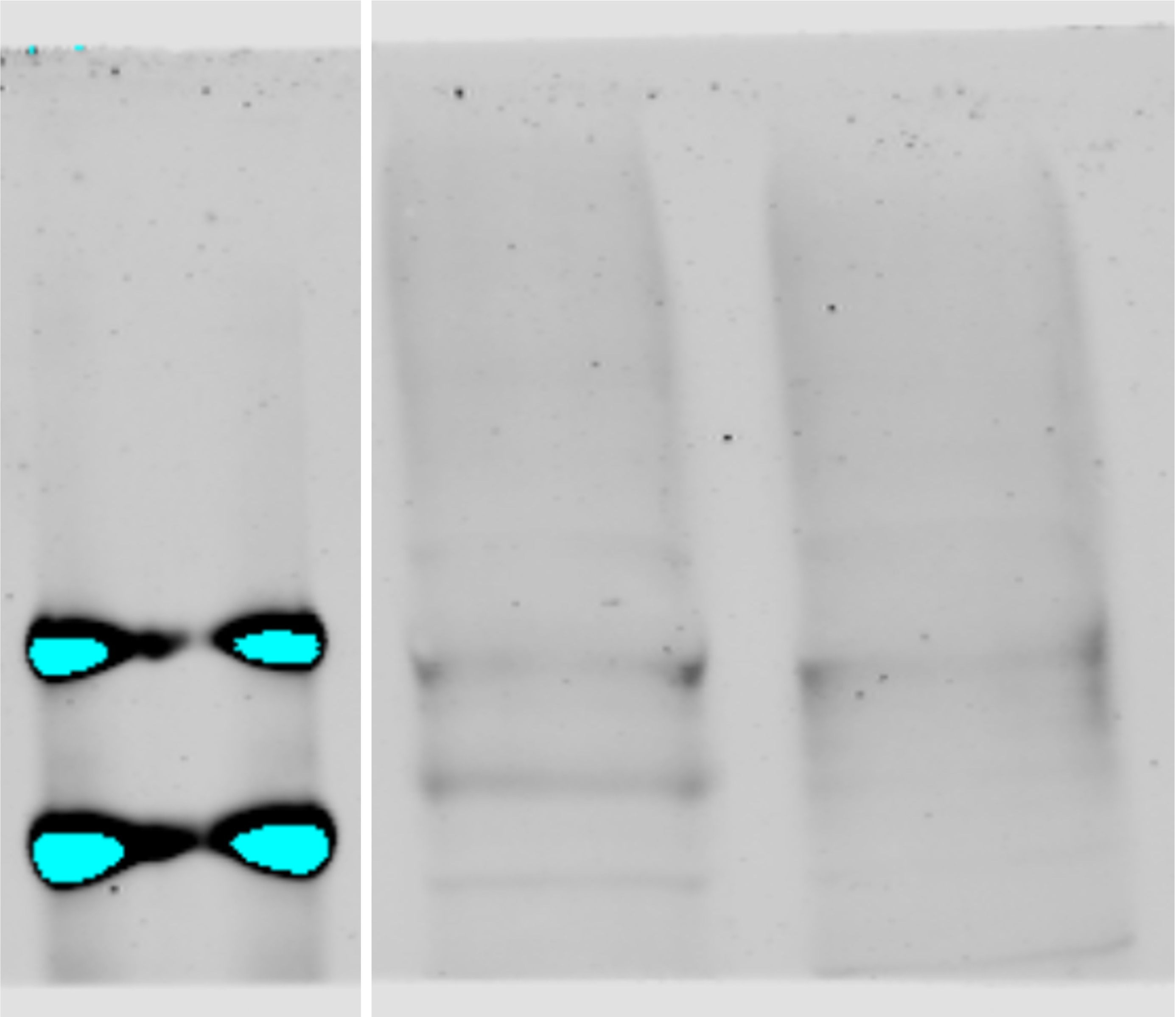

Supplement: Figure 5—figure supplement 1—source data 2. [file elife-91002-fig5-figsupp1-data2.zip › Figure 5 - Figure supplement1 - Source data 2/Figure 5 - Figure supplement1E - Source data_anti-pULK1_raw data.jpg]

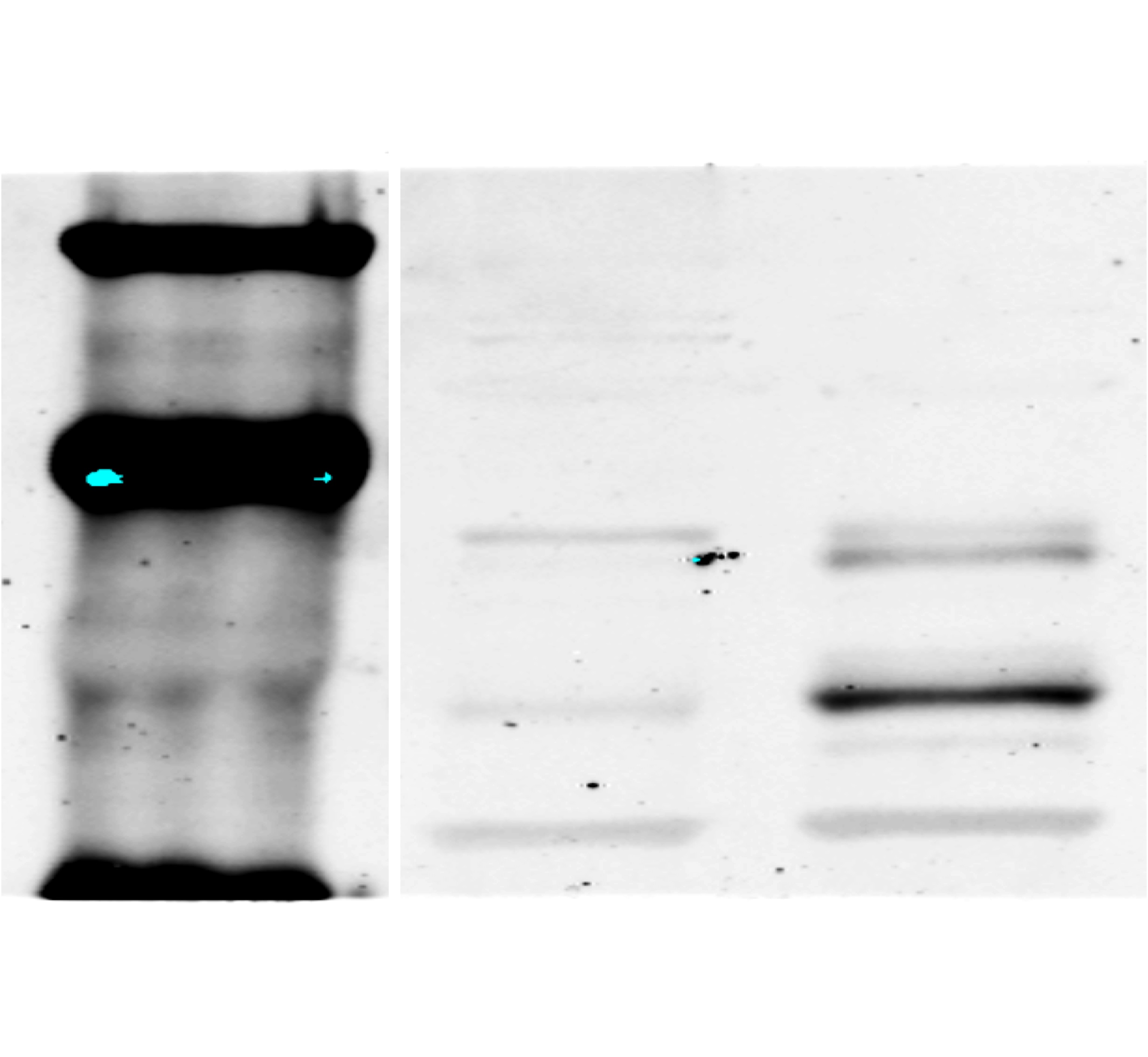

Supplement: Figure 5—figure supplement 1—source data 2. [file elife-91002-fig5-figsupp1-data2.zip › Figure 5 - Figure supplement1 - Source data 2/Figure 5 - Figure supplement1E - Source data_anti-pS6K_raw data.jpg]

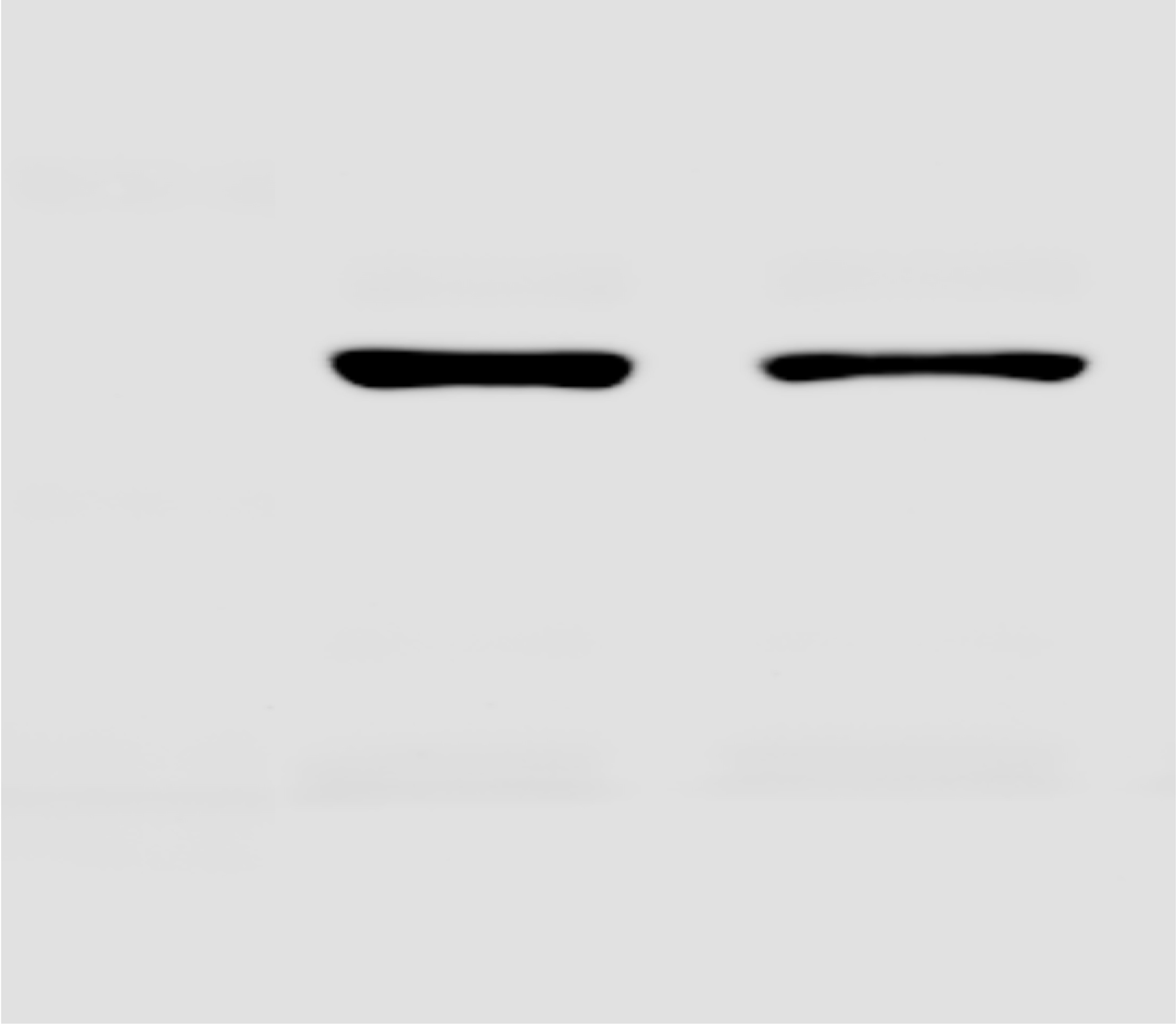

Supplement: Figure 5—figure supplement 1—source data 2. [file elife-91002-fig5-figsupp1-data2.zip › Figure 5 - Figure supplement1 - Source data 2/Figure 5 - Figure supplement1E - Source data_anti-Actin_raw data.jpg]

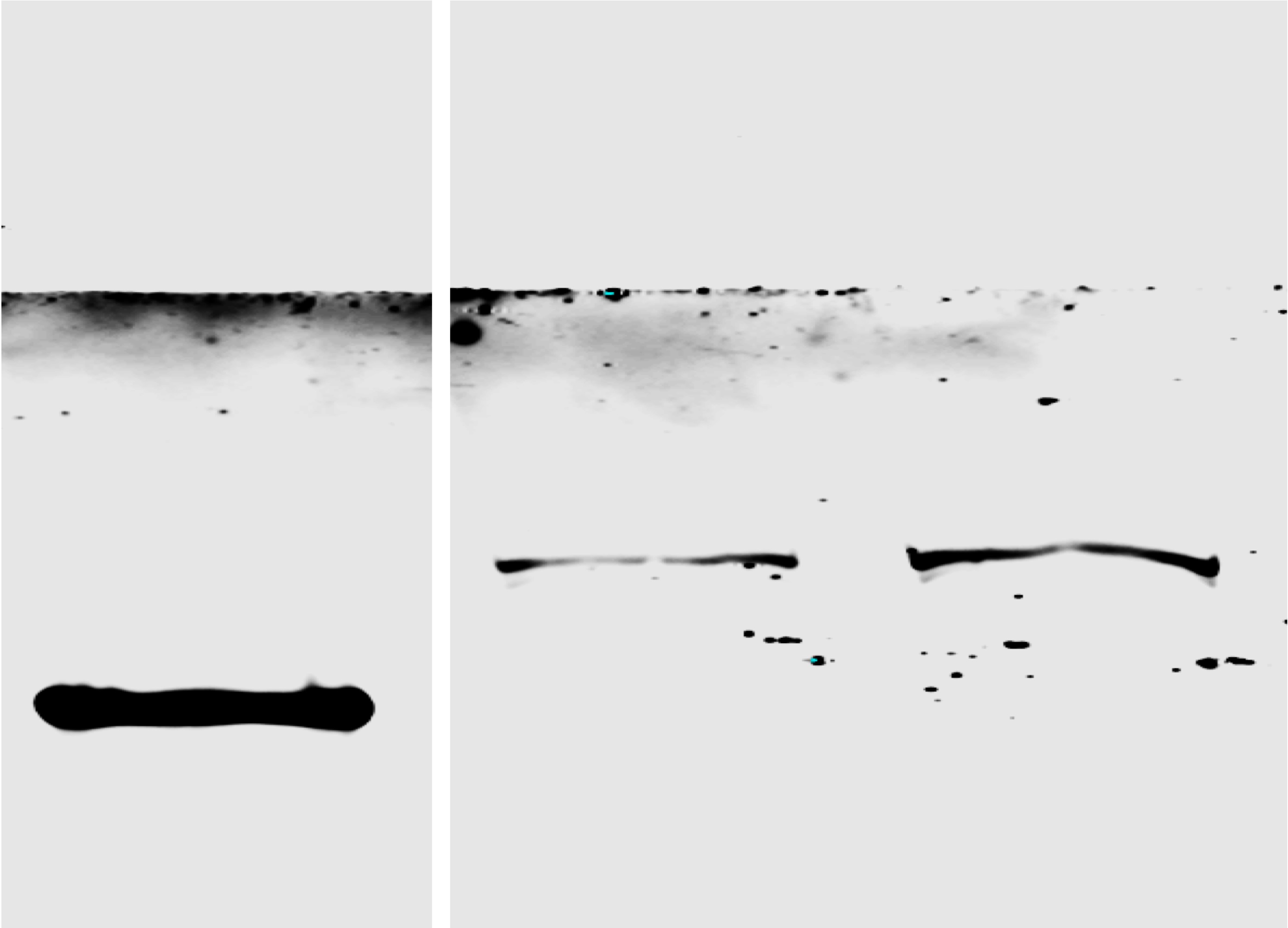

Supplement: Figure 5—figure supplement 1—source data 2. [file elife-91002-fig5-figsupp1-data2.zip › Figure 5 - Figure supplement1 - Source data 2/Figure 5 - Figure supplement1E - Source data_anti-pmTOR_raw data.jpg]

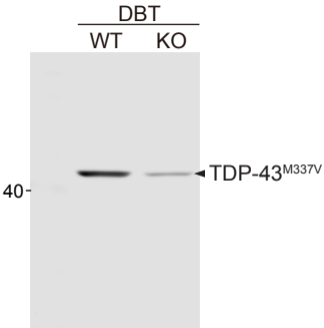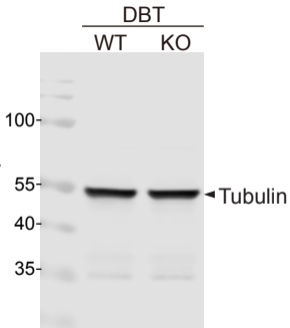

Supplement: Figure 6—source data 1. [file elife-91002-fig6-data1.zip › Figure 6 - Source data 1/Figure_6C_uncropped.pdf]

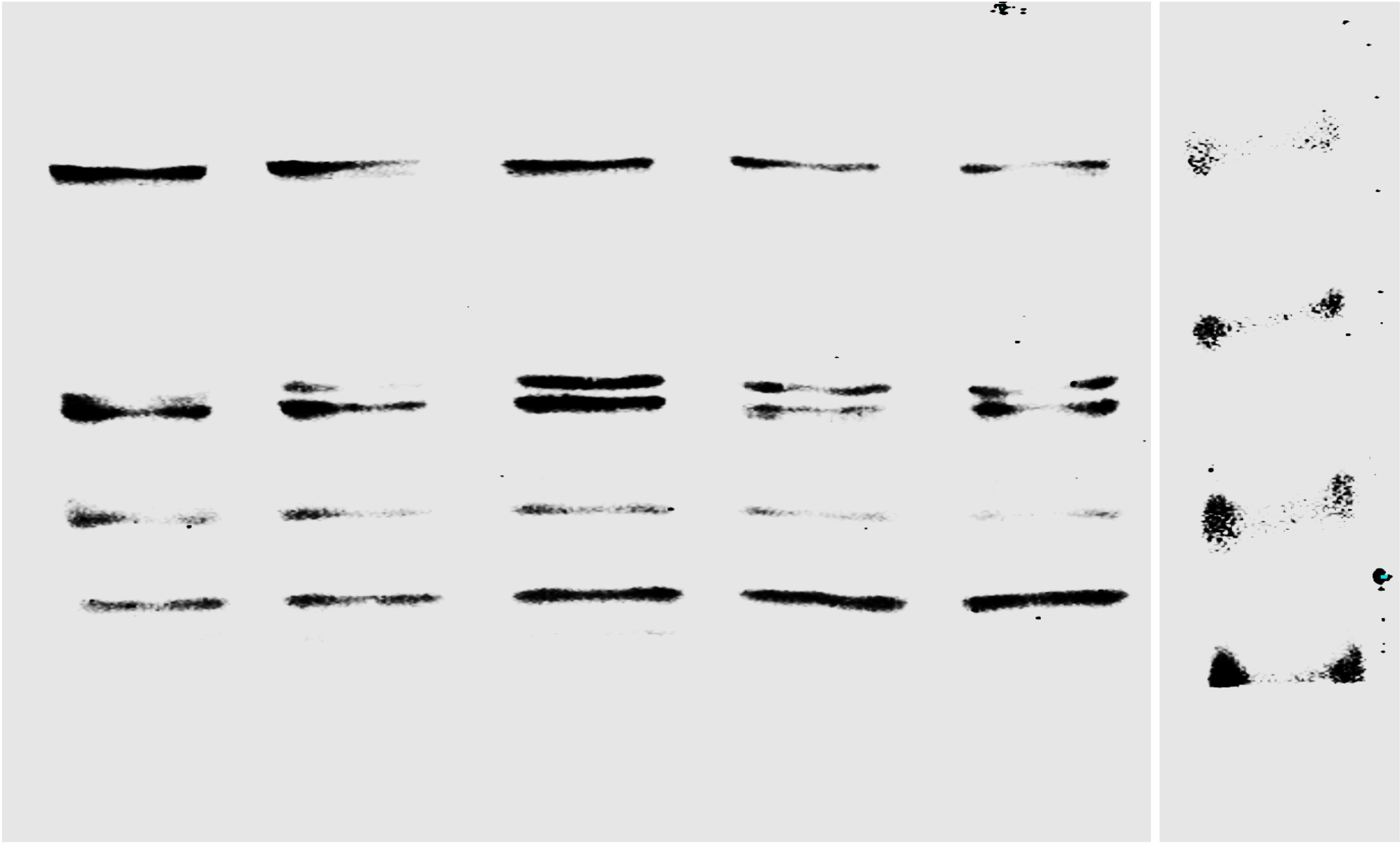

Supplement: Figure 6—source data 2. [file elife-91002-fig6-data2.zip › Figure 6 - Source data 2/Figure_6D- Source data_anti-TDP43_raw data2.jpg]

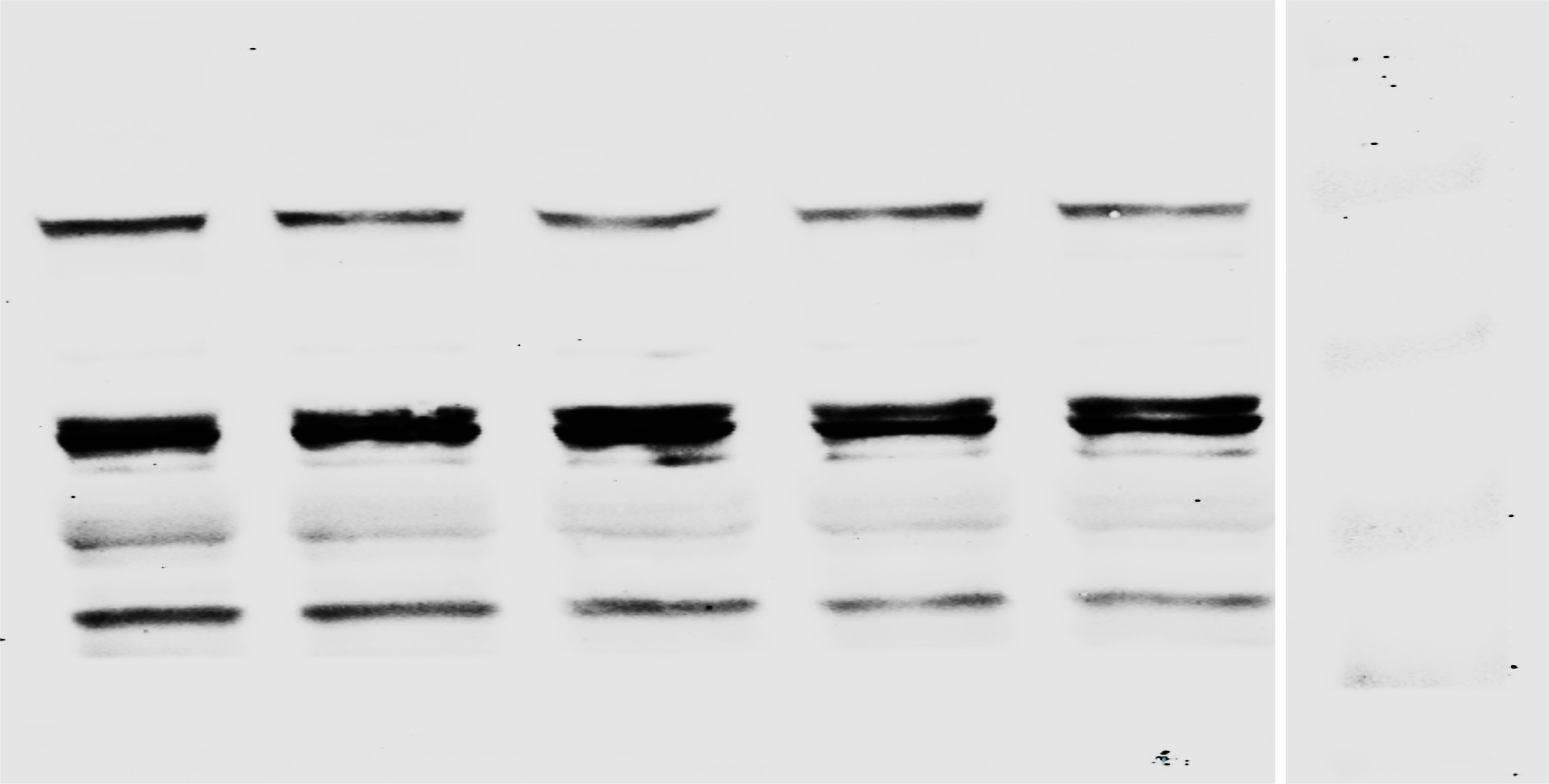

Supplement: Figure 6—source data 2. [file elife-91002-fig6-data2.zip › Figure 6 - Source data 2/Figure_6D- Source data_anti-TDP43_raw data.jpg]

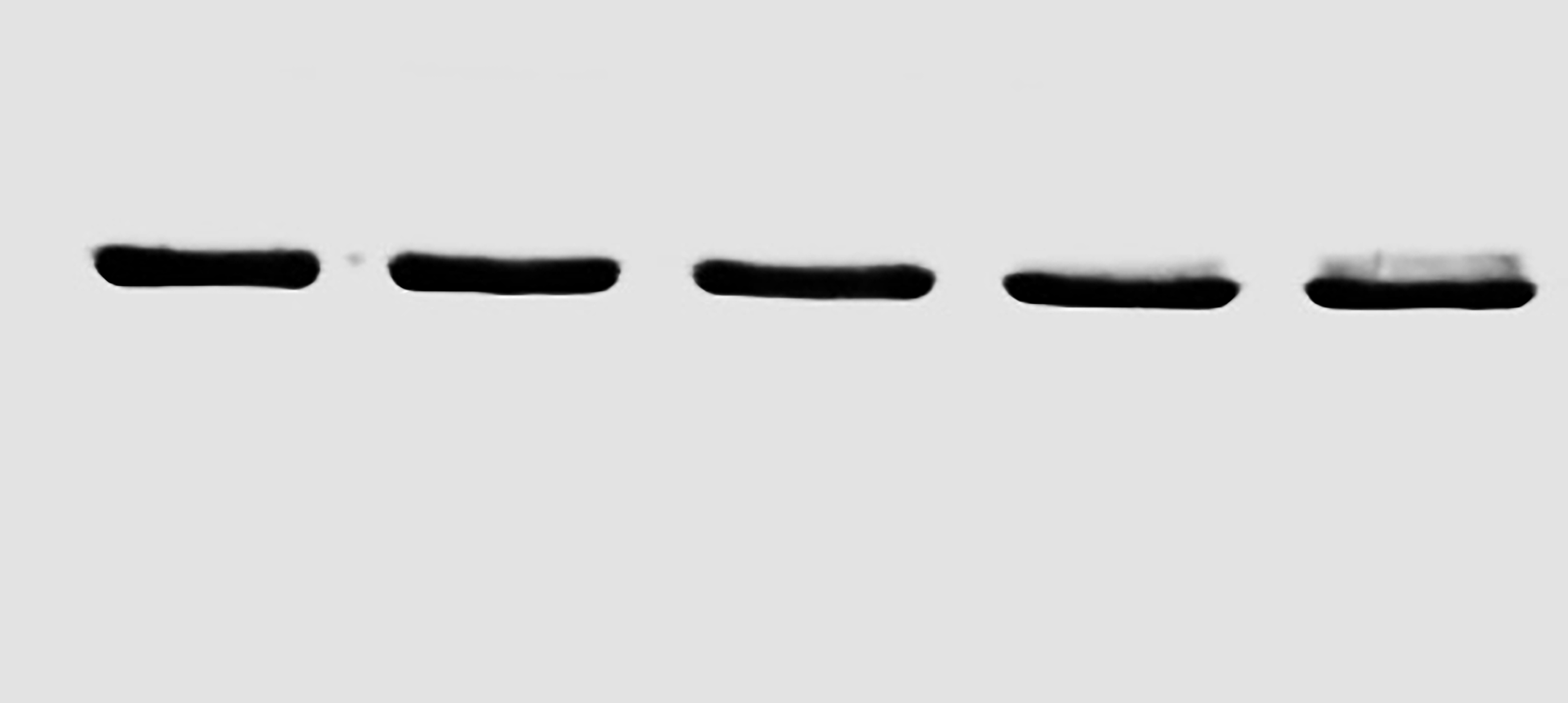

Supplement: Figure 6—source data 2. [file elife-91002-fig6-data2.zip › Figure 6 - Source data 2/Figure_6D- Source data_anti-Actin_raw data.jpg]

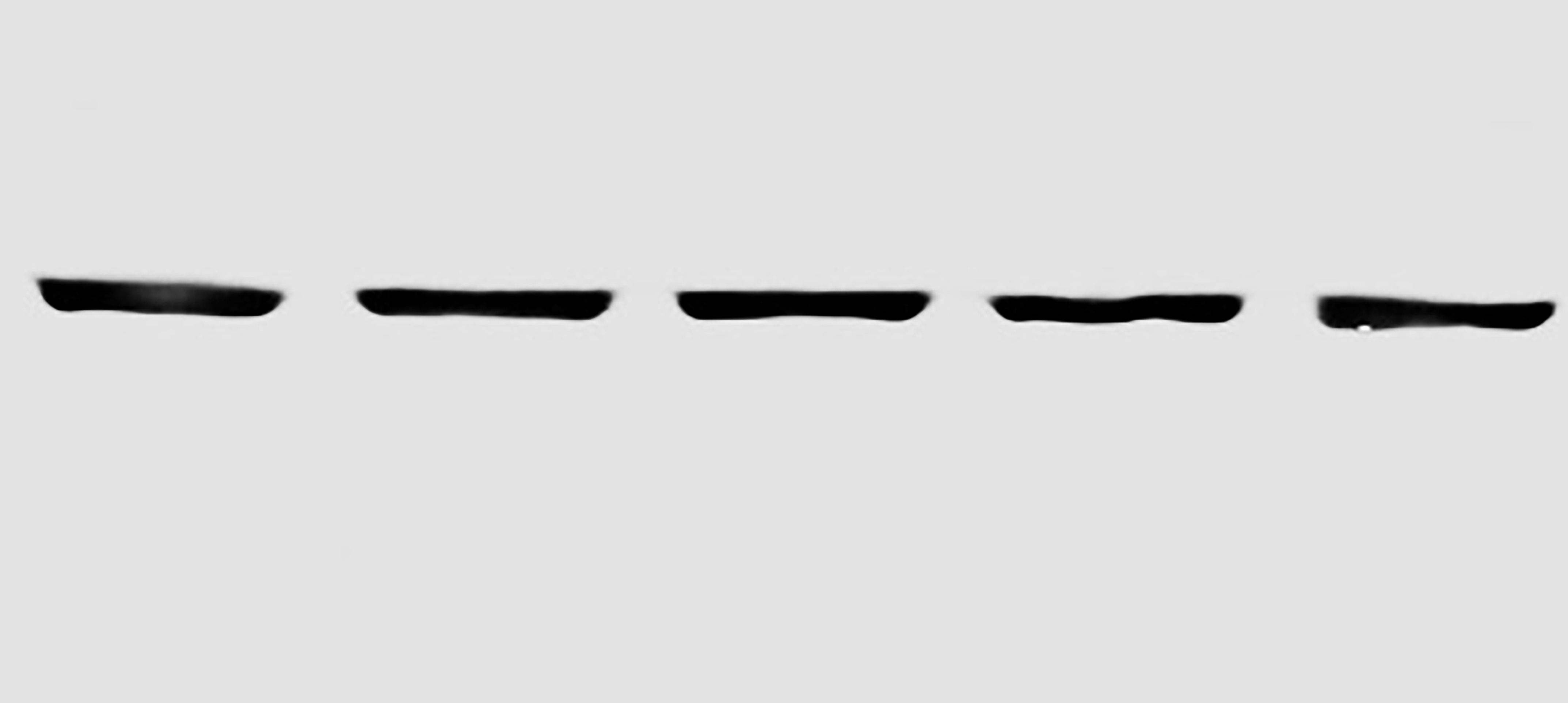

Supplement: Figure 6—source data 2. [file elife-91002-fig6-data2.zip › Figure 6 - Source data 2/Figure_6D- Source data_anti-Actin_raw data2.jpg]

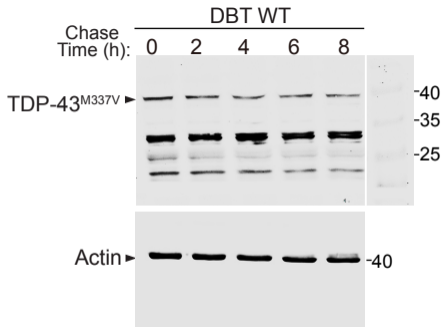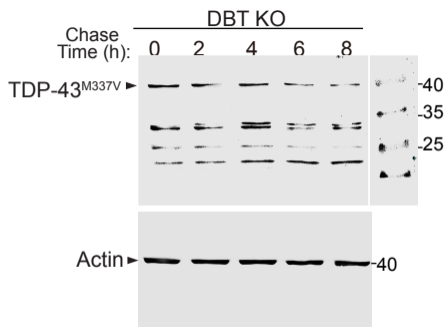

Supplement: Figure 6—source data 2. [file elife-91002-fig6-data2.zip › Figure 6 - Source data 2/Figure_6D_uncropped.pdf]

|        |  | myc-TDP-43 <sup>M337V</sup> |   |    |   |
|--------|--|-----------------------------|---|----|---|
| DBT:   |  | WT                          |   | KO |   |
| Baf A1 |  | -                           | + | -  | + |

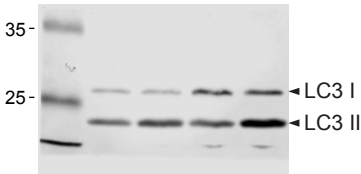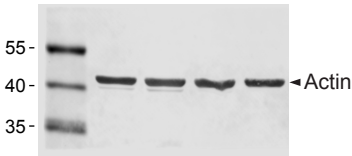

Supplement: Figure 6—source data 3. [file elife-91002-fig6-data3.zip › Figure 6 - Source data 3/Figure_6E_uncropped.pdf]

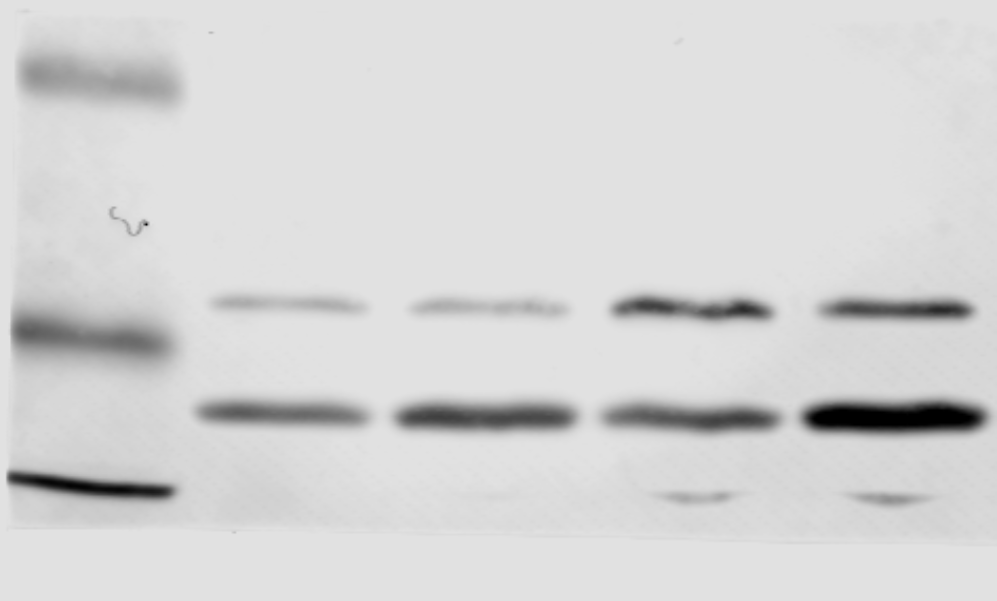

Supplement: Figure 6—source data 3. [file elife-91002-fig6-data3.zip › Figure 6 - Source data 3/Figure_6E- Source data_anti-LC3_raw data.tif]

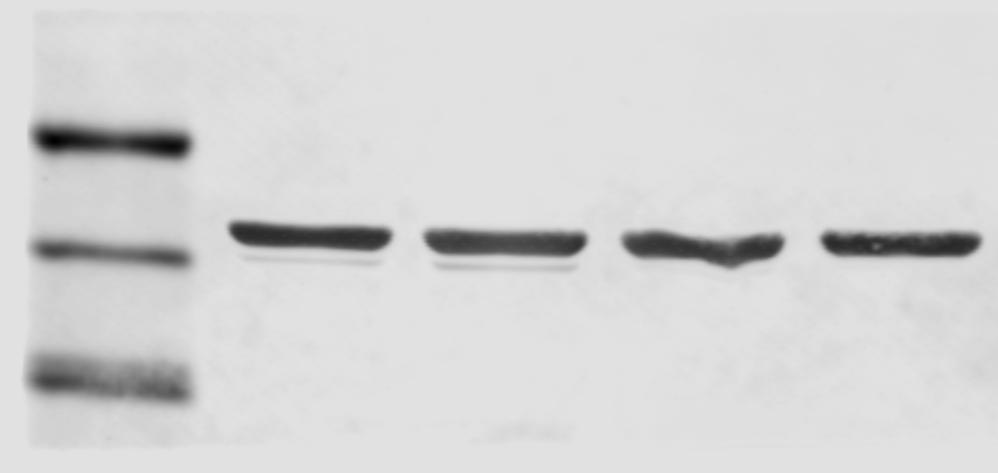

Supplement: Figure 6—source data 3. [file elife-91002-fig6-data3.zip › Figure 6 - Source data 3/Figure_6E- Source data_anti-Actin_raw data.tif]

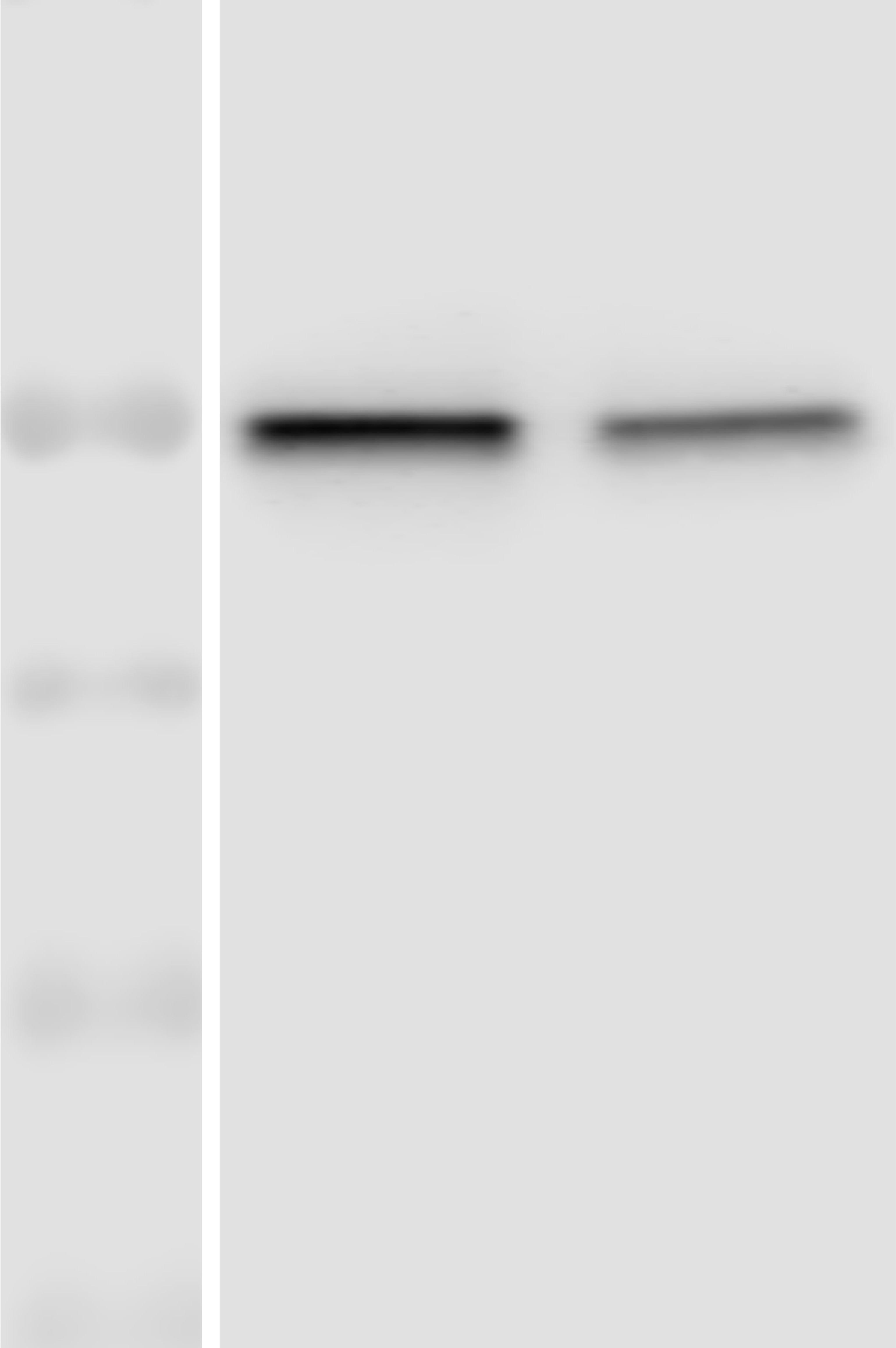

Supplement: Figure 6—figure supplement 1—source data 1. [file elife-91002-fig6-figsupp1-data1.zip › Figure 6 - Figure supplement1 - Source data 1/Figure 6 - Figure supplement1C - Source data_anti-polyQ_raw data.jpg]

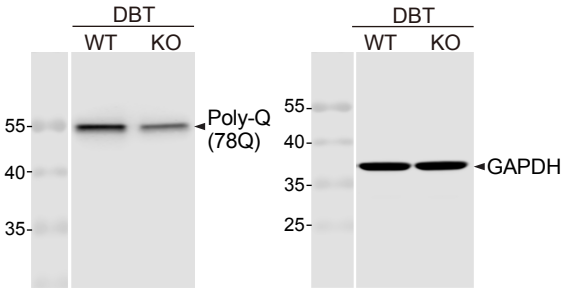

Supplement: Figure 6—figure supplement 1—source data 1. [file elife-91002-fig6-figsupp1-data1.zip › Figure 6 - Figure supplement1 - Source data 1/Figure 6 - Figure supplement1C_uncropped.pdf]

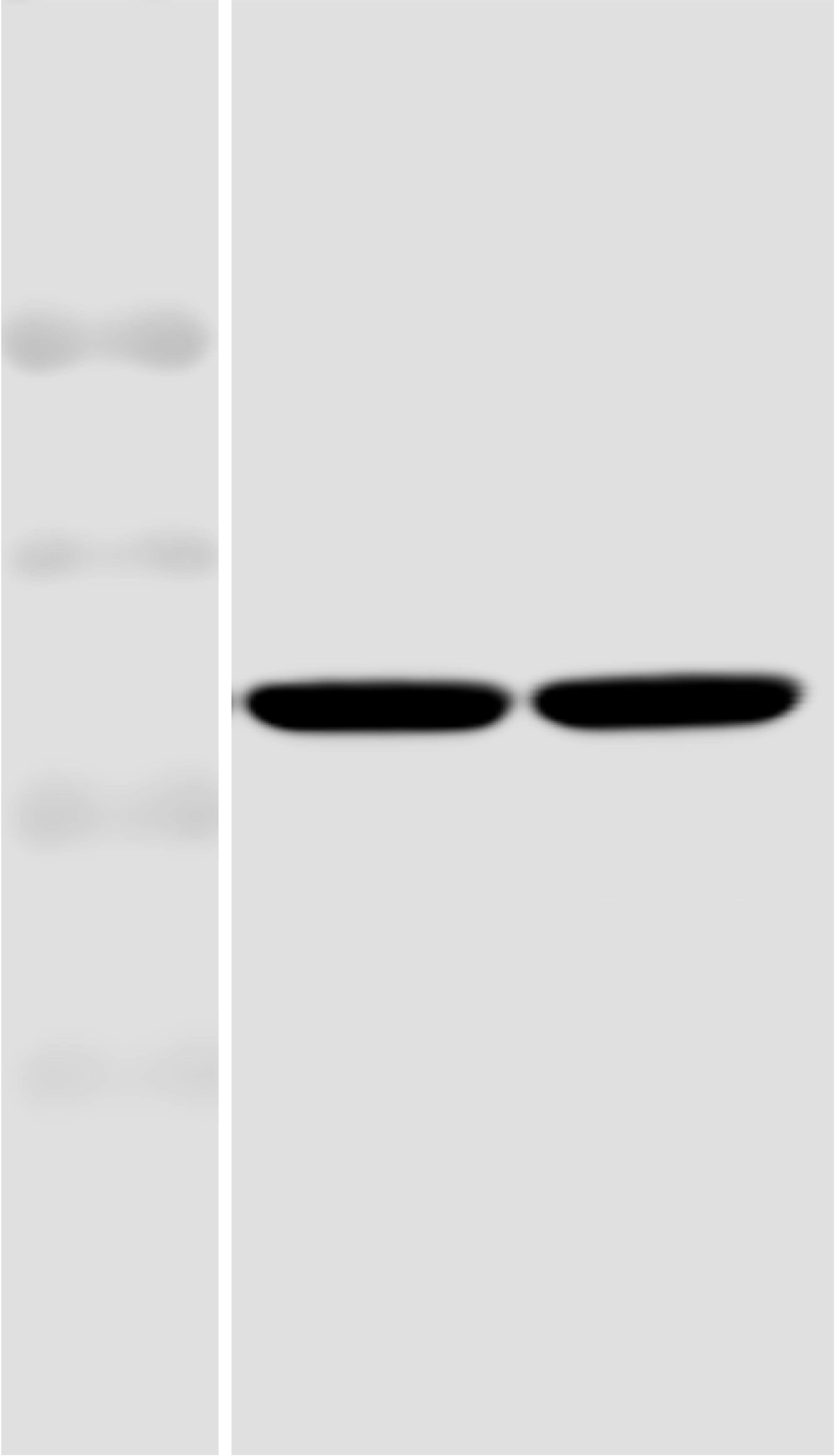

Supplement: Figure 6—figure supplement 1—source data 1. [file elife-91002-fig6-figsupp1-data1.zip › Figure 6 - Figure supplement1 - Source data 1/Figure 6 - Figure supplement1C - Source data_anti-GAPDH_raw data.jpg]

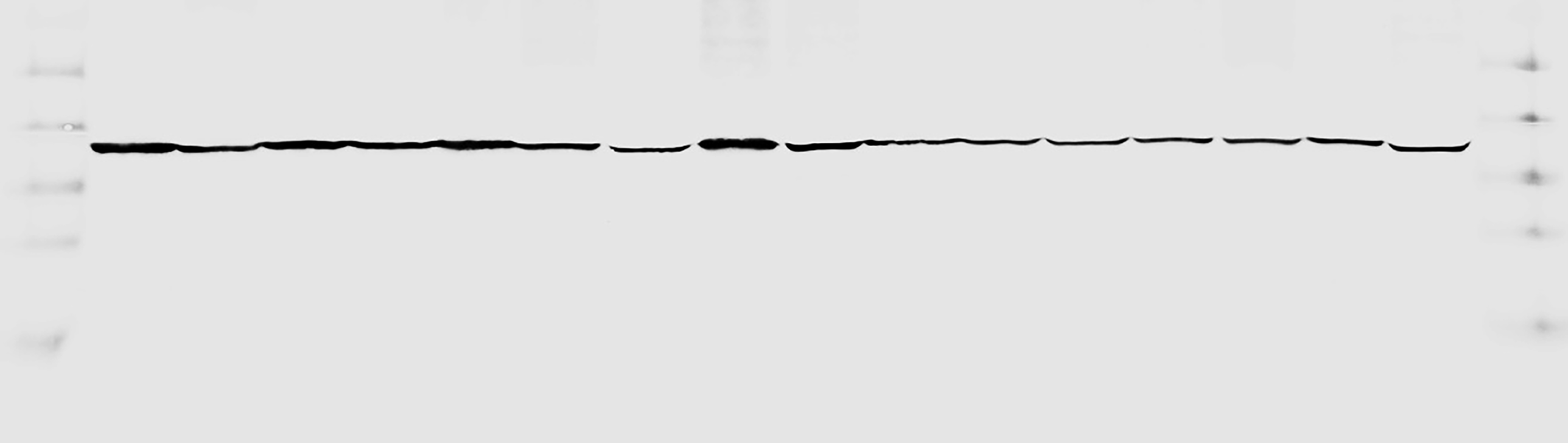

Supplement: Figure 7—source data 1. [file elife-91002-fig7-data1.zip › Figure 7 - Source data 1/Figure_7A- Source data_anti-GAPDH_raw data.jpg]
