# Supplementary figures and images for: DBT is a metabolic switch for maintenance of proteostasis under proteasomal impairment (part 2 of 2)
Source: eLife. 2024 Sep 10;12:RP91002. doi: 10.7554/eLife.91002 (PMC11386957; doi:10.7554/eLife.91002)

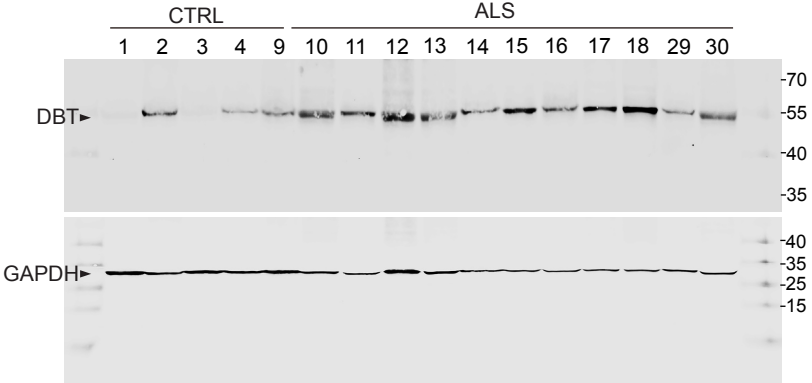

Supplement: Figure 7—source data 1. [file elife-91002-fig7-data1.zip › Figure 7 - Source data 1/Figure_7A_uncropped.pdf]

DBT : WT      KO  
TDP-43<sup>M337V</sup> : -    +    -    +

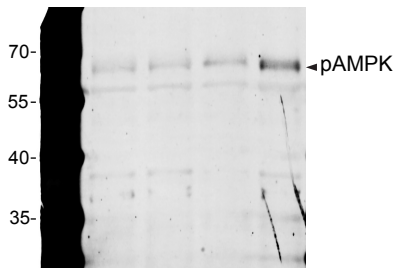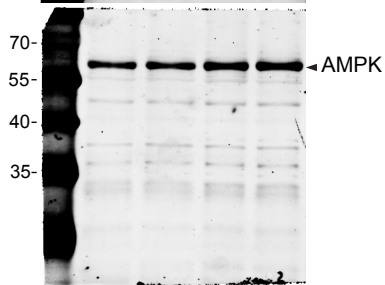

DBT : WT      KO  
TDP-43<sup>M337V</sup> : -    +    -    +

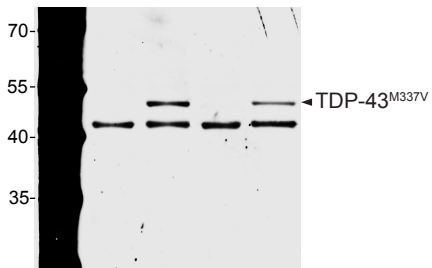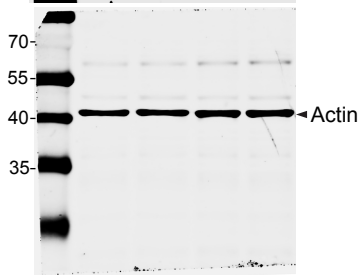

Supplement: Figure 7—figure supplement 1—source data 1. [file elife-91002-fig7-figsupp1-data1.zip › Figure 7 - Figure supplement1 - Source data 1/Figure 7 - Figure supplement1A_uncropped.pdf]

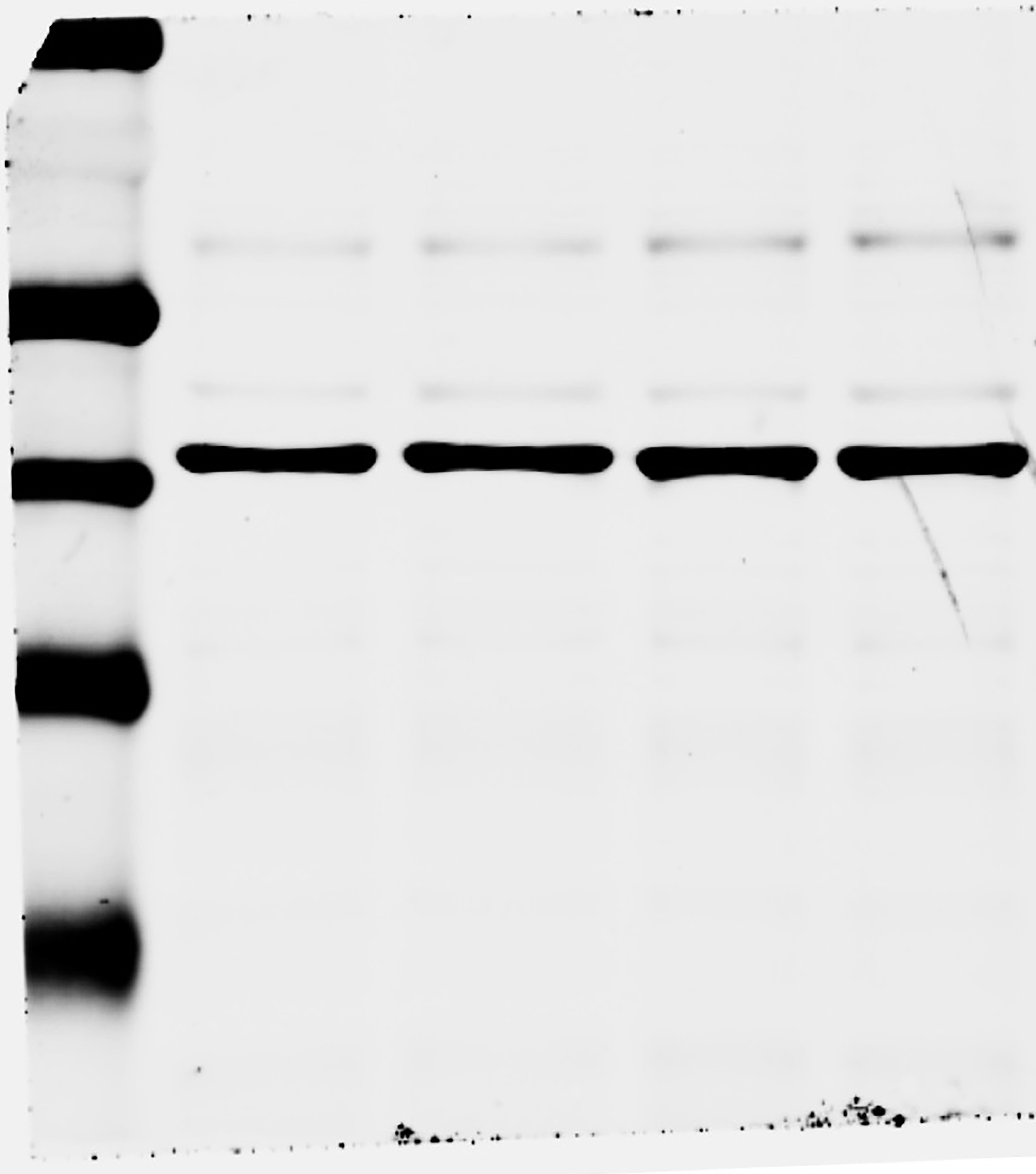

Supplement: Figure 7—figure supplement 1—source data 1. [file elife-91002-fig7-figsupp1-data1.zip › Figure 7 - Figure supplement1 - Source data 1/Figure 7 - Figure supplement1A - Source data_anti-Actin_raw data.jpg]

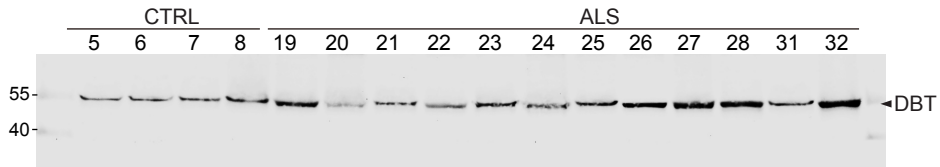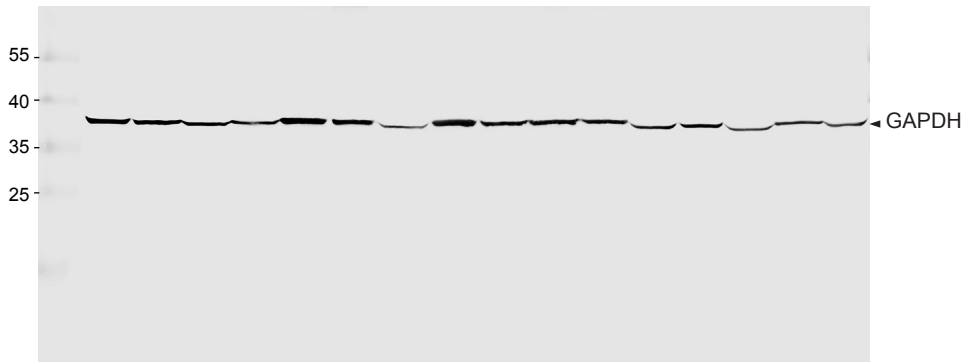

Supplement: Figure 7—figure supplement 1—source data 2. [file elife-91002-fig7-figsupp1-data2.zip › Figure 7 - Figure supplement1 - Source data 2/Figure 7 - Figure supplement1B_uncropped.pdf]

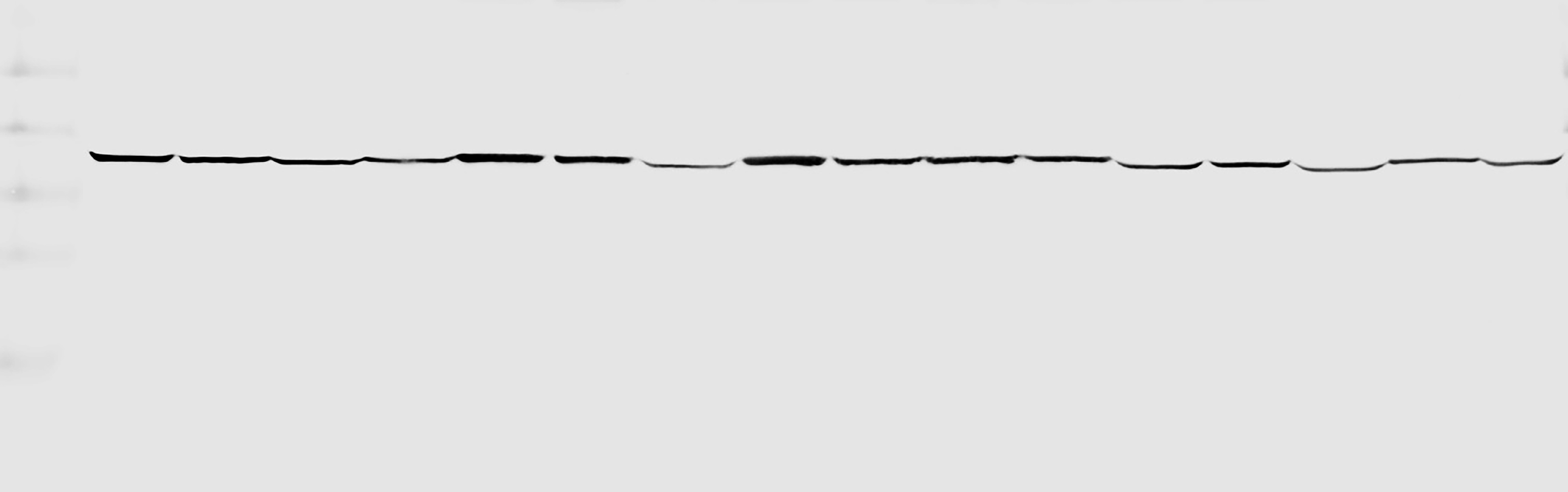

Supplement: Figure 7—figure supplement 1—source data 2. [file elife-91002-fig7-figsupp1-data2.zip › Figure 7 - Figure supplement1 - Source data 2/Figure 7 - Figure supplement1B - Source data_anti-GAPDH_raw data.jpg]
